# Examining varying number of intervention-modules and change in individual networks in young people remitted from depression or anxiety: Exploratory outcomes of the StayFine randomized clinical trial

**DOI:** 10.1016/j.nsa.2026.107007

**Published:** 2026-04-30

**Authors:** S.J. Robberegt, C.J. Albers, B.E.A.M. Kooiman, A.H. Vuuregge, J.D. Mul, N. Wilts, M.H. Nauta, Y.A.J. Stikkelbroek, C.L.H. Bockting

**Affiliations:** a Amsterdam UMC, Department of Psychiatry, Amsterdam Public Health, Amsterdam, the Netherlands; b Depression Expertise Centre-Youth, GGZ Oost Brabant, Boekel, the Netherlands; c Department of Psychometrics & Statistics, Faculty of Behavioral and Social Sciences, University of Groningen, Groningen, the Netherlands; d Department of Clinical Psychology and Experimental Psychopathology, Faculty of Behavioral and Social Sciences, University of Groningen, Groningen, the Netherlands; e Centre for Urban Mental Health, University of Amsterdam, Amsterdam, the Netherlands; f Swammerdam Institute for Life Sciences, Faculty of Science, University of Amsterdam, Amsterdam, the Netherlands; g Accare Child Study Centre, Groningen, the Netherlands; h Department of Clinical Child and Family Studies, Faculty of Social and Behavioral Sciences, Utrecht University, Utrecht, the Netherlands

**Keywords:** Digital interventions, Relapse prevention, Ecological momentary assessment, Network approach, Depressive disorders, Anxiety disorders

## Abstract

In network theory, symptoms of mental disorders constitute networks of interconnected nodes. Interventions can target (aspects of) networks. Assuming that combined intervention-modules target multiple aspects of networks simultaneously, we examined on average whether receiving more intervention-modules was associated with more baseline to post-intervention change in network characteristics. Between Dec-2019 and May-2024, 14-to-22-year-old individuals (89.8% female) remitted from depressive or anxiety disorders were randomized (1:1) to relapse prevention intervention-modules plus care as usual (M + CAU; *n* = 69), or CAU (*n* = 68). Participants completed baseline and post-intervention ecological momentary assessment (EMA; 2 weeks, 6 × /day) to construct contemporaneous partial correlation networks of 11 nodes (*anxiety, sadness, positive affect, anger, stress, fatigue, suppression, avoidance, loneliness, activity, social company*). Based on a data-driven advice, including the baseline network, six intervention-modules were offered in personalized varying combinations (psychoeducation, cognitive restructuring, positive affect, exposure, activation, sleep, wellness, relapse prevention plan). Using 45 univariate regression analyses (*α =* .05), associations were assessed between the number of completed intervention-modules (M[SD] = 4.29[1.70]; range = 0-6) and change in network density, and in intensity, instability, and centrality of 11 nodes. Randomization groups were compared using two-sample t-tests.

Results indicated that after multiple testing correction, a higher number of intervention-modules was not associated with significant change in network characteristics. Changes in networks were neither different between randomization groups. Some characteristics showed non-robust, small associations with a higher number of intervention-modules: centrality of *fatigue* decreased (*n* = 69;*β* = −0.061, 95%CI [-0.122,0.000], *p* = .037, *η*_*p*_^*2*^ = 0.03), and network density decreased (*n* = 69; *β* = −0.008, 95%CI [-0.015,0.000], *p* = .015, *η*_*p*_^*2*^ = 0.05). Change in centrality of *loneliness* was positive in the M + CAU-group and negative in the CAU-group (t[130.33] = -2.47, *p* = .015, d = −0.42). In conclusion, after multiple testing correction, more intervention-modules were not associated with more change in network characteristics across individuals. This initial examination provides a starting point for replication in confirmatory analyses, and for better mechanistic understanding of network change to prevent relapse over time.

## Abbreviations

CAU =: Care as usual
CBT =: Cognitive behavioral therapy
EMA =: Ecological momentary assessment
K-SADS-PL DSM-5 =: Kiddie-Schedule for Affective Disorders and Schizophrenia present and lifetime version
LASSO =: Least Absolute Shrinkage and Selection Operator regularization
M + CAU =: Intervention-modules added to care as usual
PCT =: Preventive cognitive therapy
RCT =: Randomized clinical trial

## 1 Background

Adolescents and young adults of 12 to 25 years in remission of depressive or anxiety disorders are at risk of relapse (Curry et al., 2011; Gibby et al., 2017). Estimated relapse rates vary between 20 and 72% by prior disorder, by follow-up duration, and between adolescents and young adults (Birmaher et al., 2002; Kovacs et al., 2016; Breedvelt et al., 2021a; Moffitt et al., 2010; Levy et al., 2021; Ginsburg et al., 2014; Krijnen-de Bruin et al., 2022; Bruce et al., 2005). Psychological and pharmacological relapse prevention interventions effectively reduce the risk of depressive and anxiety relapse (Robberegt et al., 2023; Breedvelt et al., 2024; Batelaan et al., 2017). Although risk remains for a substantial number of individuals; 38-60% in depression (Robberegt et al., 2023; Breedvelt et al., 2021b; Kuyken et al., 2016), and 5.2-20.1% in anxiety (Batelaan et al., 2017; White et al., 2013).

Personalization of evidence-based psychological interventions is hypothesized to improve efficacy of standardized interventions (Bennett and Shafran, 2023). Improved mental health outcomes have been reported from childhood to adulthood (e.g. Graham et al., 2020, Nye et al., 2023, Weisz et al., 2012) for personalized acute phase interventions for depression and anxiety. Furthermore, there are first indications that preventive modular interventions for adolescents are effective, also to prevent relapse (Frederick et al., 2024; Vivas-Fernandez et al., 2023; Emslie et al., 2015).

Personalization of intervention-modules can be achieved through various procedures. In cognitive therapy, a case conceptualization by the practitioner is common (ten Broeke et al., 2008). To optimise the treatment plan, it is increasingly being combined with shared decision making (Légaré and Thompson-Leduc, 2014). Innovative personalization procedures include data-driven tools that combine data from clinical interviews, self-report questionnaires, repeated assessment such as ecological momentary assessment (EMA), and devices, e.g. wearables or smartphones (Deisenhofer et al., 2024). Recently, the network approach to psychopathology (Borsboom and Cramer, 2013; Borsboom, 2017) was used to personalise interventions (Fisher et al., 2019; Sanford et al., 2022; Kooiman et al., 2023).

At the basis of the network approach to mental disorders is the assumption that symptoms trigger other symptoms, these interactions are dynamic, and symptoms and their interactions change over time (Borsboom, 2017; Epskamp et al., 2018a; Hayes et al., 2015). Individual networks contain symptoms and aspects of mental health –referred to as ‘nodes’ – and their connections –‘edges’. Individual networks are often based on repeated daily assessment of affect, thought, and behavior (i.e. EMA). Network characteristics can be extracted for each node and edge in the network (Robinaugh et al., 2020; Dejonckheere et al., 2019). Additionally, overall network strength is commonly extracted (Robinaugh et al., 2020; Dejonckheere et al., 2019). To illustrate, a higher overall network strength means that it is more likely that one node activates another. Overall network strength (or *network density*) could indicate whether someone is in a depressive or resilient state (Scheffer et al., 2024a, 2024b). For example, if feeling sad is strongly associated with feeling tired, lonely, less energetic, and negatively associated with being active, either of these feelings or behaviours may trigger other nodes in the network. In this case, it is likely that experiencing these feelings or behaviours holds the person in a depressive state. Additionally, the stability of the networks could indicate whether someone is on a tipping point from a depressive state to a resilient state (van De Leemput et al., 2014; Snippe et al., 2023; Lunansky et al., 2021). If affective or behavioural changes occur during interventions, this could rapidly change which nodes are active and how nodes interact within a network. For example, when the association between sadness and loneliness becomes weaker, and a new association emerges between positive affect and being active, the network is unstable, but a transition could occur towards a more resilient state.

Interventions that target symptoms and mental processes (Borsboom and Cramer, 2013; Cramer et al., 2010) could be associated with change in EMA-based networks. Some empirical studies that measured symptoms on a moment-to-moment basis with EMA, reported change in overall network strength and symptom-interactions during interventions (Schumacher et al., 2023; Kreiter et al., 2021; van Borkulo et al., 2015; Bosley et al., 2019), whereas change in overall network strength in response to interventions was not supported by other studies (Snippe et al., 2017; Schweren et al., 2018; Strauss et al., 2020; van der Wal et al., 2023). Possible explanations for the divergent findings in earlier studies are pre-processing steps to include nodes and edges (Bringmann, 2021), and the use of temporal networks that visualize the predicted change from one moment to the next, or contemporaneous networks that visualize the predicted associations within a timeframe (Epskamp et al., 2018b). Alternative explanations are differences in patient characteristics such as age and prior episodes (Schweren et al., 2018), or in network stability due to underlying mental states. Hence, it could differ between studies whether participants are on tipping points from one state to another (van De Leemput et al., 2014; Lunansky et al., 2021). Taken together, the application of the network approach to clinical practice is challenged by mixed results from empirical studies (Wichers et al., 2021; Mcnally, 2021).

We aimed to explore whether completion of intervention-modules is associated with change in networks. In the present study, alongside a randomized clinical trial (RCT), EMA was collected six times daily for two weeks before and after intervention-modules (Robberegt et al., 2022). In the RCT, adolescents and young adults received varying combinations of six intervention-modules to prevent relapse into depression or anxiety. The combination was personalized based on a data-driven advice and shared decision making (Kooiman et al., 2023). Among other data, it included contemporaneous network data from baseline EMA over two weeks.

By design the StayFine intervention-modules included a mix of cognitive intervention-modules (*Psychoeducation, Cognitive restricting)*, behavioral intervention-modules (*Exposure, Activation, Sleep)*, wellbeing intervention-modules that focus on positive mental health aspects (*Wellness* and *Positive Affect*), and a last intervention-module that combines lessons learned into a plan to summarize helpful skills and become aware of potential future triggers for relapse (*StayFine relapse prevention plan)*. Three intervention-modules were offered to all participants (*Psychoeducation, Cognitive restricting,* and *StayFine plan*)*,* and three out of five options were chosen based on personalization (*Exposure, Activation, Sleep, Wellness* and *Positive Affect*).

We aimed to explore whether the network approach as used for personalization in the RCT, can be used to examine effects of personalized combination of intervention-modules as well. As a first exploration, we assessed the relation between the number of completed intervention-modules and change in networks from baseline to post-intervention. Personalization at the individual level resulted in relatively small groups of participants that started the same combination of intervention-modules. Due to study design with baseline and post-intervention assessments, we could not reliably assess the impact of specific intervention-modules on change in networks. Either, at baseline, participants did not yet receive intervention-modules, or they received varying combinations of intervention-modules at post-intervention. Regardless of the combination of intervention-modules (Frederick et al., 2024), we expected that completing a combination of intervention-modules with cognitive, behavioral, and wellbeing underpinnings, would be associated with change in multiple aspects of individual networks simultaneously (Mcnally, 2021; Fried et al., 2017; Bringmann et al., 2022). Given the mixed results in prior studies, and the theoretical concept of ‘shaking up the system’ (a black box idea) with a combination of intervention-modules, we did not have clear hypotheses about which changes would occur in the networks.

We hypothesized that, on average across individuals, more completed intervention-modules would be associated with more change in affect dynamics and network characteristics (e.g. higher mean level of *positive affect*, lower level of *negative affect*, and lower *network density*). A second aim was to examine whether receiving intervention-modules added to care as usual (CAU; regardless of number) resulted in more change in networks as compared to CAU. To this aim, change was compared between randomization groups.

## 2 Methods

In the current study, secondary outcomes from the StayFine RCT (ClinicalTrials.gov: NCT05551468) are reported. The RCT was approved by the Medical Research Ethics Committee Utrecht. All participants (and parents if <16 years) provided written informed consent. The RCT is described in more detail in the published trial protocol (Robberegt et al., 2022). In short, young individuals (13 to 21 years) remitted from depressive or anxiety disorders were randomized (1:1) to personalized varying combinations of intervention-modules added to CAU (M + CAU), or CAU. Randomization was stratified by presence versus absence of prior treatment and number of prior episodes (1, 2, 3 or more). Assessments occurred five times (T0: baseline, T1: post-intervention at 4 months, T2: 1-year follow-up, T3: 2-year follow-up, and T4: 3-year follow-up) and included EMA optionally wearing an accelerometer, self-report questionnaires, and clinical diagnostic interviews. The baseline and post-intervention assessments of EMA were examined in the current study.

### 2.1 Participants

**Fig. 1:**
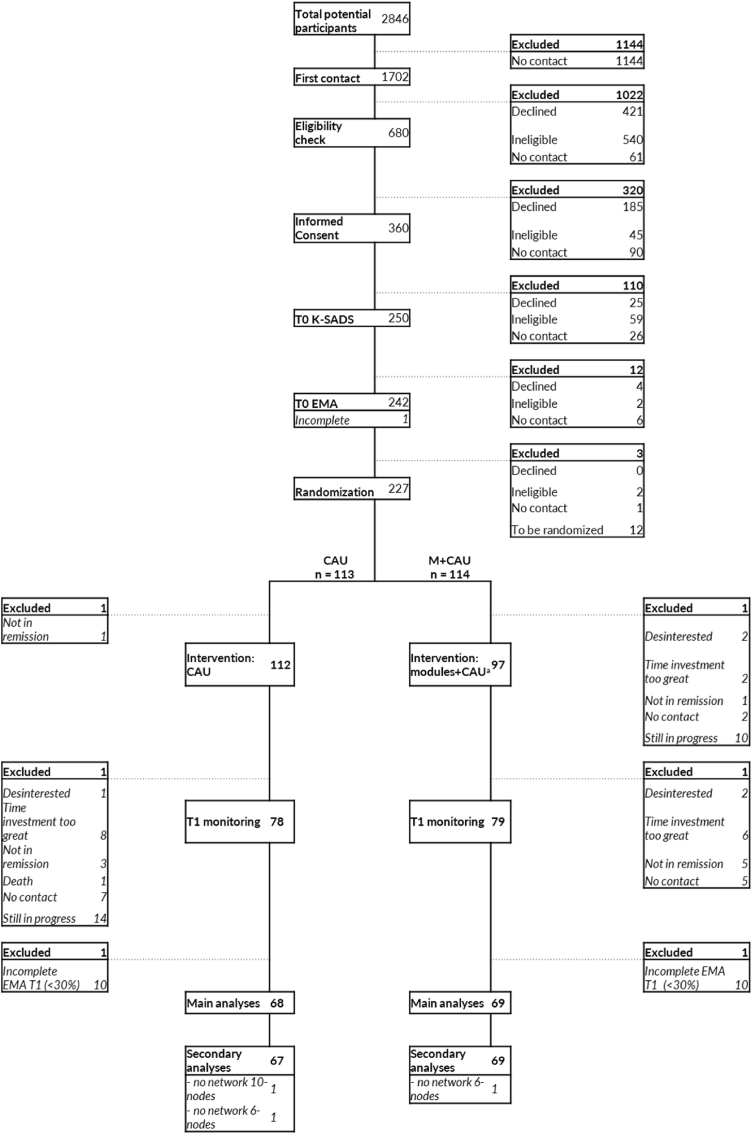
Flowchart. *Note.* CAU = care as usual, EMA = ecological momentary assessment, K-SADS = Kiddie-Schedule for Affective Disorders and Schizophrenia present and lifetime, T0 = baseline assessment, T1 = post-intervention assessment. a = 64 participants of 97 who started the intervention-modules, did not complete all (<6/6) intervention-modules.

Between December 2019 and May 2024, 227 participants were enrolled and randomized in the ongoing study, of which 157 completed baseline and post-intervention EMA at the time of data-extraction, 78 were randomized to M + CAU, and 79 were randomized to CAU. Sixty-nine participants (87%) completed sufficient EMA (≥30%; van der Wal et al., 2023; Wen et al., 2017) at baseline and post-intervention. The sample consisted of 69 participants who received M + CAU (mean age [SD] = 19.52[1.70] years; 60[87%] females, 84% Dutch origin), and 68 participants who received CAU (mean age[SD] = 19.72[1.76] years; 63[93%] females, 88% Dutch origin). In the current sample, compliance with EMA was 85% at T0, and 71% at T1. See Fig. 1 for the participant flow. See Table 1 for sample characteristics.

**Table 1:** Sample characteristics.

Variable	Sample (*N* = 137)				M + CAU (*n* = 69)				CAU (*n* = 68)			
	No.	%			No.	%			No.	%		
Female	123	89.8			60	87			63	93		

	***M***	***SD***	***min***	***max***	***M***	***SD***	***min***	***max***	***M***	***SD***	***min***	***max***

Age	19.61	1.73	14.33	22.42	19.52	1.70	14.92	22.08	19.71	1.75	14.33	22.42

	**No.**	**%**			**No.**	**%**			**No.**	**%**		

Prior episodes												
1	90	65.7			43	62			47	69		
2	33	24.1			19	28			14	21		
3	9	7.3			6	9			3	4		
4	4	2.2			1	1			3	4		
5	1	0			0	0			1	1		
Prior disorders^a^												
Depressive disorders only	26	19.0			17	25			9	13		
Major depressive disorder	25	18.2			17	25			8	12		
Persistent depressive disorder	2	1.5			0	0			2	3		
Anxiety disorders only	13	9.5			7	10			6	9		
Agoraphobia	4	2.9			1	1			3	4		
Generalized anxiety disorder	9	6.6			5	7			4	6		
Panic disorder	6	4.4			3	4			3	4		
Social anxiety disorder	4	2.9			3	4			1	1		
Separation anxiety disorder	3	2.2			1	1			2	3		
Specific phobia	3	2.2			1	1			2	3		
Anxiety and Depressive disorders	96	70.1			45	65			51	75		
Major depressive disorder	93	67.9			44	64			49	72		
Persistent depressive disorder	8	6.6			2	3			6	9		
Agoraphobia	30	19.7			9	13			21	31		
Generalized anxiety disorder	66	46.7			32	46			34	50		
Panic disorder	44	32.8			19	28			25	37		
Separation anxiety disorder	16	10.9			4	6			12	18		
Social anxiety disorder	59	43.1			27	36			32	47		
Specific phobia	29	20.4			13	26			16	24		
Other disorders^b^	81	59.1			38	55			43	63		
One	41	29.9			22	32			19	28		
Two	27	19.7			12	17			15	22		
Three	11	8			3	4			8	12		
Four	2	1.5			1	1			1	1		
Prior psychological treatment	124	90.5			61	88			63	93		
Current other disorder(s)	22	16.1			6	9			16	24		
Current infrequent psychological treatment^c^	17	12.4			9	13			8	12		
Current medication^d^	59	43.1			36	52			23	34		
For depression or anxiety	25	18.2			14	20			11	16		
For other reasons	41	29.9			28	41			13	19		
Dutch nationality birth father	122	89.1			60	87			62	91		
Dutch nationality birth mother	124	90.5			61	88			63	93		
Dutch nationality both parents	118	86.1			58	84			60	88		

	***M***	***SD***	***min***	***max***	***M***	***SD***	***min***	***max***	***M***	***SD***	***min***	***max***

T0 to T1 (weeks)	19.12	5.55	11.00	46.01	20.28	5.75	11.99	43.00	17.95	5.12	11.00	46.01
T0 entries	71.11	10.75	38	84	71.06	10.55	38	84	71.16	11.04	38.00	84.00
T0 entries p/d	5.08	0.77	2.71	6.00	5.08	0.75	2.71	6.00	5.08	0.79	2.71	6.00
T0 entries p/d SD	0.85	0.46	0.00	3.11	0.85	0.41	0.00	1.90	0.85	0.51	0.00	3.11
T1 entries	59.29	15.95	25	83	59.03	15.86	29	83	59.56	16.17	25.00	83.00
T1 entries p/d	4.24	1.14	1.79	5.93	4.22	1.13	2.07	5.93	4.25	1.15	1.79	5.93
T1 entries p/d SD	1.23	0.60	0.27	3.03	1.18	0.54	0.27	2.55	1.28	0.65	0.27	3.03

*Note.* Abbreviations: CAU = care as usual, M = mean, M + CAU = intervention-modules added to care as usual, min = minimum, max = maximum, No. = number, p/d = per day, SD = standard deviation, T0 = baseline assessment, T1 = post-intervention assessment.

a As assessed with the Kiddie-Schedule for Affective Disorders and Schizophrenia present and lifetime (K-SADS-PL DSM-5).

b Disorders could entail eating disorders, obsessive compulsive disorder, posttraumatic stress disorder, and alcohol and drug disorders.

c Infrequent treatment as detailed under heading Participants (e.g. maintenance treatment or counselling).

d Numbers do not add up to, since some have combined medication for depressive or anxiety disorders, and other reasons.

Eligibility for the RCT at study entry was based on the following criteria: age 13 to 21 years during application, and being in remission from major depressive -, persistent depressive -, disruptive mood dysregulation disorder, separation -, social - or generalized anxiety disorder, specific phobia, panic disorder, or agoraphobia. Remission was defined as not meeting the criteria for depression or anxiety for at least two months prior to randomization. For a detailed overview of which depressive and anxiety disorders were (in)eligible, see Supplement 1 in the Supplementary material. Exclusion criteria were prior (hypo)mania, bipolar disorder, prior or current psychotic episode, current alcohol or drug misuse, and more than two monthly treatment sessions. Participants received CAU, which could entail no intervention, pharmacological interventions, or psychological interventions. At enrolment, infrequent treatment aimed at change (not merely support), was allowed no more than twice each month. Examples are low frequency (cognitive) behavioural therapy, eye movement desensitisation and reprocessing (EMDR), psychoeducation, or counselling. Pharmacological interventions for other reasons than depression or anxiety could entail birth control, or medication for concurrent mental (e.g. attention deficit and hyperactivity disorder, autism spectrum disorder) or somatic conditions (e.g. epilepsy). In case someone needed intensified therapy during the study because of relapse or deterioration, this was allowed. Eligibility was assessed using a short telephonic screener and with the Kiddie-Schedule for Affective Disorders and Schizophrenia present and lifetime version (K-SADS-PL DSM-5; Kaufman et al., 2016). The short telephonic screener included a check on inclusion criteria based on age, assigned sex, prior and current episodes and treatment, and the patient health questionnaire (PHQ-4; Khubchandani et al., 2016) to assess depression and anxiety symptoms in the past two weeks. In the current RCT, the interrater reliability for the K-SADS was good to very good: Fleiss Kappa = 0.83 (range 0.65-1).

### 2.2 Measurements

#### 2.2.1 EMA and individual networks

Baseline and post-intervention EMA occurred six times daily, for fourteen consecutive days, typically starting on a Monday. Researchers instructed eligible participants about the two weeks of EMA with the StayFine app (Minddistrict B.V., 2024). Push messages to complete EMA were standardized for each participant, and personalized to be minimally 1 h, and preferably 2 to 3 h, apart between 8.00 and 23:00. Six daily time points were chosen based on a trade-off between the theoretical aim to repeatedly assess dynamic and changeable constructs, and practical feasibility (as obtained from our unpublished pilot study and a systematic review; Wen et al., 2017). Using electronic messaging, researchers provided positive feedback about EMA completion, or active reminders in case of missed entries for >2 days. Post-intervention EMA occurred 4.7 months after baseline EMA. The mean time (SD) between EMA assessments was 20.27 (5.75) weeks, ranging between 12 and 43 weeks.

EMA consisted of sixteen items. The first fourteen were scaled from 0 (totally disagree) to 100 (totally agree): I feel … *anxious, sad, stressed, angry, relaxed, energetic, enthusiastic, cheerful, tired, lonely, I want to suppress my feelings, I avoid difficult things, my current activity was enjoyable,* and *my current activity costed energy*. A binary item was used to indicate social company: being alone (0) or at least with one other person (1). An optional open-ended question was offered to include remarks. Items were based on prior EMA studies (Slofstra et al., 2018), the positive and negative affect scale (Watson et al., 1988), and targets of intervention-modules.

For network construction, the first fifteen EMA items were used, that ranged from 0 (totally disagree) to 100 (totally agree). Two nodes consisted of more than one item: *positive affect* was based on four (relaxed, energetic, enthusiastic, cheerful), and *activity investment* was based on two EMA-items (enjoyable activity, (inverted score of) activity that costed energy). The binary item for social company was rescaled from 0/1 to 0/100. Answers to the open-ended question were left out from statistical analyses. Fatigue was measured with the item ‘tired’. This resulted in a contemporaneous individual partial correlation network, potentially including the following 11 nodes: *anxiety, sadness, positive affect, anger, stress, fatigue, suppression, avoidance, loneliness, activity investment,* and *social company*. Fig. S1 in the Supplementary material shows a baseline and post-intervention network for one participant. Supplement 1 reports EMA and network data preparation steps.

Under the assumption that a combination of intervention-modules shakes up the system of interconnected nodes, we aimed to examine different affect dynamics and network characteristics: *node intensity, node instability, one-step and two-step expected influence centrality* (EI1 & EI2)*,* and *network density*. *Intensity* represents the node's mean level over repeated daily assessments and is the most basic network dynamic (Dejonckheere et al., 2019). *Instability* is the node's fluctuation from assessment to assessment, and was calculated using the root mean square of successive differences (RMSSD ;Jahng et al., 2008; Schoevers et al., 2021). Assessment-to-assessment differences were disregarded when it concerned intervals between days or one or more missing assessments. *Expected influence centrality* consisted of one- and two-step expected influence centrality, being indicative of the relative strength of a node within the network. One-step expected influence (EI1) centrality represents the node's strength by adding the edge-weights of the node to neighbouring nodes in the network (Robinaugh et al., 2016). Two-step expected influence (EI2) centrality additionally accounts for the strength of neighbouring nodes (Robinaugh et al., 2016). *Network density* reflects the overall connectivity of the network (Wichers et al., 2021), and was calculated by dividing the absolute edge weights by the possible number of edges in individual networks.

There could be differences in number and type of nodes between T0 and T1 within persons, because only nodes were included that conformed to the rule of SD >10, corresponding to 10% of the item's range. We deem it is justified to include nodes that fluctuate over time, because these may be the most changeable aspects of mental health. Items that are continuously at the same value, were not depicted in the network, and potentially may be more difficult to affect. To account for items with rather stable values, we extracted the node intensity and instability for each node, regardless of whether it was part of the network. With these metrics, we intended to assess whether the number of intervention-modules could influence rather stable items.

Clinical interpretation of change in these metrics is more straightforward for intensity and instability than for symptom interconnections. In case a decrease in intensity of negative affect occurs, this could be a precursor of lower symptoms. If the assessment-to-assessment stability increases, it could be that affect is more stable over several hours, and potentially less influenced by situations. When adopting the theory of cognitive behavioral therapy, both of these changes could be the result of more neutral or nuanced thoughts about situations. More symptom interconnectedness is more difficult to interpret, due to inclusion of positive and negative nodes in our networks. In case of primarily positive nodes with positive edges, a higher interconnectedness would be indicative of a resilient state. A change to a more interconnected network (i.e. with higher *density*) could therefore be clinically relevant. At the same time, change to a more interconnected network with several negative nodes and highly active edges, could indicate a move away from resilience and being on a tipping point to a more depressed or anxious state. The examples above illustrate how hypothesized changes could be interpreted. At the same time, we note caution because correlations in a contemporaneous network do not infer causality, and prior research reported mixed results about the link between network change and symptom change (e.g. Krijnen-de Bruin et al., 2022; Bruce et al., 2005).

#### 2.2.2 Intervention-modules and personalization

The app-based intervention-modules extended on preventive cognitive therapy (PCT; Bockting, 2009; Legemaat et al., 2023) and cognitive behavioral therapy (CBT; Clarke and Harvey, 2012; Craske et al., 2014; Kennard et al., 2008). The combination of intervention-modules was personalized to include six out of eight possible intervention-modules. Three core intervention-modules were part of each combination (*Psychoeducation, Cognitive restructuring* and *Relapse prevention plan*), and three of five additional intervention-modules were included: *Enhancing positive affect, Behavioral activation, Exposure, Sleep, and Wellness* based on a data-driven advice combined with shared decision making. Data about prior depression and/or anxiety, self-reported sleep, flourishing, and positive and negative affect, and which nodes correlated with *sadness* and *anxiety* nodes in the baseline individual network, were used by researchers to provide an advice (Kooiman et al., 2023). During a telephonic introductory meeting, experts by experience discussed the data-driven advice with the participant. Participants could deviate from the advice based on personal preferences. Common reasons to deviate were: time investment if two behavioral-based intervention-modules (e.g. *Behavioral activation* and *Exposure*) were to be combined, that participants already practiced with techniques in advised intervention-modules (e.g. *Behavioral activation, Exposure,* or *Wellness*), and that some intervention-modules were more appealing than the recommended intervention-modules. Fig. S2 shows eight potential combinations as advised by researchers. Supplement 2 provides an example personalization case.

The eight intervention-modules aimed to: explain about relapse prevention and to schedule times to work on the intervention (*Psychoeducation* (Bockting, 2009); to challenge dysfunctional beliefs, to activate positive thought networks, wishful beliefs, positive affect and schema's (*Cognitive restructuring;* Bockting, 2009); to enhance positive affect through detailed memory training using a positive diary *(Enhancing positive affect;* Bockting, 2009); to practice with behavioral activation (*Behavioral activation*; Kennard et al., 2008); to build positive associations through experience (*Exposure*; Craske et al., 2014, Emslie et al., 2015); to improve sleep behavior and thoughts about sleep (*Sleep*; Clarke and Harvey, 2012, ten Broeke et al., 2008); to engage in social contact, do purposeful, goal-oriented activities, promote a balance between relaxation and activity, and more (*Wellness;* Légaré and Thompson-Leduc, 2014, Ryff and Singer, 1996); and to make a personalized relapse prevention plan including future challenges and ways to handle these (*Relapse prevention plan;* Bockting, 2009, Frederick et al., 2024).

During intervention-modules, participants were guided by expert by experience who provided feedback or answered questions via online messaging (chat-function) in the app. Each intervention-module incorporated psychoeducation and exercises, and most consisted of two parts. After each part, the expert by experience provided positive feedback to comment on exercises and to summarize learned skills. The participant could progress to the next part after reading the feedback. Participants were instructed to invest 1 h per week over the course of approximately three months in order to finish the intervention before post-intervention assessment.

To examine whether deviations from the advice based on shared decision making resulted in different outcomes regarding change in networks, we incorporated sensitivity analyses with a sample of 50 participants (see Supplement 5).

### 2.3 Statistical testing

#### 2.3.1 Data preparation

Missing EMA-data were not imputed. Data preparation steps for EMA data and network construction are detailed in Supplement 1 online. Importantly, networks were constructed using LASSO regularization in *qgraph* (1.9.2; Epskamp et al., 2012). These included maximally 11 nodes with standard deviation (SD) >10, corresponding to 10% of the item's range. Edges represented partial correlations, with an edge-threshold of >0.3 to more reliably depict true edges (Epskamp et al., 2018a; Dobson et al., 2021).

The change in affect dynamics and network characteristics per participant (subtracting the baseline (T0) value from the post-intervention (T1) value) was used as dependent variable in group-level univariate linear regression models and two-sample *t*-tests. The number of intervention-modules was the number of completed intervention-modules at post-intervention (T1) EMA, and was the independent variable in univariate regression models. This variable was rounded to halves, ranging from 0 to 6 completed intervention-modules. The number of completed intervention-modules, regardless of the combination, was considered in the analyses.

#### 2.3.2 Main analyses

Data were prepared and analysed using *Excel* (Microsoft Corporation, 2018) and *R* (version 4.2.2) (R Core Team, 2022). Means (M) and standard deviations (SDs) were calculated for continuous outcomes, and numbers and percentages were reported for binary and ordinal outcomes. In all analyses, *p* < .05 was considered statistically significant, thereby accepting an inflation of type 1 error (Seidl et al., 2021), given the exploratory nature of the analyses. Afterwards, Benjamini-Hochberg correction (Benjamini and Hochberg, 1995) was applied to correct for multiple testing.

As a first step to prepare for regression analyses, boxplots were visually inspected to detect outliers in the change-score of affect dynamics and network characteristics. Potential outliers, indicated by the boxplots, were removed based on group-discussion between the authors who are scientists and scientist-practitioners. Outliers were assessed based on data from clinical interviews about clinical status. If a value was plausible, the observation was maintained. Formal decision rules to exclude outliers were not applied. Next, the normality of residuals assumption was assessed in Q-Q plots of change-scores. Additionally, the homoscedasticity assumption was assessed by plotting residuals of ordinary least squares univariate regression and robust iterated re-weighted least squares (IWLS) regression change-scores in scatterplots, which did not show violation. Robust IWLS univariate regression analyses were used, because these provide more reliable estimates in case of influential observations (which were present for some centrality-variables). In total, 45 exploratory analyses were performed, including one for density and 4 × 11 for 4 affect dynamics and network characteristics (intensity, instability, EI1-, and EI2-centrality) and 11 nodes in the contemporaneous partial correlation networks (*anxious, sad, stressed, angry, positive affect, fatigue, loneliness, suppression, avoidance, activity investment,* and *social company*). The analyses were repeated both including and excluding individual outliers.

#### 2.3.3 Secondary analyses

Three additional analyses were performed: (1) comparison between randomization groups (using two-sample Welch t-tests for each outcome), (2) robustness checks of the regression analyses with alternative networks, (3) a robustness check with a sample that only included participants that chose intervention-modules as advised based on the data. In Supplementary materials, we report on these robustness checks. Differences in pre-processing are reported in each supplement. Supplement 3 presents results from a network without the node *social company,* Supplement 4 of a more parsimonious model of six nodes, and Supplement 5 on sensitivity analyses with a smaller sample, that included only participants that chose intervention-modules as advised based on the data. The analyses were similar to the main analyses.

## 3 Results

### 3.1 Choice and completion of intervention-modules

The current sample consisted of 69 participants randomized to M + CAU, that provided sufficient (≥30%) EMA-data. For included participants (*N* = 69), most often advised intervention-modules were *Wellness* (*n* = 51), *Behavioral activation* (*n* = 49), and *Enhancing positive affect* (*n* = 48). One participant did not complete any intervention-module at the post-intervention assessments, and all others finished 1 to 6 intervention-modules (M[SD] = 4.29[1.70]). Almost four in five participants (*n* = 55; 79.7%) completed at least half of the intervention (≥3 modules), and approximately half of the participants (*n* = 35; 50.7%) started the last module. Table 2 summarises the total number of chosen and completed intervention-modules, also per combination.

**Table 2:** Number of chosen and completed intervention-modules (total and per combination).

Completed number of intervention-modules												
No. int-modules	0	1	1.5	2	2.5	3	3.5	4	4.5	5	5.5	6
(*n* = 69)	1	1	5	6	1	7	4	7	2	7	4	24

		Completed per combination									
Intervention	Completed (total)	Positive affect, activation, sleep (*n* = 8)	Positive affect, activation, wellness (*n* = 17)	Wellness, activation, sleep (*n* = 9)	Positive affect, exposure, sleep (*n* = 5)	Positive affect, exposure, wellness (*n* = 12)	Wellness, exposure, sleep (*n* = 1)	Positive affect, activation, exposure (*n* = 4)	Wellness, activation, exposure (*n* = 10)	Sleep, activation, exposure^a^ (*n* = 1)	Sleep, positive affect, wellness^a^ (*n* = 2)
PE (*n* = 69)	68	8	16	9	5	12	1	4	10	1	2
CR (*n* = 69)	61	7	15^b^	9	3	11	1	3	9	1	2
AC (*n* = 49)	36	5	15	6	-	-	-	2	7	1	-
EX (*n* = 33)	16	-	-	-	1	7	1	2	5	0	-
PA (*n* = 48)	36	6	15	-	2	9	-	3	-	-	1
SL (*n* = 26)	12	5	-	5	1	-	0	-	-	0	1
WN (*n* = 51)	34	-	12	7	-	5	1	-	8	-	1
SF (*n* = 69)	24	5	7	3	1	3	0	2	3	0	0

*Note.* The number of times an intervention-module was chosen is noted on the left, with *n* = x after each intervention-module. Then, the number of completed intervention-modules is reported in total, followed by how many were completed in each combination. The subsample size is noted with *n* = x for each combination of intervention-modules.

Abbreviations: *n* = times chosen/completed, PE = Psychoeducation, CR = cognitive restructuring, AC = Behavioral activation, EX = Exposure, PA = Enhancing positive affect, SL = Sleep, WN = Wellness, SF = StayFine relapse prevention plan.

a Combination was not based on the data-driven advice.

b = 1 participant was never offered part 2 of the module and therefore did not complete the entire module.

### 3.2 Network descriptives

On average, change in network metrics was minimal (between −2% and 4% change), see Table S1 in Supplementary materials. On average, networks consisted of 7 nodes at baseline (M = 6.19; SD = 2.48, range = 0 to 11) and post-intervention (M = 6.26, SD = 2.88, range = 0 to 11), and the change in the number of nodes was 0 from baseline to post-intervention (M = 0.07, SD = 2.66, range = −7 to 6). Fig. S3 in Supplementary material shows a line graph to visualize the change in number of nodes per participant. This graph suggests that changes in number of nodes occur, but that extreme changes are less common.

In the M + CAU group (*n* = 69), most often visualized nodes in the baseline networks of 11 nodes were *positive affect* (*n* = 58), *fatigue* (*n* = 52), and *stress* (*n* = 49). In the post-intervention networks, common nodes were *positive affect* (*n* = 51), *activity* (*n* = 50), and *fatigue* (*n* = 50). Nodes with the highest intensity were *activity, positive affect,* and *fatigue* at baseline and post-intervention. At baseline and post-intervention, node-instability was highest in the nodes *fatigue, activity,* and *stress*. On average, the most-central nodes were *sadness* and *suppression* based on centrality values (bootstrapping methods for centrality ordering were not performed).

In the CAU group, the nodes *positive affect* (*n* = 53), *fatigue* (*n* = 51), and *stress* (*n* = 49) were most common in baseline-networks, and *fatigue* (*n* = 56), *positive affect* (*n* = 52), and *activity* (*n* = 49), in the post-intervention networks. Highest intensity was evident in the nodes *activity, positive affect,* and *fatigue* at baseline and post-intervention. During both assessments, highest instability was reported in the nodes *fatigue, activity,* and *stress*. On average, the most-central nodes (i.e. highest value of expected influence centrality EI1 or EI2-values*)* were *sadness* and *suppression*. Table S1 in Supplementary Materials summarises the network characteristics at baseline, post-intervention, and the change-score, grouped per variable.

### 3.3 Completed modules and change in networks

No association was evident between number of intervention-modules and change in node intensity (11 × ) or instability (11 × ) across individuals.

On average, a higher number of completed intervention-modules was associated with a decrease in node-EI2-centrality of *fatigue* (*n* = 69;*β* = −0.061 95%CI [−0.122,0.000], *p* = .037, *η*_*p*_^*2*^ = 0.03), also after removal of two outliers (*p* = .041). This suggested that *fatigue* would become less influential in the network taking into account the relative strength of neighbouring nodes. However, after correcting for multiple testing, the result was non-significant, which hampers further interpretation. No other change in one- (11 ×), and two step (10 ×) expected influence centrality was significantly associated with the number of completed intervention-modules.

On average across individuals, a higher number of completed intervention-modules was associated with a decrease in *network density* (*n* = 69;*β* = −0.008 95%CI [−0.015,0.000], *p* = .015, *η*_*p*_^*2*^ = 0.05). No outliers were evident for change in density. Again, multiple testing correction resulted in a non-significant association. A post-hoc analysis assessing change in adolescents and young adults, did not show any difference in network density at baseline or in change of density between 13 and 18 (*n* = 27) and 18-22 (*n* = 110) year old individuals.

### 3.4 Secondary analyses

Randomization groups were compared for change in each network characteristic (45 ×). Of 45 *t*-tests for differences between randomization groups (M + CAU versus CAU), one significantly showed a difference: one-step EI-centrality of *loneliness* ((*t[*130.33] = -2.47, *p* = .015, d = −0.42); M_M + CAU_ [SD] = 0.08[0.45]; M_CAU_[SD-] = -0.13[0.53]). Reflecting a mean positive change (increase) in the M + CAU-group, and a mean negative change (decrease) in the CAU-group. However, results were non-significant after multiple testing correction. Given that both randomization groups received CAU, there is limited evidence that intervention-modules have additional effects on change in individual networks.

Additionally, regression analyses with an alternative network (without the node *social company*), with a more parsimonious model of six nodes, and analyses including only participants who chose intervention-modules as advised (*n* = 50) were performed as robustness check. These sensitivity analyses are reported in more detail in the Supplementary material. The results after multiple testing correction were unchanged. To assess whether current null finding are related to an absence in symptom changes, we performed post-hoc analyses on symptom change. These additional analyses suggest that although there is clinical change – a slight increase – over time in depressive and anxiety symptoms, this change is not reflected in network change. Moreover, we examined whether baseline symptom severity was related to the number of completed intervention-modules. This analysis suggested that baseline symptom severity does not positively or negatively correlate with completion of intervention-modules. It is important to note that symptom data were missing for 2 participants in the Modules group and 3 in the No modules group, and that timing of the T1 assessment for the online questionnaires and EMA did not always overlap. The results are reported in Supplement 7 in supplemental material.

## 4 Discussion

In 69 young individuals remitted from depression or anxiety, the association between number of completed intervention-modules added to CAU and change in individual networks from baseline to post-intervention was examined. We expected that more intervention-modules would be associated with more change in networks. Participants completed four intervention-modules on average, and approximately half (*n* = 35) started the sixth (and last) intervention-module before the post-intervention assessment. Although a higher number of completed intervention-modules was associated with change in some network characteristics, findings were not robust after multiple testing correction. Thus, contrary to our expectation, a higher number of completed intervention-modules was not significantly associated with more change in mean level, fluctuation, relative importance of nodes, or overall strength of the network. Furthermore, after multiple testing correction, change in networks was not robustly different between randomization groups (M + CAU [*n* = 69] versus CAU [*n* = 68]). Given the non-robust findings and small effects, we refrain from interpretation of these results. At this point, results on average provide insufficient support that the number of completed intervention-modules is associated with change in individual networks as used for personalization in remitted young individuals.

Current non-robust associations add to the mixed evidence for the clinical application of the network approach (Wichers et al., 2021; Mcnally, 2021). One explanation for current null-findings are floor effects in affect dynamics and network characteristics. Indications for floor effects are (1) the relatively low mean levels for negatively-valanced nodes at baseline (and high mean levels of positively-valanced nodes), (2) that the mean change in individual networks from baseline to post-intervention was minimal, and (3) a relatively low overall network strength at baseline and post-intervention, see Table S1. Hence, we can conclude that the networks were in a rather stable state (Lunansky et al., 2021), instead of on a tipping point. Stable networks are thought to be less affected by external events or activation of symptoms in the network (Scheffer et al., 2024a), and, thus, may be less susceptible to change following a higher number of intervention-modules. It could be that the association between intervention-modules and network characteristics is different during resilient (remitted; Snippe et al., 2017), and depressed or anxious mental states (Schumacher et al., 2023; Beard et al., 2016). If lower overall strength and stability of the network resemble a more stable state of remission, we cannot rule out that change in EMA-based outcomes and networks characteristics will occur over a longer time-span, when relapse is more likely (Paykel, 2008).

Besides floor effects, an alternative explanation for current results is that the number of intervention-modules is not related to change in individual networks. Perhaps, a specific intervention-module (e.g. *Enhancing positive affect*) does result in change in (specific) affect dynamics or network characteristics (e.g. increased mean level of *positive affect*). Even though we did not examine associations for specific intervention-modules, we did examine differential change between randomization groups. Change in networks was not robustly different between groups (M + CAU versus CAU). This partly contrasts finding of (personalized) modular interventions that were superior to standardized protocols or active controls (Graham et al., 2020; Weisz et al., 2012; Vivas-Fernandez et al., 2023; Emslie et al., 2015). However, the interpretation is restricted to change in individual networks in response to combined intervention-modules. Different outcomes could be expected when specific intervention-modules, or other mental health outcomes are examined such as symptomatology and quality of life. Although, in the current sample, randomization did not (yet) have predictive value on change in depressive and anxiety symptoms.

Sensitivity analyses with alternative networks and a smaller sample suggested the results were unchanged after multiple testing correction. The sensitivity analyses suggest that the methodological choices to construct networks influence the reported associations, but not the statistical significance. By adopting a more parsimonious model, we increased statistical power, but did not find significant associations. This suggests that potential floor effects cannot be explained by the number of nodes in the network. In another set of sensitivity analyses with a smaller sample (*n* = 50 versus *n* = 69), different associations between network characteristics and intervention-modules were evident, but not after multiple testing correction. This tentatively suggests that changes in networks across individuals do not depend on adherence to the data-driven advice or deviations based on personal preferences. However, careful interpretation is warranted, due to potential power issues in this smaller sample, and because we did not directly compare both groups.

The results should be interpreted in light of some strengths and limitations. Strengths of our study are that we present unique empirical, and ecological valid data about young individuals remitted from depressive-, anxiety disorders or both. We incorporated personalization based on a multimodal data-driven advice (Kooiman et al., 2023), including baseline individual networks. A second strength is the inclusion of both positively and negatively phrased items into EMA, that were based on intervention targets, such as *positive affect*, and *activity*. This allowed assessment of intervention targets, and not merely depressive or anxiety symptoms (Hofmann et al., 2020; Schumacher et al., 2024).

A first limitation is that the current exploratory analyses were not pre-registered (Schumacher et al., 2024). Secondly, influential observations could not be ruled out due to the relatively small sample size (<200), relatively low number of time points, and due to complexity of the networks. Formal post-hoc power analyses were not performed, but to illustrate, in our sample of 69, with presumed power of 0.8, a medium effect size (0.32) would be necessary to detect change in a network with one edge. Networks typically consisted of seven nodes, and potentially more, meaning there could be 55 edges, which complicates power analyses. For more reliable estimates, we performed robust regression and performed sensitivity analyses including networks with fewer nodes. Moreover, nodes that were continuously at the same value, were not depicted in the network. To account for nodes with rather stable values, we extracted the node intensity and instability for each node. Thirdly, it can be argued that the potential of EMA can be leveraged more by using a multilevel modelling approach. Due to the sample size, amount of time points, and because we wanted to use the same pre-processing steps as used to construct networks for personalization, we deemed a stepped approach justified. As a pragmatic solution to account for temporality of within-person data, we extracted node *instability* at the individual level, which is a commonly used dynamic variable (Dejonckheere et al., 2019). An alternative method would be to construct temporal networks next to contemporaneous networks, to assess temporal associations from assessment-to-assessment. Fourthly, the time interval between baseline and post-intervention ranged from 12 to 43 weeks. A majority of participants had a time interval between 17 and 22 weeks. Although the longer time intervals are not ideal because we average data across participants, and influences of seasonality cannot be ruled out, there are no indications that participants who took longer (>21 weeks), differed from participants who completed the intervention-modules within 12 to 21 weeks, in terms of completed intervention-modules, nor in change in number of nodes. A last limitation is that our sample included more females than males, which influences generalizability. Post-hoc analyses for sex were not performed because the subsample of males was very small (*n* = 9 in the M + CAU group).

Future empirical research using the network approach could be extended and optimized. We suggest examination of intervention-modules and networks over a longer follow-up to decrease possible floor effects in networks due to stable resilient states. It needs to be considered that the potential of EMA and networks additionally depends on the design of the RCT. When EMA is offered during interventions, the effects of specific intervention-modules and the associations with change in individual networks could be more reliably assessed. To better understand how different intervention-modules relate to quantitative network changes, it could be informative to gather qualitative data by consulting participants about experiences with specific intervention-modules. Moreover, it could be interesting to assess whether guidance by experts by experience, who are trained to provide peer support, is different from guidance by clinicians, who are trained to have in depth knowledge of CBT and to change cognitions and behaviours. In addition, to prevent false negative findings, EMA items should be carefully considered. For example, to include theoretically relevant variables (e.g. symptoms as well as psychological processes) with temporal fluctuations (Hofmann et al., 2020). Moreover, to increase statistical power a restricted number of nodes relative to the sample size must be used (Mansueto et al., 2023).Lastly, the use of multimodal sources such as EMA, self-report questionnaires, and clinical interviews, in one study may be useful to not only assess the potential of EMA, but to additionally examine the added value in comparison to more traditional assessments.

## 5 Conclusions

Overall, after multiple-testing correction, more combined intervention-modules were not associated with more change in individual networks. Additionally, change in individual networks was not robustly different between randomization groups. At this point, results do not (yet) provide support for added value of examining the number of intervention-modules in relation to networks as used for personalization. The current examination does provide a starting point to replicate non-robust findings for change in networks in confirmatory analyses. It may be worthwhile to examine how specific intervention-modules are associated with change in networks in remitted young individuals, as well as how methodological choices in EMA or network construction influence associations with intervention-modules. This may result in better mechanistic understanding of change in networks in relation to resilient mental states and intervention-modules. Ultimately, knowing how to handle EMA data for different purposes (e.g. personalization, assessing change in mental states or intervention effects), can contribute to innovative clinical tools for individuals previously confronted with common mental health disorders at a young age.

## Author contribution

Conceptualization: S.R., C.A., Y.S., M.N., C.B.; Data curation: S.R., B.K., N.W.; Formal analysis: S.R., C.B.; Funding acquisition: Y.S., M.N., C.B.; Investigation: S.R., B.K., N.W., A.V., Y.S., M.N., C.B.; Methodology: S.R., C.A., Y.S., M.N., C.B.; Project administration: S.R., B.K., N.W.; Resources: Y.S., C.B.; Software: S.R., C.A., B.K., A.V.; Supervision: C.A., Y.S., M.N., C.B., J.M.; Validation: S.R., B.K., N.W.; Visualization: S.R., C.A., B.K.; Writing—original draft: S.R.; Writing—review and editing: S.R., C.A., B.K., A.V., J.M., N.W., M.N., Y.S., C.B.

## Data-sharing statement

Materials, including an intervention manual, and analysis code for the RCT and current study are not yet available, because this is an ongoing trial for which the main analyses and outcomes are expected in the coming years. Decisions about data-sharing will be made in accordance with international guidelines on data-sharing, the informed consent of participants and any conflict with answering the research questions of the RCT.

## Funding

This work, as part of the project ‘STAY-FINE: a personalized monitoring and intervention app to prevent relapse of anxiety and mood disorders in youth and young adults’ was supported by ZonMw (Grant number: 636310007), GGZ Oost Brabant, Rijksuniversiteit Groningen, Accare, RINO Zuid, and Centre for Urban Mental Health.

## Declaration of competing interest

The authors declare the following financial interests/personal relationships which may be considered as potential competing interests: Dr. Maaike Nauta reports a relationship with Clinical training organizations that includes: speaking and lecture fees and travel reimbursement. Dr. Stikkelbroek is a member of the Dutch multi-disciplinary guideline for depression and Dr. Bockting is a co-developer of this guideline. Dr. Nauta is a member of the national workgroup of the Dutch multi-disciplinary guideline for anxiety problems and disorders. Ms. Robberegt, Mr. Kooiman, drs. Stikkelbroek, Nauta, and Bockting are authors of the StayFine intervention. If there are other authors, they declare that they have no known competing financial interests or personal relationships that could have appeared to influence the work reported in this paper.

## References

[bib1] Batelaan N.M., Bosman R.C., Muntingh A., Scholten W.D., Huijbregts K.M., van Balkom A.J.L.M. (2017). Risk of relapse after antidepressant discontinuation in anxiety disorders, obsessive-compulsive disorder, and post-traumatic stress disorder: systematic review and meta-analysis of relapse prevention trials. BMJ.

[bib2] Beard C., Millner A.J., Forgeard M.J.C., Fried E.I., Hsu K.J., Treadway M.T., Leonard C.V., Kertz S.J., Björgvinsson T. (2016). Network analysis of depression and anxiety symptom relationships in a psychiatric sample. Psychol. Med..

[bib3] Benjamini Y., Hochberg Y. (1995). Controlling the false discovery rate: a practical and powerful approach to multiple testing. J. R. Stat. Soc. Ser. B.

[bib4] Bennett S.D., Shafran R. (2023). Adaptation, personalization and capacity in mental health treatments: a balancing act?. Curr. Opin. Psychiatr..

[bib5] Birmaher B., Arbelaez C., Brent D. (2002). Course and outcome of child and adolescent major depressive disorder. Child Adolesc. Psychiatr. Clin..

[bib6] Bockting C.L.H. (2009).

[bib7] Borsboom D. (2017). A network theory of mental disorders. World Psychiatry.

[bib8] Borsboom D., Cramer A.O.J. (2013). Network analysis: an integrative approach to the structure of psychopathology. Annu. Rev. Clin. Psychol..

[bib9] Bosley H.G., Soyster P.D., Fisher A.J. (2019). Affect dynamics as predictors of symptom severity and treatment response in mood and anxiety disorders: evidence for specificity. J. Person. Res..

[bib10] Breedvelt J.J.F., Warren F.C., Segal Z., Kuyken W., Bockting C.L.H. (2021). Continuation of antidepressants vs sequential psychological interventions to prevent relapse in depression: an individual participant data meta-analysis. JAMA Psychiatry.

[bib11] Breedvelt J.J.F., Brouwer M.E., Harrer M., Semkovska M., Ebert D.D., Cuijpers P., Bockting C.L.H. (2021). Psychological interventions as an alternative and add-on to antidepressant medication to prevent depressive relapse: systematic review and meta-analysis. Br. J. Psychiatry.

[bib12] Breedvelt J.J.F., Karyotaki E., Warren F.C., Brouwer M.E., Jermann F., Hollandare F., Klein N., de Jonge M., Klein D.N., Farb N., Segal Z., Biesheuvel Leliefeld K.E.M., Jarrett R., Vittengl J., Thase M., Ma H., Kuyken W., Shallcross A.J., van Heeringen C., Hoorelbeke K., Koster E., Williams M., Huijbers M.J., Speckens A., Cuijpers P., van Oppen P., Gilbody S., Bockting C.L.H. (2024). An individual participant data meta-analysis of psychological interventions for preventing depression relapse. Nat. Ment. Heal..

[bib13] Bringmann L.F. (2021). Person-specific networks in psychopathology: past, present, and future. Curr. Opin. Psychol..

[bib14] Bringmann L.F., Albers C., Bockting C.L.H., Borsboom D., Ceulemans E., Cramer A., Epskamp S., Eronen M.I., Hamaker E., Kuppens P., Lutz W., McNally R.J., Molenaar P., Tio P., Voelkle M.C., Wichers M. (2022). Psychopathological networks: theory, methods and practice. Behav. Res. Ther..

[bib15] Bruce S.E., Yonkers K.A., Otto M.W., Eisen J.L., Weisberg R.B., Pagano M., Tracie Shea M., Keller M.B. (2005). Influence of psychiatric comorbidity on recovery and recurrence in generalized anxiety disorder, social phobia, and panic disorder: a 12-year prospective study. Am. J. Psychiatry.

[bib16] Clarke G., Harvey A.G. (2012). The complex role of sleep in adolescent depression. Child Adolesc. Psychiatr. Clin. N. Am..

[bib17] Cramer A.O.J., Waldorp L.J., Van Der Maas H.L.J., Borsboom D. (2010). Comorbidity: a network perspective. Behav. Brain Sci..

[bib18] Craske M.G., Treanor M., Conway C.C., Zbozinek T., Vervliet B. (2014). Maximizing exposure therapy: an inhibitory learning approach. Behav. Res. Ther..

[bib19] Curry J., Silva S., Rohde P., Ginsburg G., Kratochvil C., Simons A., Kirchner J., May D., Kennard B.D., Mayes T., Feeny N., Albano A.M., Lavanier S., Reinecke M., Jacobs R., Becker-Weidman E., Weller E., Emslie G., Walkup J., Kastelic E., Burns B., Wells K., March J. (2011). Recovery and recurrence following treatment for adolescent major depression. Arch. Gen. Psychiatry.

[bib20] Deisenhofer A.K., Barkham M., Beierl E.T., Schwartz B., Aafjes-van Doorn K., Beevers C.G., Berwian I.M., Blackwell S.E., Bockting C.L.H., Brakemeier E.L., Brown G., Buckman J.E.J., Castonguay L.G., Cusack C.E., Dalgleish T., de Jong K., Delgadillo J., DeRubeis R.J., Driessen E., Ehrenreich-May J., Fisher A.J., Fried E.I., Fritz J., Furukawa T.A., Gillan C.M., Gómez Penedo J.M., Hitchcock P.F., Hofmann S.G., Hollon S.D., Jacobson N.C., Karlin D.R., Lee C.T., Levinson C.A., Lorenzo-Luaces L., McDanal R., Moggia D., Ng M.Y., Norris L.A., Patel V., Piccirillo M.L., Pilling S., Rubel J.A., Salazar-de-Pablo G., Schleider J.L., Schnurr P.P., Schueller S.M., Siegle G.J., Uher R., Watkins E., Webb C.A., Wiltsey Stirman S., Wynants L., Youn S.J., Zilcha-Mano S., Lutz W., Cohen Z.D. (2024). Implementing precision methods in personalizing psychological therapies: barriers and possible ways forward. Behav. Res. Ther..

[bib21] Dejonckheere E., Mestdagh M., Houben M., Rutten I., Sels L., Kuppens P., Tuerlinckx F. (2019). Complex affect dynamics add limited information to the prediction of psychological well-being. Nat. Hum. Behav..

[bib22] Dobson E.T., Croarkin P.E., Schroeder H.K., Varney S.T., Mossman S.A., Cecil K., Strawn J.R. (2021). Bridging anxiety and depression: a network approach in anxious adolescents. J. Affect. Disord..

[bib23] Emslie G.J., Kennard B.D., Mayes T.L., Nakonezny P.A., Moore J., Jones J.M., Foxwell A.A., King J. (2015). Continued effectiveness of relapse prevention cognitive-behavioral therapy following fluoxetine treatment in youth with major depressive disorder. J. Am. Acad. Child Adolesc. Psychiatry.

[bib24] Epskamp S., Cramer A.O.J., Waldorp L.J., Schmittmann V.D., Borsboom D. (2012). Qgraph: network visualizations of relationships in psychometric data. J. Stat. Software.

[bib25] Epskamp S., Borsboom D., Fried E.I. (2018). Estimating psychological networks and their accuracy: a tutorial paper. Behav. Res. Methods.

[bib26] Epskamp S., van Borkulo C.D., van der Veen D.C., Servaas M.N., Isvoranu A.M., Riese H., Cramer A.O.J. (2018). Personalized network modeling in psychopathology: the importance of contemporaneous and temporal connections. Clin. Psychol. Sci..

[bib27] Fisher A.J., Bosley H.G., Fernandez K.C., Reeves J.W., Soyster P.D., Diamond A.E., Barkin J. (2019). Open trial of a personalized modular treatment for mood and anxiety. Behav. Res. Ther..

[bib28] Frederick J., Yi Ng M., Valente M.J., Venturo-Conerly K., Weisz J.R. (2024). What CBT modules work best for whom? Identifying subgroups of depressed youths by their differential response to specific modules. Behav. Ther..

[bib29] Fried E.I., van Borkulo C.D., Cramer A.O.J., Boschloo L., Schoevers R.A., Borsboom D. (2017). Mental disorders as networks of problems: a review of recent insights. Soc. Psychiatr. Psychiatr. Epidemiol..

[bib30] Gibby B.A., Casline E.P., Ginsburg G.S. (2017). Long-term outcomes of youth treated for an anxiety disorder: a critical review. Clin. Child Fam. Psychol. Rev..

[bib31] Ginsburg G.S., Becker E.M., Keeton C.P., Sakolsky D., Piacentini J., Albano A.M., Compton S.N., Iyengar S., Sullivan K., Caporino N., Peris T., Birmaher B., Rynn M., March J., Kendall P.C. (2014). Naturalistic follow-up of youths treated for pediatric anxiety disorders. JAMA Psychiatry.

[bib32] Graham A.K., Greene C.J., Kwasny M.J., Kaiser S.M., Lieponis P., Powell T., Mohr D.C. (2020). Coached mobile app platform for the treatment of depression and anxiety among primary care patients: a randomized clinical trial. JAMA Psychiatry.

[bib33] Hayes A.M., Yasinski C., Ben Barnes J., Bockting C.L.H. (2015). Network destabilization and transition in depression: new methods for studying the dynamics of therapeutic change. Clin. Psychol. Rev..

[bib34] Hofmann S.G., Curtiss J.E., Hayes S.C. (2020). Beyond linear mediation: toward a dynamic network approach to study treatment processes. Clin. Psychol. Rev..

[bib35] Jahng S., Wood P.K., Trull T.J. (2008). Analysis of affective instability in ecological momentary assessment: indices using successive difference and group comparison via multilevel modeling. Psychol. Methods.

[bib36] Kaufman J., Birmaher B., Axelson D., Perepletchikova F., Brent D., Ryan N. (2016). Comprehensive Psychiatry.

[bib37] Kennard B.D., Stewart S.M., Hughes J.L., Jarrett R.B., Emslie G.J. (2008). Developing cognitive behavioral therapy to prevent depressive relapse in youth. Cognit. Behav. Pract..

[bib38] Khubchandani J., Brey R., Kotecki J., Kleinfelder J., Anderson J. (2016). The psychometric properties of PHQ-4 depression and anxiety screening Scale among college students.

[bib39] Kooiman B.E.A.M., Robberegt S.J., Albers C.J., Bockting C.L.H., Stikkelbroek Y.A.J., Nauta M.H. (2023). Congruency of multimodaldata-driven personalization withshared decision-making forStayFine: Individualizedapp-based relapse prevention foranxiety and depression in youngpeople. Front. Psychiatr..

[bib40] Kovacs M., Obrosky S., George C. (2016). The course of major depressive disorder from childhood to young adulthood: recovery and recurrence in a longitudinal observational study. J. Affect. Disord..

[bib41] Kreiter D., Drukker M., Mujagic Z., Vork L., Rutten B.P.F., van Os J., Masclee A.A.M., Kruimel J.W., Leue C. (2021). Symptom-network dynamics in irritable bowel syndrome with comorbid panic disorder using electronic momentary assessment: a randomized controlled trial of escitalopram vs. placebo. J. Psychosom. Res..

[bib42] Krijnen-de Bruin E., Scholten W.D., Muntingh A., Maarsingh O., van Meijel B., van Straten A., Batelaan N. (2022). Psychological interventions to prevent relapse in anxiety and depression: a systematic review and meta-analysis. PLoS One.

[bib43] Kuyken W., Warren F.C., Taylor R.S., Whalley B., Crane C., Bondolfi G., Hayes R., Huijbers M., Ma H., Schweizer S., Segal Z., Speckens A., Teasdale J.D., Van Heeringen K., Williams M., Byford S., Byng R., Dalgleish T. (2016). Efficacy of mindfulness-based cognitive therapy in prevention of depressive relapse an individual patient data meta-analysis from randomized trials. JAMA Psychiatry.

[bib44] Légaré F., Thompson-Leduc P. (2014). Twelve myths about shared decision making. Patient Educ. Counsel..

[bib45] Legemaat A.M., Burger H., Geurtsen G.J., Brouwer M., Spinhoven P., Denys D., Bockting C.L.H. (2023). Effects up to 20-year follow-up of preventive cognitive therapy in adults remitted from recurrent depression: the DELTA study. Psychother. Psychosom..

[bib46] Levy H.C., Stevens K.T., Tolin D.F. (2021). Research review: a meta‐analysis of relapse rates in cognitive behavioral therapy for anxiety and related disorders in youth. JCPP (J. Child Psychol. Psychiatry).

[bib47] Lunansky G., van Borkulo C.D., Haslbeck J.M.B., van der Linden M.A., Garay C.J., Etchevers M.J., Borsboom D. (2021). The mental health ecosystem: extending symptom networks with risk and protective factors. Front. Psychiatr..

[bib48] Mansueto A.C., Wiers R.W., van Weert J.C.M., Schouten B.C., Epskamp S. (2023). Investigating the feasibility of idiographic network models. Psychol. Methods.

[bib49] Mcnally R.J. (2021). Network analysis of psychopathology: controversies and challenges. Annu. Rev. Clin. Psychol..

[bib50] (2018). Microsoft Corporation, Microsoft Excel.

[bib51] Moffitt T.E., Caspi A., Taylor A., Kokaua J., Milne B.J., Polanczyk G., Poulton R. (2010). How common are common mental disorders? Evidence that lifetime prevalence rates are doubled by prospective versus retrospective ascertainment. Psychol. Med..

[bib52] Nye A., Delgadillo J., Barkham M. (2023). Efficacy of personalized psychological interventions: a systematic review and meta-analysis. J. Consult. Clin. Psychol..

[bib53] Paykel E.S. (2008). Partial remission, residual symptoms, and relapse in depression. Dialogues Clin. Neurosci..

[bib54] (2022). R Core Team, Rstudio.

[bib55] Robberegt S.J., Kooiman B.E.A.M., Albers C.J., Nauta M.H., Bockting C.L.H., Stikkelbroek Y.A.J. (2022). Personalised app-based relapse prevention of depressive and anxiety disorders in remitted adolescents and young adults: a protocol of the StayFine RCT. BMJ Open.

[bib56] Robberegt S.J., Brouwer M.E., Kooiman B.E.A.M., Stikkelbroek Y.A.J., Nauta M.H., Bockting C.L.H. (2023). Meta-analysis: relapse prevention strategies for depression and anxiety in remitted adolescents and young adults. J. Am. Acad. Child Adolesc. Psychiatry.

[bib57] Robinaugh D.J., Millner A.J., McNally R.J. (2016). Identifying highly influential nodes in the complicated grief network. J. Abnorm. Psychol..

[bib58] Robinaugh D.J., Hoekstra R.H.A., Toner E.R., Borsboom D. (2020). The network approach to psychopathology: a review of the literature 2008-2018 and an agenda for future research. Psychol. Med..

[bib59] Ryff C.D., Singer B. (1996). Psychological well-being: meaning, measurement, and implications for psychotherapy research. Psychother. Psychosom..

[bib60] Sanford B.T., Ciarrochi J., Hofmann S.G., Chin F., Gates K.M., Hayes S.C. (2022). Toward empirical process-based case conceptualization: an idionomic network examination of the process-based assessment tool. J. Context. Behav. Sci..

[bib61] Scheffer M., Bockting C.L.H., Borsboom D., Cools R., Delecroix C., Hartmann J.A., Kendler K.S., van de Leemput I., van der Maas H.L.J., van Nes E., Mattson M., McGorry P.D., Nelson B. (2024). A dynamical systems view of psychiatric disorders — theory: a review. JAMA Psychiatry.

[bib62] Scheffer M., Bockting C.L.H., Borsboom D., Cools R., Delecroix C., Hartmann J.A., Kendler K.S., van de Leemput I., van der Maas H.L.J., van Nes E., Mattson M., McGorry P.D., Nelson B. (2024). A dynamical systems view of psychiatric disorders — practical implications: a review. JAMA Psychiatry.

[bib63] Schoevers R.A., Van Borkulo C.D., Lamers F., Servaas M.N., Bastiaansen J.A., Beekman A.T.F., Van Hemert A.M., Smit J.H., Penninx B.W.J.H., Riese H. (2021). Affect fluctuations examined with ecological momentary assessment in patients with current or remitted depression and anxiety disorders. Psychol. Med..

[bib64] Schumacher L., Klein J.P., Elsaesser M., Härter M., Hautzinger M., Schramm E., Kriston L. (2023). Implications of the network theory for the treatment of mental disorders: a secondary analysis of a randomized clinical trial. JAMA Psychiatry.

[bib65] Schumacher L., Burger J., Echterhoff J., Kriston L. (2024). Methodological and statistical practices of using symptom networks to evaluate mental health interventions: a review and reflections. Multivariate Behav. Res..

[bib66] Schweren L., Van Borkulo C.D., Fried E., Goodyer I.M. (2018). Assessment of symptom network density as a prognostic marker of treatment response in adolescent depression. JAMA Psychiatry.

[bib67] Seidl E., Venz J., Ollmann T.M., Voss C., Hoyer J., Pieper L., Beesdo-Baum K. (2021). How current and past anxiety disorders affect daily life in adolescents and young adults from the general population—An epidemiological study with ecological momentary assessment, depress. Anxiety.

[bib68] Slofstra C., Nauta M.H., Bringmann L.F., Klein N.S., Albers C.J., Batalas N., Wichers M., Bockting C.L.H. (2018). Individual negative affective trajectories can be detected during different depressive relapse prevention strategies. Psychother. Psychosom..

[bib69] Snippe E., Viechtbauer W., Geschwind N., Klippel A., De Jonge P., Wichers M. (2017). The impact of treatments for depression on the dynamic network structure of mental states: two randomized controlled trials. Sci. Rep..

[bib70] Snippe E., Smit A.C., Kuppens P., Burger H., Ceulemans E. (2023). Recurrence of depression can be foreseen by monitoring mental states with statistical process control. J. Psychopathol. Clin. Sci..

[bib71] Strauss G.P., Esfahlani F.Z., Sayama H., Kirkpatrick B., Opler M.G., Saoud J.B., Davidson M., Luthringer R. (2020). Network analysis indicates that avolition is the most central domain for the successful treatment of negative symptoms: evidence from the roluperidone randomized clinical trial. Schizophr. Bull..

[bib72] ten Broeke E., van der Heiden C., Meijer S., Hamelink H. (2008).

[bib73] van Borkulo C., Boschloo L., Borsboom D., Penninx B.W.J.H., Lourens J.W., Schoevers R.A. (2015). Association of symptom network structure with the course of longitudinal depression. JAMA Psychiatry.

[bib74] van De Leemput I.A., Wichers M., Cramer A.O.J., Borsboom D., Tuerlinckx F., Kuppens P., van Nes E.H., Viechtbauer W., Giltay E.J., Aggen S.H., Derom C., Jacobs N., Kendler K.S., van Der Maas H.L.J., Neale M.C., Peeters F., Thiery E., Zachar P., Scheffer M. (2014). Critical slowing down as early warning for the onset and termination of depression. Proc. Natl. Acad. Sci. U. S. A.

[bib75] van der Wal J.M., van Borkulo C.D., Haslbeck J.M.B., Slofstra C., Klein N.S., Blanken T.F., Deserno M.K., Lok A., Nauta M.H., Bockting C.L.H. (2023). Differential impact of preventive cognitive therapy while tapering antidepressants versus maintenance antidepressant treatment on affect fluctuations and individual affect networks and impact on relapse: a secondary analysis of a randomised controlled trial. EClinicalMedicine.

[bib76] Vivas-Fernandez M., Garcia-Lopez L.J., Piqueras J.A., Espinosa-Fernandez L., Muela-Martinez J.A., Jimenez-Vazquez D., Diaz-Castela M. del M., Ehrenreich-May J. (2023). A 12-month follow-up of PROCARE+, a transdiagnostic, selective, preventive intervention for adolescents at-risk for emotional disorders. Child Psychiatr. Hum. Dev..

[bib77] Watson D., Clark L.A., Tellegen A. (1988). Development and validation of brief measures of positive and negative affect: the PANAS scales. J. Personality Soc. Psychol..

[bib78] Weisz J.R., Chorpita B.F., Palinkas L.A., Schoenwald S.K., Miranda J., Bearman S.K., Daleiden E.L., Ugueto A.M., Ho A., Martin J., Gray J., Alleyne A., Langer D.A., Southam-Gerow M.A., Gibbons R.D., Glisson C., Green E.P., Hoagwood K.E., Kelleher K., Landsverk J., Mayberg S. (2012). Testing standard and modular designs for psychotherapy treating depression, anxiety, and conduct problems in youth: a randomized effectiveness trial. Arch. Gen. Psychiatry.

[bib79] Wen C.K.F., Schneider S., Stone A.A., Spruijt-Metz D. (2017). Compliance with mobile ecological momentary assessment protocols in children and adolescents: a systematic review and meta-analysis. J. Med. Internet Res..

[bib80] White K.S., Payne L.A., Gorman J.M., Shear M.K., Woods S.W., Saksa J.R., Barlow D.H. (2013). Does maintenance CBT contribute to long-term treatment response of panic disorder with or without agoraphobia? A randomized controlled clinical trial. J. Consult. Clin. Psychol..

[bib81] Wichers M., Riese H., Hodges T.M., Snippe E., Bos F.M. (2021). A narrative review of network studies in depression: what different methodological approaches tell us about depression. Front. Psychiatr..

