# Supplementary material for: Examining varying number of intervention-modules and change in individual networks in young people remitted from depression or anxiety: Exploratory outcomes of the StayFine randomized clinical trial
Source: Neurosci Appl. 2026 Apr 30;5:107007. doi: 10.1016/j.nsa.2026.107007 (PMC13195766; doi:10.1016/j.nsa.2026.107007)
Supplement: Multimedia component 1 [file mmc1.docx]

# Supplementary Material to “Examining Varying Number of Intervention-Modules and Change in Network Dynamics in Young People Remitted from Depression or Anxiety: Exploratory Outcomes of the StayFine Randomized Clinical Trial*”* in Neuroscience Applied

**Authors:** S.J. Robberegt, C.J. Albers, B.E.A.M. Kooiman, A.H. Vuuregge, J.D. Mul, N. Wilts, M.H. Nauta, Y.A.J. Stikkelbroek, C.L.H. Bockting

## Abbreviations

BDI = Beck Depression Inventory

CAU = Care as usual

EMA = Ecological momentary assessment

K-SADS-PL DSM-5 = Kiddie-Schedule for Affective Disorders and Schizophrenia present and lifetime version

LASSO = Least Absolute Shrinkage and Selection Operator regularization

M+CAU = Intervention-modules added to care as usual

RCADS = Revised Child Anxiety and Depression Scale

RCT = Randomized clinical trial

**Fig. S1**

*Exemplar networks at T0 and T1 of participant 1726 using maximally 11 nodes*

| **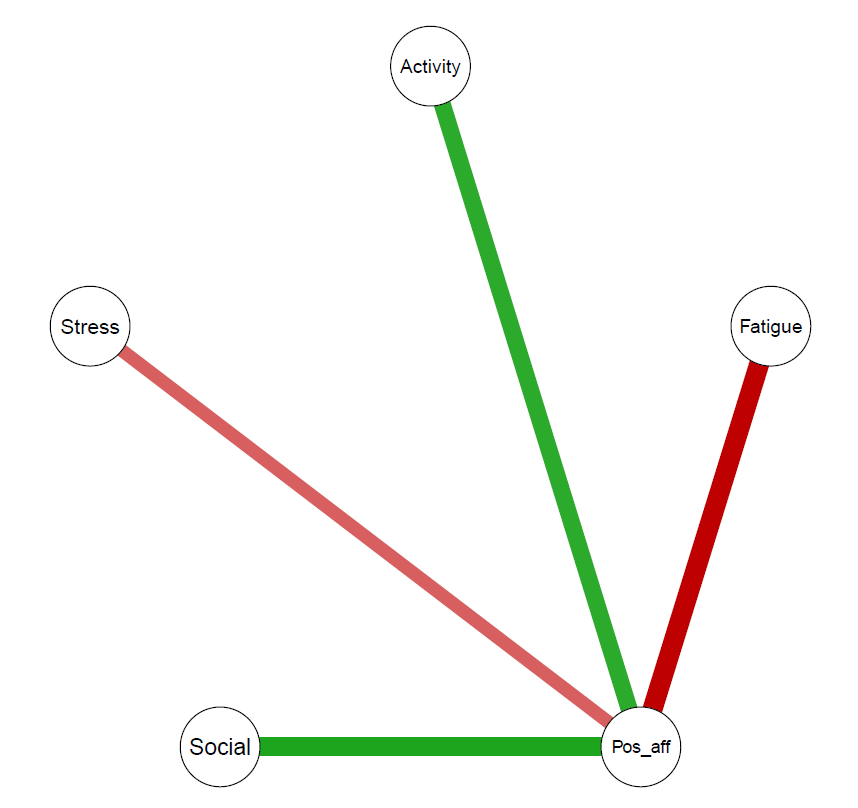** |
| --- |
| **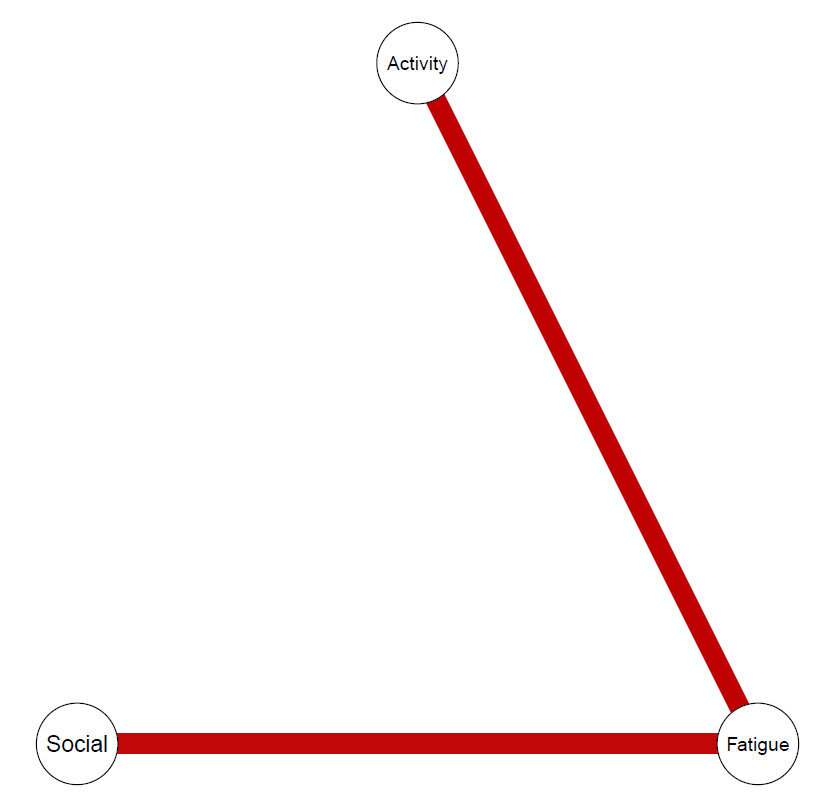** |

*Note.* Depicted nodes are Activity (which is an aggregate of the items ‘my current activity was enjoyable, my current activity costed energy’, Fatigue (I feel… tired), Positive affect (Pos_aff; which is an aggregate of the items ‘I feel… relaxed, energetic, enthusiastic, cheerful’), Social company (Social; which is a binary item of social company), and Stress (I feel… stressed). Red lines depict a negative partial correlation, green lines depict a positive partial correlation. Thicker lines resemble stronger partial correlations.

## Supplement 1 Methods

### Inclusion and exclusion criteria for prior depressive and anxiety disorders

Eligible depressive and anxiety disorders: Major depressive -, persistent depressive -, disruptive mood dysregulation disorder, and separation -, social – or generalized anxiety disorder, specific phobia, panic disorder, agoraphobia.

Ineligible depressive and anxiety disorders: Premenstrual dysphoric disorder, depressive disorder due to another medical condition, substance/medication-induced depressive disorder, other specified depressive disorder, unspecified depressive disorder, and selective mutism, substance/medication-induced anxiety disorder or anxiety disorder due to another medical condition, other specified anxiety disorder, unspecified anxiety disorder.

### Data preparation ecological momentary assessment and networks

The data preparation and analyses made use of the *R* packages (and respective versions) *dplyr* (1.0.10; [1]), *lubridate* (1.9.0; [2])*, MASS* (7.3.6; [3]), *qgraph* (1.9.2; [4])*,* *readxl* (1.4.1; [5]), *repmod* (0.1.7; [6]), *reshape2* (1.4.4; [7]), *rstatix* (0.7.2; [8]), *tidyverse* (1.3.2; [9]), *varhandle* (2.0.5; [10]), and *varian* (0.2.2; [11]).

Ecological momentary assessment (EMA) data were selected as follows: first, the fourteen days of EMA corresponding to the baseline and post-intervention assessment were determined. Then, by semi-automatically looping through the entries, entries between 6:00 and 23:59 within one hour were removed to include the last, while simultaneously adding missing values between 6:00 and 23:59 on days with fewer than six entries. The missing entry was automatically added as an empty entry between two entries which showed the largest time difference between entries. Afterwards, entries before 6 AM were considered an entry of the previous day and manually assigned to that day. When any of these data-cleaning steps resulted in an error in the automated procedure due to an excess of entries (>6 entries), the entry with the smallest time difference was manually removed. In total, the dataset per participant consisted of maximally 84 entries and 14 empty entries between days (i.e. for every night), resulting in 98 entries. In line with prior studies [12,13], participants were included when compliance rate with EMA was above 30%. Missing EMA-data were not imputed.

Contemporaneous individual partial correlation networks were constructed using LASSO regularization in *qgraph* (1.9.2 [4]). These included maximally 11 nodes with standard deviation (SD) >10, corresponding to 10% of the item’s range. Edges represented partial correlations, with an edge-threshold of >0.3 to more reliably depict true edges [14,15]. Extracted affect dynamics and network characteristics at baseline and post-intervention were node intensity, instability, one-step and two-step expected influence centrality, and the overall network density. For transparency, the r-code regarding network construction is included in Supplement 6.

**Fig. S2**

*Advised combinations of intervention-modules*

**
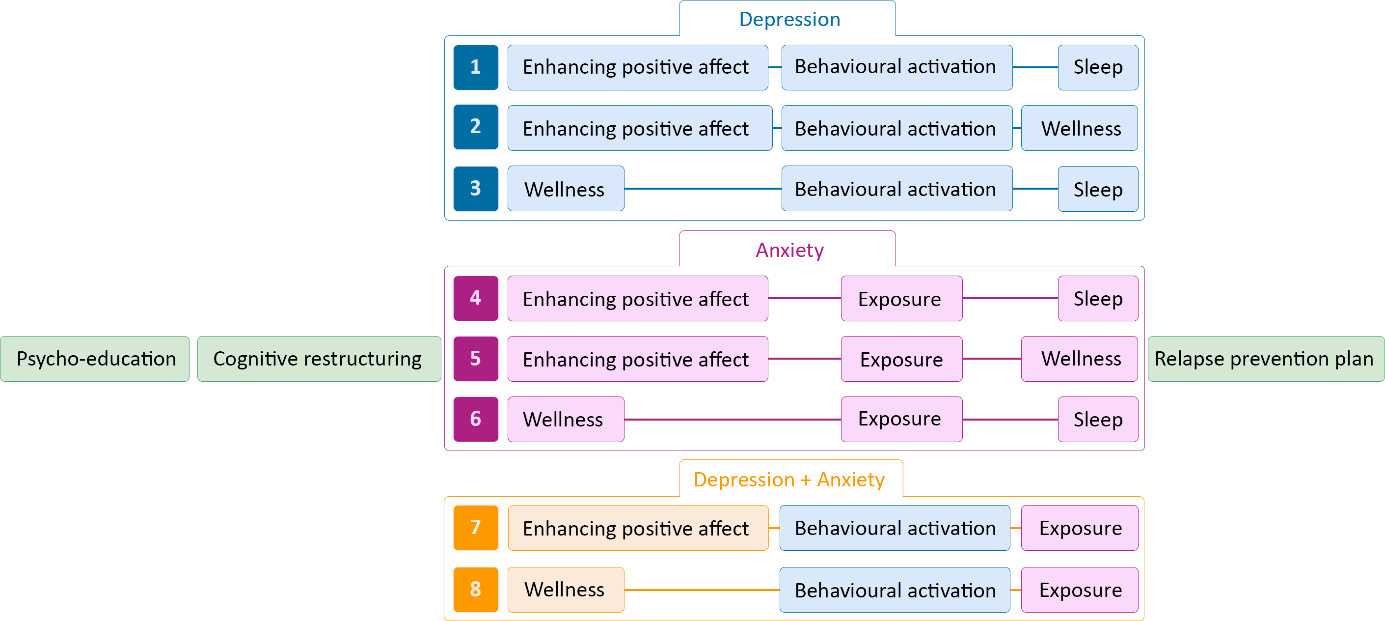
**

Note. *Behavioral activation* or *Exposure* were always part of the advice owing to the prior episode(s). Enhancing positive affect, sleep, and wellness could be advised resulting from scores on self-report questionnaires or connections with *sadness* or *anxiety* nodes in individual networks based on ecological momentary assessment.

## Supplement 2 Example case

Kooiman and colleagues [16] provided support that multimodal data (clinical interview, self-report questionnaires, and associations in an individual network) can provide complementary advice for personalization of modular relapse prevention intervention-modules, next to shared decision making. Here we present data from the baseline assessment (T0) of an exemplar participant, to illustrate the personalization procedure. The clinical interview of the exemplar participant indicated prior depressive episodes. The intervention-module *Behavioral activation* was advised, because it can contribute to improved *activity* and lower *sadness*. Since the participant did not report prior anxiety episodes, the advice was narrowed to combinations 1, 2, or 3 in Figure S2 above. Thereafter, self-report questionnaires indicated lower positive affect and average negative affect, suggesting *Enhancing positive affect* could help. The wellbeing and sleep questionnaires did not indicate a specific intervention-aim. This left combination 1 or 2 as most fitting combination. Then, the baseline network based on EMA indicated that the node *sadness* was positively associated with *loneliness*. This suggested that at times the participant felt sad, she often felt lonely as well. Her network indicated that interventions aiming to lower *loneliness* would be advised. This could include *Cognitive restructuring* to improve functional attitudes about the individual and others (e.g. ‘*I am likeable’* or ‘*others are kind and interested’*), and *Wellness* to improve social skills to counteract thoughts and behaviors related to *loneliness*. Based on the data, combination 2 in Figure S2 was advised as first choice, because it includes the intervention-modules *Psychoeducation, Cognitive restructuring*, *Enhancing positive affect* (self-report questionnaires), *Behavioral activation* (clinical interview), *Wellness* (network), and *Relapse prevention plan.* The participant could choose any combination based on shared decision making, and decided to start with the advised intervention-modules.

**Table S1**

*Affect dynamics and network characteristics at baseline, post-intervention, and change-score*

| **Characteristic** | **M+CAU (*n*  = 69)** | | | | | **CAU (*n* = 68)** | | | | |
| --- | --- | --- | --- | --- | --- | --- | --- | --- | --- | --- |
|  |  | ***M*** | ***SD*** | ***min*** | ***max*** |  | ***M*** | ***SD*** | ***min*** | ***max*** |
| T0 density |  | 0.08 | 0.05 | 0.00 | 0.23 |  | 0.08 | 0.05 | 0.00 | 0.27 |
| T1 density |  | 0.08 | 0.04 | 0.00 | 0.29 |  | 0.09 | 0.05 | 0.00 | 0.26 |
| Δ density |  | 0.00 | 0.06 | -0.20 | 0.17 |  | 0.01 | 0.07 | -0.20 | 0.20 |
| T0 intensity sadness |  | 14.46 | 11.69 | 0.00 | 44.71 |  | 15.10 | 13.05 | 0.09 | 52.56 |
| T1 intensity sadness |  | 16.67 | 15.10 | 0.00 | 69.01 |  | 15.68 | 13.99 | 0.06 | 57.46 |
| Δ intensity sadness |  | 2.22 | 10.64 | -19.41 | 44.21 |  | 0.57 | 8.42 | -15.17 | 27.93 |
| T0 intensity anxiety |  | 11.44 | 11.11 | 0.00 | 55.85 |  | 10.91 | 11.42 | 0.00 | 39.72 |
| T1 intensity anxiety |  | 12.99 | 14.57 | 0.00 | 66.10 |  | 10.83 | 11.46 | 0.00 | 46.24 |
| Δ intensity anxiety |  | 1.55 | 7.86 | -10.64 | 27.49 |  | -0.08 | 7.10 | -21.72 | 21.62 |
| T0 intensity stress |  | 26.32 | 16.66 | 0.38 | 68.96 |  | 26.96 | 17.53 | 1.17 | 72.14 |
| T1 intensity stress |  | 27.74 | 18.07 | 0.07 | 89.97 |  | 25.51 | 16.48 | 0.01 | 66.68 |
| Δ intensity stress |  | 1.42 | 14.16 | -45.68 | 36.08 |  | -1.45 | 10.45 | -22.09 | 25.71 |
| T0 intensity anger |  | 7.68 | 8.38 | 0.00 | 45.69 |  | 9.30 | 8.41 | 0.00 | 32.96 |
| T1 intensity anger |  | 8.61 | 10.00 | 0.00 | 47.61 |  | 9.77 | 10.07 | 0.00 | 43.80 |
| Δ intensity anger |  | 0.94 | 5.29 | -14.36 | 20.66 |  | 0.47 | 7.78 | -20.42 | 39.68 |
| T0 intensity positive affect |  | 56.96 | 12.34 | 17.52 | 85.75 |  | 58.40 | 14.84 | 7.55 | 92.27 |
| T1 intensity positive affect |  | 56.46 | 14.51 | 9.01 | 87.42 |  | 57.63 | 16.03 | 7.44 | 94.39 |
| Δ intensity positive affect |  | -0.50 | 9.21 | -34.66 | 13.79 |  | -0.77 | 8.96 | -28.44 | 25.68 |
| T0 intensity fatigue |  | 49.73 | 16.86 | 5.93 | 86.33 |  | 44.54 | 14.84 | 13.00 | 83.46 |
| T1 intensity fatigue |  | 48.97 | 16.13 | 13.89 | 96.72 |  | 43.89 | 16.98 | 11.08 | 85.86 |
| Δ intensity fatigue |  | -0.77 | 10.96 | -19.28 | 30.97 |  | -0.65 | 11.91 | -44.26 | 25.73 |
| T0 intensity loneliness |  | 15.73 | 14.63 | 0.00 | 57.74 |  | 15.51 | 15.53 | 0.00 | 68.66 |
| T1 intensity loneliness |  | 16.65 | 16.74 | 0.00 | 66.63 |  | 16.28 | 16.63 | 0.00 | 61.08 |
| Δ intensity loneliness |  | 0.93 | 9.81 | -23.18 | 30.47 |  | 0.77 | 10.76 | -26.50 | 52.36 |
| T0 intensity suppression |  | 15.09 | 14.19 | 0.00 | 55.82 |  | 15.10 | 15.13 | 0.00 | 65.67 |
| T1 intensity suppression |  | 18.59 | 19.10 | 0.00 | 93.94 |  | 15.97 | 14.86 | 0.00 | 59.47 |
| Δ intensity suppression |  | 3.50 | 12.38 | -24.32 | 49.13 |  | 0.88 | 12.31 | -25.44 | 47.76 |
| T0 intensity social contact |  | 50.68 | 18.87 | 0.00 | 83.82 |  | 54.93 | 19.73 | 0.00 | 98.72 |
| T1 intensity social contact |  | 51.56 | 18.26 | 0.00 | 85.71 |  | 57.70 | 17.19 | 5.56 | 98.73 |
| Δ intensity social contact |  | 0.88 | 14.33 | -30.39 | 46.57 |  | 2.76 | 18.26 | -36.66 | 77.93 |
| T0 intensity activity investment |  | 61.95 | 8.48 | 45.54 | 83.83 |  | 64.53 | 10.08 | 41.47 | 95.81 |
| **Characteristic** | **M+CAU (*n* = 69)** | | | | | **CAU (*n* = 68)** | | | | |
|  |  | ***M*** | ***SD*** | ***min*** | ***max*** |  | ***M*** | ***SD*** | ***min*** | ***max*** |
| T1 intensity activity investment |  | 61.15 | 9.58 | 40.66 | 90.80 |  | 64.20 | 11.51 | 39.26 | 96.89 |
| Δ intensity activity investment |  | -0.80 | 6.42 | -21.14 | 17.36 |  | -0.34 | 6.99 | -15.78 | 19.15 |
| T0 intensity avoidance |  | 13.64 | 13.15 | 0.00 | 44.30 |  | 13.96 | 14.54 | 0.00 | 56.62 |
| T1 intensity avoidance |  | 16.55 | 16.57 | 0.00 | 74.42 |  | 15.28 | 14.36 | 0.00 | 60.49 |
| Δ intensity avoidance |  | 2.91 | 11.10 | -29.30 | 36.13 |  | 1.32 | 12.95 | -47.40 | 48.90 |
| T0 instability sadness |  | 15.95 | 9.48 | 0.00 | 34.97 |  | 15.79 | 8.78 | 0.76 | 35.55 |
| T1 instability sadness |  | 14.60 | 9.85 | 0.00 | 43.75 |  | 15.62 | 9.64 | 0.66 | 35.25 |
| Δ instability sadness |  | -1.35 | 10.07 | -30.39 | 43.53 |  | -0.17 | 6.97 | -17.06 | 16.42 |
| T0 instability anxiety |  | 13.40 | 9.77 | 0.00 | 58.36 |  | 12.16 | 10.06 | 0.00 | 37.44 |
| T1 instability anxiety |  | 11.54 | 9.18 | 0.00 | 34.50 |  | 10.95 | 9.57 | 0.00 | 44.76 |
| Δ instability anxiety |  | -1.85 | 7.84 | -23.87 | 20.88 |  | -1.21 | 6.74 | -17.84 | 21.43 |
| T0 instability stress |  | 21.27 | 8.40 | 1.91 | 44.11 |  | 21.24 | 8.42 | 1.54 | 40.03 |
| T1 instability stress |  | 20.83 | 8.26 | 0.98 | 40.56 |  | 20.56 | 9.58 | 0.12 | 37.45 |
| Δ instability stress |  | -0.44 | 7.23 | -17.89 | 19.80 |  | -0.68 | 7.57 | -21.41 | 17.95 |
| T0 instability anger |  | 12.57 | 9.11 | 0.00 | 41.89 |  | 13.15 | 8.75 | 0.00 | 39.63 |
| T1 instability anger |  | 11.55 | 8.50 | 0.00 | 47.03 |  | 11.85 | 7.98 | 0.00 | 32.78 |
| Δ instability anger |  | -1.02 | 9.32 | -25.72 | 47.03 |  | -1.30 | 7.44 | -23.89 | 16.51 |
| T0 instability positive affect |  | 15.70 | 5.30 | 4.69 | 34.92 |  | 15.77 | 6.07 | 4.50 | 31.44 |
| T1 instability positive affect |  | 14.59 | 4.82 | 6.36 | 29.97 |  | 15.09 | 6.44 | 3.34 | 33.63 |
| Δ instability positive affect |  | -1.11 | 4.11 | -13.57 | 11.06 |  | -0.68 | 4.16 | -11.68 | 9.39 |
| T0 instability fatigue |  | 26.47 | 7.79 | 9.25 | 52.01 |  | 27.13 | 8.66 | 8.57 | 47.96 |
| T1 instability fatigue |  | 26.34 | 8.54 | 6.86 | 44.49 |  | 25.51 | 9.33 | 8.93 | 49.19 |
| Δ instability fatigue |  | -0.12 | 7.04 | -21.01 | 17.60 |  | -1.62 | 7.19 | -18.04 | 15.06 |
| T0 instability loneliness |  | 16.39 | 10.53 | 0.00 | 35.03 |  | 15.84 | 9.55 | 0.00 | 37.35 |
| T1 instability loneliness |  | 15.40 | 11.71 | 0.00 | 49.44 |  | 14.83 | 10.36 | 0.00 | 38.11 |
| Δ instability loneliness |  | -0.99 | 10.42 | -32.86 | 38.23 |  | -1.01 | 9.02 | -22.87 | 21.13 |
| T0 instability suppression |  | 16.52 | 9.68 | 0.00 | 47.21 |  | 16.00 | 11.26 | 0.00 | 43.65 |
| T1 instability suppression |  | 16.44 | 11.34 | 0.00 | 51.20 |  | 15.13 | 10.68 | 0.00 | 46.35 |
| Δ instability suppression |  | -0.08 | 9.87 | -22.55 | 41.96 |  | -0.87 | 9.20 | -23.13 | 20.92 |
| T0 instability social contact |  | 55.57 | 11.39 | 0.00 | 74.05 |  | 56.12 | 12.96 | 0.00 | 84.85 |
| T1 instability social contact |  | 55.44 | 12.96 | 0.00 | 79.77 |  | 55.45 | 11.47 | 0.00 | 76.38 |
| Δ instability social contact |  | -0.13 | 11.24 | -26.09 | 31.01 |  | -0.67 | 14.43 | -46.55 | 46.67 |
| T0 instability activity investment |  | 21.74 | 7.29 | 5.67 | 47.25 |  | 22.05 | 6.17 | 9.28 | 40.84 |
| T1 instability activity investment |  | 20.01 | 6.04 | 5.08 | 34.79 |  | 20.00 | 7.38 | 4.84 | 35.66 |
| Δ instability activity investment |  | -1.74 | 6.72 | -20.28 | 20.14 |  | -2.04 | 5.67 | -15.90 | 9.16 |
| **Characteristic** | **M+CAU (*n* = 69)** | | | | | **CAU (*n* = 68)** | | | | |
|  |  | ***M*** | ***SD*** | ***min*** | ***max*** |  | ***M*** | ***SD*** | ***min*** | ***Max*** |
| T0 instability avoidance |  | 14.50 | 10.64 | 0.00 | 44.17 |  | 13.33 | 9.69 | 0.00 | 39.01 |
| T1 instability avoidance |  | 14.80 | 11.33 | 0.00 | 46.27 |  | 13.43 | 9.04 | 0.00 | 32.44 |
| Δ instability avoidance |  | 0.29 | 9.96 | -20.79 | 27.50 |  | 0.09 | 8.76 | -21.23 | 21.03 |
|  | ***n*** | ***M*** | ***SD*** | ***min*** | ***max*** | ***n*** | ***M*** | ***SD*** | ***min*** | ***max*** |
| T0 EI1 sadness | 39 | 0.25 | 0.40 | -0.43 | 1.33 | 44 | 0.19 | 0.49 | -0.49 | 1.70 |
| T1 EI1 sadness | 42 | 0.18 | 0.45 | -0.92 | 1.38 | 35 | 0.24 | 0.50 | -0.42 | 1.79 |
| Δ EI1 sadness |  | -0.07 | 0.60 | -1.68 | 1.29 |  | 0.05 | 0.63 | -1.70 | 1.67 |
| T0 EI1 anxiety | 27 | 0.16 | 0.27 | -0.34 | 1.01 | 25 | 0.12 | 0.28 | -0.86 | 0.93 |
| T1 EI1 anxiety | 31 | 0.15 | 0.33 | -0.56 | 1.15 | 15 | 0.07 | 0.35 | -1.56 | 1.27 |
| Δ EI1 anxiety |  | -0.02 | 0.43 | -1.48 | 1.28 |  | -0.05 | 0.43 | -1.91 | 1.19 |
| T0 EI1 stress | 49 | 0.00 | 0.47 | -1.19 | 0.99 | 49 | -0.08 | 0.46 | -1.30 | 1.03 |
| T1 EI1 stress | 49 | 0.04 | 0.48 | -0.85 | 1.57 | 46 | -0.08 | 0.45 | -1.36 | 1.14 |
| Δ EI1 stress |  | 0.05 | 0.66 | -1.42 | 1.91 |  | 0.00 | 0.60 | -1.66 | 1.61 |
| T0 EI1 anger | 27 | 0.14 | 0.28 | -0.63 | 0.91 | 25 | 0.10 | 0.28 | -0.46 | 0.84 |
| T1 EI1 anger | 25 | 0.12 | 0.32 | -0.75 | 1.34 | 23 | 0.01 | 0.34 | -1.28 | 0.81 |
| Δ EI1 anger |  | -0.02 | 0.42 | -1.23 | 0.96 |  | -0.09 | 0.48 | -1.70 | 0.81 |
| T0 EI1 positive affect | 58 | -0.47 | 0.62 | -2.12 | 1.02 | 53 | -0.59 | 0.54 | -1.91 | 0.46 |
| T1 EI1 positive affect | 51 | -0.44 | 0.53 | -1.75 | 0.84 | 52 | -0.56 | 0.54 | -2.26 | 0.18 |
| Δ EI1 positive affect |  | 0.03 | 0.77 | -1.67 | 2.12 |  | 0.03 | 0.54 | -1.29 | 1.35 |
| T0 EI1 fatigue | 52 | -0.35 | 0.34 | -1.00 | 0.32 | 51 | -0.37 | 0.35 | -1.32 | 0.35 |
| T1 EI1 fatigue | 50 | -0.32 | 0.40 | -1.53 | 0.95 | 56 | -0.35 | 0.50 | -1.53 | 1.19 |
| Δ EI1 fatigue |  | 0.03 | 0.53 | -1.18 | 1.39 |  | 0.02 | 0.49 | -0.91 | 1.50 |
| T0 EI1 loneliness | 28 | -0.01 | 0.29 | -0.85 | 0.84 | 34 | 0.12 | 0.42 | -1.29 | 1.11 |
| T1 EI1 loneliness | 33 | 0.07 | 0.40 | -1.10 | 0.96 | 29 | -0.02 | 0.36 | -1.31 | 0.95 |
| Δ EI1 loneliness |  | 0.08 | 0.45 | -1.11 | 0.92 |  | -0.13 | 0.53 | -1.57 | 1.18 |
| T0 EI1 suppression | 38 | 0.30 | 0.46 | -1.01 | 1.61 | 32 | 0.29 | 0.39 | -0.30 | 1.47 |
| T1 EI1 suppression | 34 | 0.25 | 0.43 | -0.48 | 1.79 | 36 | 0.32 | 0.60 | -0.86 | 2.74 |
| Δ EI1 suppression |  | -0.04 | 0.48 | -1.29 | 1.16 |  | 0.02 | 0.66 | -2.05 | 2.27 |
| T0 EI1 social contact | 42 | -0.02 | 0.46 | -1.11 | 1.62 | 25 | -0.06 | 0.28 | -1.21 | 0.78 |
| T1 EI1 social contact | 41 | -0.13 | 0.35 | -1.09 | 1.02 | 33 | -0.15 | 0.40 | -1.44 | 0.97 |
| Δ EI1 social contact |  | -0.10 | 0.52 | -1.62 | 1.25 |  | -0.09 | 0.39 | -1.44 | 0.97 |
| T0 EI1 activity investment | 41 | -0.04 | 0.35 | -1.24 | 0.86 | 43 | -0.20 | 0.39 | -1.64 | 1.16 |
| T1 EI1 activity investment | 50 | -0.12 | 0.48 | -1.38 | 1.26 | 49 | -0.24 | 0.45 | -1.61 | 0.75 |
| Δ EI1 activity investment |  | -0.08 | 0.49 | -1.20 | 1.30 |  | -0.04 | 0.63 | -2.30 | 1.64 |
| T0 EI1 avoidance | 26 | 0.15 | 0.31 | -0.32 | 1.15 | 24 | 0.20 | 0.33 | -0.02 | 1.35 |
| **Characteristic** | **M+CAU (*n* = 69)** | | | | | **CAU (*n* = 68)** | | | | |
|  | ***n*** | ***M*** | ***SD*** | ***min*** | ***max*** | ***N*** | ***M*** | ***SD*** | ***min*** | ***Max*** |
| T1 EI1 avoidance | 26 | 0.11 | 0.33 | -0.86 | 0.86 | 29 | 0.11 | 0.34 | -1.13 | 1.08 |
| Δ EI1 avoidance |  | -0.04 | 0.44 | -1.30 | 0.86 |  | -0.09 | 0.43 | -1.35 | 0.95 |
| T0 EI2 sadness | 39 | 0.51 | 0.62 | 0.00 | 2.34 | 44 | 0.55 | 0.68 | -0.16 | 2.36 |
| T1 EI2 sadness | 42 | 0.44 | 0.65 | -0.56 | 2.32 | 35 | 0.52 | 0.74 | -0.24 | 2.66 |
| Δ EI2 sadness |  | -0.07 | 0.85 | -2.34 | 1.71 |  | -0.03 | 0.81 | -2.36 | 2.02 |
| T0 EI2 anxiety | 27 | 0.29 | 0.46 | -0.22 | 1.59 | 25 | 0.23 | 0.41 | -0.08 | 1.62 |
| T1 EI2 anxiety | 31 | 0.33 | 0.53 | -0.46 | 2.00 | 15 | 0.21 | 0.50 | 0.00 | 2.15 |
| Δ EI2 anxiety |  | 0.04 | 0.65 | -2.05 | 1.61 |  | -0.02 | 0.58 | -1.62 | 1.99 |
| T0 EI2 stress | 49 | 0.31 | 0.54 | -0.42 | 1.96 | 49 | 0.22 | 0.46 | -0.47 | 1.79 |
| T1 EI2 stress | 49 | 0.34 | 0.55 | -0.60 | 2.05 | 46 | 0.21 | 0.48 | -0.79 | 1.89 |
| Δ EI2 stress |  | 0.03 | 0.75 | -1.75 | 2.05 |  | -0.01 | 0.62 | -1.57 | 1.83 |
| T0 EI2 anger | 27 | 0.28 | 0.48 | -0.21 | 1.71 | 25 | 0.22 | 0.45 | -0.55 | 1.45 |
| T1 EI2 anger | 25 | 0.27 | 0.52 | -0.45 | 2.22 | 23 | 0.22 | 0.48 | -0.84 | 1.80 |
| Δ EI2 anger |  | -0.01 | 0.69 | -1.71 | 1.78 |  | 0.00 | 0.70 | -1.46 | 1.61 |
| T0 EI2 positive affect | 58 | -0.12 | 0.53 | -1.17 | 1.72 | 53 | -0.23 | 0.44 | -1.19 | 0.93 |
| T1 EI2 positive affect | 51 | -0.14 | 0.47 | -1.08 | 1.15 | 52 | -0.17 | 0.43 | -1.06 | 2.06 |
| Δ EI2 positive affect |  | -0.03 | 0.66 | -1.81 | 2.29 |  | 0.06 | 0.57 | -1.10 | 2.80 |
| T0 EI2 fatigue | 52 | 0.00 | 0.38 | -0.55 | 2.01 | 51 | 0.00 | 0.31 | -0.89 | 0.77 |
| T1 EI2 fatigue | 50 | -0.02 | 0.43 | -1.17 | 2.06 | 56 | 0.10 | 0.40 | -0.45 | 1.66 |
| Δ EI2 fatigue |  | -0.01 | 0.61 | -2.49 | 2.31 |  | 0.10 | 0.48 | -0.69 | 1.57 |
| T0 EI2 loneliness | 28 | 0.16 | 0.37 | -0.33 | 1.77 | 34 | 0.36 | 0.53 | -0.49 | 1.81 |
| T1 EI2 loneliness | 33 | 0.29 | 0.52 | -0.37 | 1.59 | 29 | 0.27 | 0.56 | -0.44 | 2.43 |
| Δ EI2 loneliness |  | 0.13 | 0.60 | -1.77 | 1.59 |  | -0.10 | 0.71 | -1.81 | 1.72 |
| T0 EI2 suppression | 38 | 0.51 | 0.63 | -0.55 | 2.08 | 32 | 0.49 | 0.62 | -0.11 | 2.24 |
| T1 EI2 suppression | 34 | 0.46 | 0.60 | -0.48 | 2.01 | 36 | 0.42 | 0.62 | -0.28 | 2.07 |
| Δ EI2 suppression |  | -0.04 | 0.75 | -2.00 | 1.91 |  | -0.07 | 0.80 | -1.68 | 1.84 |
| T0 EI2 social contact | 42 | 0.08 | 0.46 | -0.63 | 1.90 | 25 | -0.02 | 0.21 | -0.66 | 0.65 |
| T1 EI2 social contact | 41 | 0.00 | 0.33 | -0.65 | 1.34 | 33 | 0.06 | 0.38 | -0.72 | 1.61 |
| Δ EI2 social contact |  | -0.08 | 0.58 | -1.90 | 1.54 |  | 0.07 | 0.46 | -0.78 | 2.27 |
| T0 EI2 activity investment | 41 | 0.08 | 0.38 | -0.53 | 1.82 | 43 | -0.07 | 0.33 | -0.50 | 1.83 |
| T1 EI2 activity investment | 50 | 0.00 | 0.40 | -0.68 | 1.48 | 49 | -0.03 | 0.31 | -0.74 | 1.17 |
| Δ EI2 activity investment |  | -0.08 | 0.49 | -1.95 | 1.25 |  | 0.04 | 0.43 | -1.83 | 1.41 |
| T0 EI2 avoidance | 26 | 0.28 | 0.46 | -0.21 | 1.62 | 24 | 0.35 | 0.56 | 0.00 | 2.37 |
| T1 EI2 avoidance | 26 | 0.27 | 0.51 | -0.63 | 1.70 | 29 | 0.41 | 0.68 | -0.11 | 2.97 |
| Δ EI2 avoidance |  | -0.02 | 0.64 | -1.55 | 1.49 |  | 0.07 | 0.87 | -2.28 | 2.76 |
| *Note.* Abbreviations: Abbreviations: CAU = care as usual, EI1 = one-step expected influence centrality, EI2 = two-step expected influence centrality, M = mean, M + CAU = intervention-modules added to care as usual, min = minimum, max = maximum, n = times depicted at group-level, SD = standard deviation, T0 = baseline assessment, T1 = post-intervention assessment, Δ = change from T0 to T1. | | | | | | | | | | |

**Fig. S3**

*Number of nodes over time*


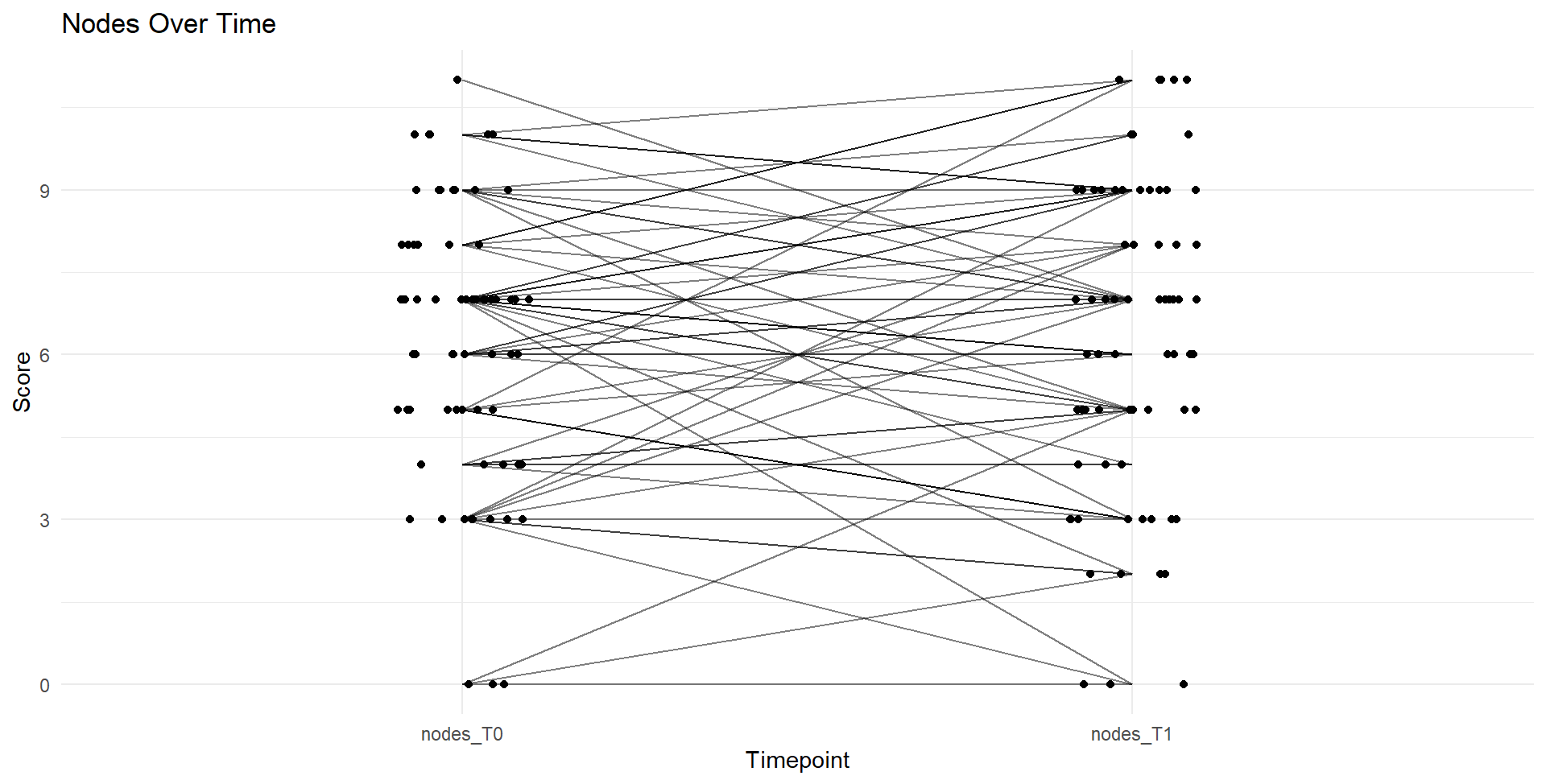


*Note*. Change from baseline to post-intervention in number of nodes are depicted for each participant with a line. Dots to the left and right of the lines indicate the number of observations at baseline or post-intervention.

## Supplement 3: Sensitivity analyses using 10-node networks

This section reports sensitivity analyses, using as basis for dependent variables a contemporaneous partial correlation network of ten nodes (*anxiety, sadness, stress, anger, positive affect, fatigue, loneliness, suppression, avoidance,* and *activity*). As opposed to the main analyses, the node *social company* was excluded in line with networks used for personalization in the randomized clinical trial (RCT). The network did not include *social company* due to data preparation steps to use SD>10 for each node, which by default was not possible for the binary item (0 = no social company, 1 = at least one other person). In total, 41 robust IWLS univariate regression analyses were performed with completed intervention-modules as independent variable and as dependent variables change from baseline to post-intervention in network density and 4×10 nodes for node dynamics (intensity, instability, one-step expected influence centrality; EI1, and two-step expected influence centrality; EI2).

### Network characteristic descriptives

The nodes most often depicted in the baseline networks of the M+CAU-group (*n* = 69) were *positive affect* (*n* = 58), *fatigue* (*n* = 56), *stress* (*n* = 47), and in post-intervention networks *positive affect* (*n* = 51), *stress* (*n* = 50)*,* and *fatigue* (*n* = 49). In the CAU-group, nodes most often depicted were *positive affect* (*n* = 53), *fatigue* (*n* = 51), *stress* (*n* = 49) at baseline, and *fatigue* (*n* = 56), *positive affect* (*n* = 52)*,* and *activity* (*n* = 49) at post-intervention. Items with the highest intensity at baseline and post-intervention were *activity, positive affect,* and *fatigue* in both groups. Instability at baseline and post-intervention was highest in the nodes *fatigue, activity,* and *stress* for both groups*.* Table S2 summarises the baseline, post-intervention, and change scores for each network characteristic, grouped by density, intensity, instability, EI1-centrality, and EI2-centrality.

### Completed modules and change in network characteristics

Using robust regression, at group level, the associations between number of completed modules and the change from baseline to post-intervention networks was assessed. A higher number of completed modules was significantly associated with a decrease from baseline to post-intervention in network density (*n* = 69;*β* = -0.009 95%CI [-0.018,0.000], *p* = .031, *η_p_^2^* = 0.02). After multiple testing correction, the statistical significance vanished.

No associations between number of modules and node-intensity (10x), -instability (10x), -EI1-centrality (10x), and -EI2-centrality (10x), were statistically significant at *p* = .05. As compared to the main analyses, the significant association between number of completed modules and centrality of *fatigue* disappeared.

### Change in network characteristics between CAU-group and modules-group

We additionally explored change in the network dynamics (Δ density, Δ intensity, Δ instability, Δ EI1 and Δ EI2 centrality) between randomization groups (M+CAU versus CAU). However, all 41 two-samples t-tests assessing differential change between the CAU-group (*n* = 67) and modules-group (*n* = 69), yielded non-significant results. Suggesting that intervention-modules added to CAU do not lead to differential change as compared to CAU.

**Table S2**

*Descriptives for network characteristics of 10-node networks per randomization group*

| **Characteristic** | **M + CAU (*n* = 69)** | | | | | **CAU (*n* = 68)** | | | | |
| --- | --- | --- | --- | --- | --- | --- | --- | --- | --- | --- |
|  |  | ***M*** | ***SD*** | ***min*** | ***max*** |  | ***M*** | ***SD*** | ***min*** | ***max*** |
| T0 density |  | 0.10 | 0.09 | 0.00 | 0.60 |  | 0.09 | 0.07 | 0.00 | 0.31 |
| T1 density |  | 0.10 | 0.08 | 0.00 | 0.53 |  | 0.11 | 0.06 | 0.00 | 0.32 |
| Δ density |  | 0.00 | 0.13 | -0.60 | 0.53 |  | 0.01 | 0.09 | -0.30 | 0.17 |
| T0 intensity sadness |  | 14.46 | 11.69 | 0.00 | 44.71 |  | 15.43 | 13.00 | 0.09 | 52.56 |
| T1 intensity sadness |  | 16.67 | 15.10 | 0.00 | 69.01 |  | 15.97 | 13.95 | 0.06 | 57.46 |
| Δ intensity sadness |  | 2.22 | 10.64 | -19.41 | 44.21 |  | 0.55 | 8.50 | -15.17 | 27.93 |
| T0 intensity anxiety |  | 11.44 | 11.11 | 0.00 | 55.85 |  | 11.16 | 11.41 | 0.00 | 39.72 |
| T1 intensity anxiety |  | 12.99 | 14.57 | 0.00 | 66.10 |  | 11.00 | 11.48 | 0.00 | 46.24 |
| Δ intensity anxiety |  | 1.55 | 7.86 | -10.64 | 27.49 |  | -0.15 | 7.16 | -21.72 | 21.62 |
| T0 intensity stress |  | 26.32 | 16.66 | 0.38 | 68.96 |  | 27.49 | 17.33 | 1.17 | 72.14 |
| T1 intensity stress |  | 27.74 | 18.07 | 0.07 | 89.97 |  | 26.02 | 16.29 | 0.01 | 66.68 |
| Δ intensity stress |  | 1.42 | 14.16 | -45.68 | 36.08 |  | -1.47 | 10.52 | -22.09 | 25.71 |
| T0 intensity anger |  | 7.68 | 8.38 | 0.00 | 45.69 |  | 9.48 | 8.40 | 0.00 | 32.96 |
| T1 intensity anger |  | 8.61 | 10.00 | 0.00 | 47.61 |  | 9.96 | 10.06 | 0.00 | 43.80 |
| Δ intensity anger |  | 0.94 | 5.29 | -14.36 | 20.66 |  | 0.49 | 7.83 | -20.42 | 39.68 |
| T0 intensity positive affect |  | 56.96 | 12.34 | 17.52 | 85.75 |  | 57.63 | 14.42 | 7.55 | 92.27 |
| T1 intensity positive affect |  | 56.46 | 14.51 | 9.01 | 87.42 |  | 56.94 | 15.68 | 7.44 | 94.39 |
| Δ intensity positive affect |  | -0.50 | 9.21 | -34.66 | 13.79 |  | -0.70 | 9.03 | -28.44 | 25.68 |
| T0 intensity fatigue |  | 49.73 | 16.86 | 5.93 | 86.33 |  | 45.49 | 14.40 | 15.89 | 83.46 |
| T1 intensity fatigue |  | 48.97 | 16.13 | 13.89 | 96.72 |  | 44.86 | 16.60 | 11.08 | 85.86 |
| Δ intensity fatigue |  | -0.77 | 10.96 | -19.28 | 30.97 |  | -0.63 | 11.95 | -44.26 | 25.73 |
| T0 intensity loneliness |  | 15.73 | 14.63 | 0.00 | 57.74 |  | 15.86 | 15.55 | 0.00 | 68.66 |
| T1 intensity loneliness |  | 16.65 | 16.74 | 0.00 | 66.63 |  | 16.66 | 16.58 | 0.00 | 61.08 |
| Δ intensity loneliness |  | 0.93 | 9.81 | -23.18 | 30.47 |  | 0.81 | 10.81 | -26.50 | 52.36 |
| T0 intensity suppression |  | 15.09 | 14.19 | 0.00 | 55.82 |  | 15.38 | 15.13 | 0.00 | 65.67 |
| T1 intensity suppression |  | 18.59 | 19.10 | 0.00 | 93.94 |  | 16.24 | 14.85 | 0.00 | 59.47 |
| Δ intensity suppression |  | 3.50 | 12.38 | -24.32 | 49.13 |  | 0.86 | 12.41 | -25.44 | 47.76 |
| T0 intensity social contact |  | 0.51 | 0.19 | 0.00 | 0.84 |  | 0.55 | 0.19 | 0.00 | 0.99 |
| T1 intensity social contact |  | 0.52 | 0.18 | 0.00 | 0.86 |  | 0.58 | 0.17 | 0.06 | 0.99 |
| Δ intensity social contact |  | 0.01 | 0.14 | -0.30 | 0.47 |  | 0.03 | 0.18 | -0.37 | 0.78 |
| T0 intensity activity investment |  | 61.95 | 8.48 | 45.54 | 83.83 |  | 63.72 | 9.71 | 41.47 | 95.81 |
| T1 intensity activity investment |  | 61.15 | 9.58 | 40.66 | 90.80 |  | 63.40 | 11.17 | 39.26 | 96.89 |
| **Characteristic** | **M + CAU (*n* = 69)** | | | | | **CAU (*n* = 67)** | | | | |
|  |  | ***M*** | ***SD*** | ***min*** | ***max*** |  | ***M*** | ***SD*** | ***min*** | ***max*** |
| Δ intensity activity investment |  | -0.80 | 6.42 | -21.14 | 17.36 |  | -0.33 | 7.01 | -15.78 | 19.15 |
| T0 intensity avoidance |  | 13.64 | 13.15 | 0.00 | 44.30 |  | 14.30 | 14.57 | 0.00 | 56.62 |
| T1 intensity avoidance |  | 16.55 | 16.57 | 0.00 | 74.42 |  | 15.65 | 14.35 | 0.00 | 60.49 |
| Δ intensity avoidance |  | 2.91 | 11.10 | -29.30 | 36.13 |  | 1.35 | 13.05 | -47.40 | 48.90 |
| T0 instability sadness |  | 15.95 | 9.48 | 0.00 | 34.97 |  | 16.00 | 8.68 | 0.76 | 35.55 |
| T1 instability sadness |  | 14.60 | 9.85 | 0.00 | 43.75 |  | 15.85 | 9.54 | 0.66 | 35.25 |
| Δ instability sadness |  | -1.35 | 10.07 | -30.39 | 43.53 |  | -0.15 | 7.02 | -17.06 | 16.42 |
| T0 instability anxiety |  | 13.40 | 9.77 | 0.00 | 58.36 |  | 12.29 | 10.07 | 0.00 | 37.44 |
| T1 instability anxiety |  | 11.54 | 9.18 | 0.00 | 34.50 |  | 11.11 | 9.57 | 0.00 | 44.76 |
| Δ instability anxiety |  | -1.85 | 7.84 | -23.87 | 20.88 |  | -1.18 | 6.80 | -17.84 | 21.43 |
| T0 instability stress |  | 21.27 | 8.40 | 1.91 | 44.11 |  | 21.29 | 8.49 | 1.54 | 40.03 |
| T1 instability stress |  | 20.83 | 8.26 | 0.98 | 40.56 |  | 20.87 | 9.38 | 0.12 | 37.45 |
| Δ instability stress |  | -0.44 | 7.23 | -17.89 | 19.80 |  | -0.42 | 7.55 | -21.41 | 17.95 |
| T0 instability anger |  | 12.57 | 9.11 | 0.00 | 41.89 |  | 13.25 | 8.73 | 0.00 | 39.63 |
| T1 instability anger |  | 11.55 | 8.50 | 0.00 | 47.03 |  | 11.92 | 7.96 | 0.00 | 32.78 |
| Δ instability anger |  | -1.02 | 9.32 | -25.72 | 47.03 |  | -1.32 | 7.49 | -23.89 | 16.51 |
| T0 instability positive affect |  | 15.70 | 5.30 | 4.69 | 34.92 |  | 15.89 | 6.03 | 4.50 | 31.44 |
| T1 instability positive affect |  | 14.59 | 4.82 | 6.36 | 29.97 |  | 15.30 | 6.31 | 3.34 | 33.63 |
| Δ instability positive affect |  | -1.11 | 4.11 | -13.57 | 11.06 |  | -0.59 | 4.18 | -11.68 | 9.39 |
| T0 instability fatigue |  | 26.47 | 7.79 | 9.25 | 52.01 |  | 27.03 | 8.94 | 8.57 | 47.96 |
| T1 instability fatigue |  | 26.34 | 8.54 | 6.86 | 44.49 |  | 25.79 | 9.15 | 10.21 | 49.19 |
| Δ instability fatigue |  | -0.12 | 7.04 | -21.01 | 17.60 |  | -1.25 | 7.21 | -18.04 | 15.06 |
| T0 instability loneliness |  | 16.39 | 10.53 | 0.00 | 35.03 |  | 16.11 | 9.41 | 0.00 | 37.35 |
| T1 instability loneliness |  | 15.40 | 11.71 | 0.00 | 49.44 |  | 15.14 | 10.23 | 0.00 | 38.11 |
| Δ instability loneliness |  | -0.99 | 10.42 | -32.86 | 38.23 |  | -0.98 | 9.09 | -22.87 | 21.13 |
| T0 instability suppression |  | 16.52 | 9.68 | 0.00 | 47.21 |  | 16.23 | 11.19 | 0.00 | 43.65 |
| T1 instability suppression |  | 16.44 | 11.34 | 0.00 | 51.20 |  | 15.33 | 10.64 | 0.00 | 46.35 |
| Δ instability suppression |  | -0.08 | 9.87 | -22.55 | 41.96 |  | -0.90 | 9.26 | -23.13 | 20.92 |
| T0 instability social contact |  | 0.56 | 0.11 | 0.00 | 0.74 |  | 0.56 | 0.13 | 0.00 | 0.85 |
| T1 instability social contact |  | 0.55 | 0.13 | 0.00 | 0.80 |  | 0.56 | 0.12 | 0.00 | 0.76 |
| Δ instability social contact |  | 0.00 | 0.11 | -0.26 | 0.31 |  | 0.00 | 0.15 | -0.47 | 0.47 |
| T0 instability activity investment |  | 21.74 | 7.29 | 5.67 | 47.25 |  | 22.06 | 6.24 | 9.28 | 40.84 |
| T1 instability activity investment |  | 20.01 | 6.04 | 5.08 | 34.79 |  | 20.26 | 7.16 | 6.37 | 35.66 |
| Δ instability activity investment |  | -1.74 | 6.72 | -20.28 | 20.14 |  | -1.79 | 5.48 | -15.54 | 9.16 |
| T0 instability avoidance |  | 14.50 | 10.64 | 0.00 | 44.17 |  | 13.53 | 9.65 | 0.00 | 39.01 |
| **Characteristic** | **M + CAU (*n* = 69)** | | | | | **CAU (*n* = 67)** | | | | |
|  | ***n*** | ***M*** | ***SD*** | ***min*** | ***max*** | ***n*** | ***M*** | ***SD*** | ***min*** | ***max*** |
| T1 instability avoidance |  | 14.80 | 11.33 | 0.00 | 46.27 |  | 13.66 | 8.95 | 0.00 | 32.44 |
| Δ instability avoidance |  | 0.29 | 9.96 | -20.79 | 27.50 |  | 0.12 | 8.82 | -21.23 | 21.03 |
| T0 EI1 sadness | 40 | 0.26 | 0.41 | -0.43 | 1.51 | 44 | 0.21 | 0.51 | -0.49 | 1.67 |
| T1 EI1 sadness | 41 | 0.22 | 0.44 | -0.66 | 1.40 | 40 | 0.25 | 0.48 | -0.41 | 1.80 |
| Δ EI1 sadness |  | -0.04 | 0.62 | -1.55 | 1.29 |  | 0.04 | 0.62 | -1.67 | 1.41 |
| T0 EI1 anxiety | 27 | 0.17 | 0.29 | -0.34 | 1.01 | 25 | 0.14 | 0.34 | -0.87 | 1.63 |
| T1 EI1 anxiety | 30 | 0.14 | 0.34 | -0.56 | 1.11 | 17 | 0.09 | 0.35 | -1.24 | 1.24 |
| Δ EI1 anxiety |  | -0.04 | 0.44 | -1.49 | 1.28 |  | -0.05 | 0.47 | -1.60 | 1.16 |
| T0 EI1 stress | 47 | -0.03 | 0.47 | -1.26 | 0.99 | 48 | -0.07 | 0.45 | -1.33 | 1.05 |
| T1 EI1 stress | 50 | 0.04 | 0.47 | -0.99 | 1.19 | 51 | -0.05 | 0.41 | -1.29 | 0.79 |
| Δ EI1 stress |  | 0.07 | 0.64 | -1.57 | 1.59 |  | 0.01 | 0.52 | -1.60 | 1.15 |
| T0 EI1 anger | 25 | 0.14 | 0.28 | -0.35 | 0.93 | 24 | 0.10 | 0.27 | -0.46 | 0.83 |
| **Characteristic** | **M + CAU (*n* = 69)** | | | | | **CAU (*n* = 67)** | | | | |
|  | ***n*** | ***M*** | ***SD*** | ***min*** | ***max*** | ***n*** | ***M*** | ***SD*** | ***min*** | ***max*** |
| T1 EI1 anger | 23 | 0.13 | 0.33 | -1.06 | 1.35 | 26 | 0.02 | 0.40 | -1.67 | 0.84 |
| Δ EI1 anger |  | -0.01 | 0.42 | -1.22 | 1.00 |  | -0.08 | 0.54 | -2.16 | 0.84 |
| T0 EI1 positive affect | 58 | -0.61 | 0.55 | -2.11 | 0.43 | 54 | -0.64 | 0.52 | -1.92 | 0.48 |
| T1 EI1 positive affect | 51 | -0.52 | 0.53 | -1.66 | 0.58 | 54 | -0.64 | 0.55 | -2.27 | 0.37 |
| Δ EI1 positive affect |  | 0.09 | 0.71 | -1.37 | 2.11 |  | 0.00 | 0.56 | -1.31 | 1.45 |
| T0 EI1 fatigue | 56 | -0.37 | 0.37 | -1.01 | 0.89 | 52 | -0.38 | 0.34 | -1.33 | 0.35 |
| T1 EI1 fatigue | 49 | -0.29 | 0.38 | -1.52 | 0.91 | 54 | -0.39 | 0.48 | -1.56 | 0.55 |
| Δ EI1 fatigue |  | 0.08 | 0.50 | -1.15 | 1.34 |  | -0.01 | 0.47 | -0.93 | 1.61 |
| T0 EI1 loneliness | 23 | 0.09 | 0.28 | -0.58 | 1.11 | 35 | 0.17 | 0.41 | -1.18 | 1.11 |
| T1 EI1 loneliness | 31 | 0.12 | 0.34 | -0.81 | 0.97 | 31 | 0.06 | 0.34 | -0.80 | 0.93 |
| Δ EI1 loneliness |  | 0.03 | 0.45 | -0.96 | 0.94 |  | -0.11 | 0.50 | -1.34 | 1.18 |
| T0 EI1 suppression | 36 | 0.26 | 0.43 | -1.02 | 1.57 | 31 | 0.29 | 0.40 | -0.31 | 1.49 |
| T1 EI1 suppression | 34 | 0.27 | 0.43 | -0.48 | 1.39 | 39 | 0.32 | 0.52 | -0.63 | 1.99 |
| Δ EI1 suppression |  | 0.00 | 0.51 | -1.24 | 1.14 |  | 0.02 | 0.64 | -2.12 | 1.91 |
| T0 EI1 social contact | 0 | 0.00 | 0.00 | 0.00 | 0.00 | 0 | 0.00 | 0.00 | 0.00 | 0.00 |
| T1 EI1 social contact | 0 | 0.00 | 0.00 | 0.00 | 0.00 | 0 | 0.00 | 0.00 | 0.00 | 0.00 |
| Δ EI1 social contact |  | 0.00 | 0.00 | 0.00 | 0.00 |  | 0.00 | 0.00 | 0.00 | 0.00 |
| T0 EI1 activity investment | 40 | 0.01 | 0.40 | -1.34 | 0.90 | 38 | -0.17 | 0.41 | -1.63 | 1.16 |
| T1 EI1 activity investment | 45 | -0.06 | 0.45 | -1.12 | 1.24 | 45 | -0.17 | 0.44 | -1.62 | 0.76 |
| Δ EI1 activity investment |  | -0.07 | 0.50 | -1.37 | 1.34 |  | 0.01 | 0.64 | -2.30 | 1.61 |
| T0 EI1 avoidance | 25 | 0.14 | 0.32 | -0.48 | 1.07 | 22 | 0.19 | 0.32 | -0.03 | 1.35 |
| **Characteristic** | **M + CAU (*n* = 69)** | | | | | **CAU (*n* = 67)** | | | | |
|  | ***n*** | ***M*** | ***SD*** | ***min*** | ***max*** | ***n*** | ***M*** | ***SD*** | ***min*** | ***max*** |
| T1 EI1 avoidance | 27 | 0.14 | 0.30 | -0.82 | 0.87 | 32 | 0.14 | 0.37 | -1.07 | 1.39 |
| Δ EI1 avoidance |  | 0.00 | 0.43 | -1.07 | 1.19 |  | -0.05 | 0.47 | -1.35 | 0.95 |
| T0 EI2 sadness | 40 | 0.55 | 0.65 | -0.02 | 2.38 | 44 | 0.61 | 0.70 | -0.12 | 2.33 |
| T1 EI2 sadness | 41 | 0.48 | 0.68 | -0.65 | 2.48 | 40 | 0.56 | 0.73 | -0.25 | 2.52 |
| Δ EI2 sadness |  | -0.07 | 0.89 | -2.38 | 1.49 |  | -0.06 | 0.84 | -2.33 | 1.86 |
| T0 EI2 anxiety | 27 | 0.31 | 0.49 | -0.22 | 1.78 | 25 | 0.26 | 0.43 | -0.08 | 1.62 |
| T1 EI2 anxiety | 30 | 0.32 | 0.54 | -0.47 | 2.31 | 17 | 0.24 | 0.52 | 0.00 | 2.19 |
| Δ EI2 anxiety |  | 0.01 | 0.66 | -2.09 | 1.65 |  | -0.02 | 0.61 | -1.62 | 1.99 |
| T0 EI2 stress | 47 | 0.32 | 0.53 | -0.40 | 1.98 | 48 | 0.23 | 0.46 | -0.47 | 1.83 |
| T1 EI2 stress | 50 | 0.34 | 0.52 | -0.38 | 1.77 | 51 | 0.24 | 0.43 | -0.58 | 1.27 |
| Δ EI2 stress |  | 0.02 | 0.70 | -1.82 | 1.75 |  | 0.01 | 0.54 | -1.16 | 1.13 |
| T0 EI2 anger | 25 | 0.28 | 0.47 | -0.20 | 1.78 | 24 | 0.21 | 0.44 | -0.56 | 1.44 |
| T1 EI2 anger | 23 | 0.28 | 0.52 | -0.62 | 2.22 | 26 | 0.21 | 0.48 | -0.78 | 1.82 |
| Δ EI2 anger |  | 0.00 | 0.71 | -1.78 | 1.90 |  | 0.00 | 0.72 | -1.72 | 1.82 |
| T0 EI2 positive affect | 58 | -0.24 | 0.45 | -1.55 | 0.67 | 54 | -0.28 | 0.43 | -1.20 | 0.97 |
| T1 EI2 positive affect | 51 | -0.21 | 0.47 | -1.21 | 0.95 | 54 | -0.21 | 0.38 | -1.05 | 1.33 |
| Δ EI2 positive affect |  | 0.03 | 0.60 | -1.60 | 2.13 |  | 0.07 | 0.49 | -0.97 | 1.96 |
| T0 EI2 fatigue | 56 | 0.03 | 0.34 | -0.54 | 1.28 | 52 | 0.03 | 0.32 | -0.68 | 1.40 |
| T1 EI2 fatigue | 49 | 0.02 | 0.38 | -0.56 | 1.80 | 54 | 0.06 | 0.41 | -0.89 | 1.51 |
| Δ EI2 fatigue |  | -0.01 | 0.54 | -1.71 | 2.05 |  | 0.03 | 0.50 | -1.41 | 1.41 |
| T0 EI2 loneliness | 23 | 0.22 | 0.40 | 0.00 | 1.71 | 35 | 0.37 | 0.55 | -0.50 | 2.00 |
| T1 EI2 loneliness | 31 | 0.32 | 0.53 | -0.68 | 1.56 | 31 | 0.32 | 0.55 | -0.48 | 1.86 |
| Δ EI2 loneliness |  | 0.10 | 0.64 | -1.67 | 1.38 |  | -0.05 | 0.72 | -2.00 | 1.57 |
| T0 EI2 suppression | 36 | 0.48 | 0.61 | -0.34 | 2.05 | 31 | 0.53 | 0.69 | -0.11 | 2.66 |
| T1 EI2 suppression | 34 | 0.48 | 0.62 | -0.61 | 1.88 | 39 | 0.48 | 0.58 | -0.23 | 2.12 |
| Δ EI2 suppression |  | 0.00 | 0.77 | -1.99 | 1.74 |  | -0.05 | 0.85 | -2.26 | 1.84 |
| T0 EI2 social contact | 0 | 0.00 | 0.00 | 0.00 | 0.00 | 0 | 0.00 | 0.00 | 0.00 | 0.00 |
| T1 EI2 social contact | 0 | 0.00 | 0.00 | 0.00 | 0.00 | 0 | 0.00 | 0.00 | 0.00 | 0.00 |
| Δ EI2 social contact |  | 0.00 | 0.00 | 0.00 | 0.00 |  | 0.00 | 0.00 | 0.00 | 0.00 |
| T0 EI2 activity investment | 40 | 0.08 | 0.34 | -0.49 | 1.30 | 38 | -0.05 | 0.34 | -0.50 | 1.82 |
| T1 EI2 activity investment | 45 | 0.05 | 0.40 | -0.75 | 1.40 | 45 | -0.03 | 0.29 | -0.75 | 0.63 |
| Δ EI2 activity investment |  | -0.03 | 0.44 | -1.30 | 1.24 |  | 0.02 | 0.43 | -1.82 | 1.13 |
| T0 EI2 avoidance | 25 | 0.28 | 0.48 | 0.00 | 2.19 | 22 | 0.34 | 0.57 | 0.00 | 2.44 |
| T1 EI2 avoidance | 27 | 0.30 | 0.49 | -0.57 | 1.72 | 32 | 0.44 | 0.64 | -0.07 | 2.99 |
| Δ EI2 avoidance |  | 0.02 | 0.63 | -2.00 | 1.43 |  | 0.09 | 0.83 | -2.29 | 1.92 |
| *Note*. Abbreviations: CAU = care as usual, EI1 = one-step expected influence centrality, EI2 = two-step expected influence centrality, M = mean, M + CAU = intervention-modules added to care as usual, min = minimum, max = maximum, *n* = times depicted at group-level, SD = standard deviation, T0 = baseline assessment, T1 = post-intervention assessment, Δ = change from T0 to T1. | | | | | | | | | | |

## Supplement 4: Sensitivity analyses using a network with fewer nodes

This section reports sensitivity analyses using a network with maximally six instead of eleven nodes. Contemporaneous partial correlation networks of 6 nodes were constructed using aggregated scores for *negative affect* (anxiety, sadness anger, stress), *positive affect* (relaxed, energetic, enthusiastic, cheerful), *activity investment* (enjoyable activity, activity that costed energy), *avoidance* (suppression, avoidance), and the EMA items *fatigue*, and *loneliness*. *Social company* was left out of the analyses, because we wanted to restrict the number of nodes to maximally six, as suggested by Mansueto and colleagues [17]. See Figure S3 for example networks at T0 and T1 using maximally 6 nodes.

Here, we report the descriptives (Table S3) and results based on the sample randomized to intervention-modules + CAU (M+CAU), that showed T0 and T1 data for the networks with fewer nodes (*n* = 68). As compared to the main analyses, one participant was removed because no network was available during T1. The results after multiple testing correction were unchanged. However, there were two difference before the correction *(*i.e. when using *α =* .05). The number of completed intervention-modules was not associated with change in centrality of *fatigue* or with change in *network density* in networks with fewer nodes.

### Results

#### Completed modules and change in network characteristics

No association between number of intervention-modules and average-level change in node intensity (6×), instability (6×), one-step or two-step expected influence centrality (2×6), or network density was evident.

#### Difference between randomization groups

No significant difference was evident for change in network dynamics between the M+CAU and CAU group.

**Table S3**

*Characteristics of six-node network at baseline, post-intervention, and change-score per randomization group*

| **Characteristic** | **M+CAU (*n* = 68)** | | | | | **CAU (*n* = 67)** | | | | |
| --- | --- | --- | --- | --- | --- | --- | --- | --- | --- | --- |
|  |  | ***M*** | ***SD*** | ***Min*** | ***max*** |  | ***M*** | ***SD*** | ***min*** | ***max*** |
| T0 density |  | 0.14 | 0.09 | 0.00 | 0.46 |  | 0.16 | 0.11 | 0.00 | 0.49 |
| T1 density |  | 0.15 | 0.09 | 0.00 | 0.40 |  | 0.16 | 0.12 | 0.00 | 0.55 |
| Δ density |  | 0.01 | 0.11 | -0.25 | 0.28 |  | 0.00 | 0.15 | -0.45 | 0.44 |
| T0 mean negative affect |  | 15.06 | 10.54 | 0.13 | 44.23 |  | 15.71 | 10.82 | 0.97 | 43.78 |
| T1 mean negative affect |  | 16.68 | 13.07 | 0.02 | 53.20 |  | 15.58 | 11.67 | 0.17 | 51.76 |
| Δ mean negative affect |  | 1.62 | 8.24 | -14.11 | 27.56 |  | -0.13 | 6.93 | -15.95 | 25.98 |
| T0 mean positive affect |  | 56.58 | 12.02 | 17.52 | 85.75 |  | 58.13 | 14.79 | 7.55 | 92.27 |
| T1 mean positive affect |  | 56.00 | 14.12 | 9.01 | 84.68 |  | 57.39 | 16.02 | 7.44 | 94.39 |
| Δ mean positive affect |  | -0.58 | 9.26 | -34.66 | 13.79 |  | -0.74 | 9.02 | -28.44 | 25.68 |
| T0 mean fatigue |  | 50.25 | 16.43 | 5.93 | 86.33 |  | 44.78 | 14.82 | 13.00 | 83.46 |
| T1 mean fatigue |  | 49.48 | 15.67 | 21.00 | 96.72 |  | 44.24 | 16.87 | 11.08 | 85.86 |
| Δ mean fatigue |  | -0.77 | 11.04 | -19.28 | 30.97 |  | -0.55 | 11.96 | -44.26 | 25.73 |
| T0 mean loneliness |  | 15.86 | 14.70 | 0.00 | 57.74 |  | 15.58 | 15.64 | 0.00 | 68.66 |
| T1 mean loneliness |  | 16.85 | 16.79 | 0.00 | 66.63 |  | 16.48 | 16.67 | 0.00 | 61.08 |
| Δ mean loneliness |  | 0.98 | 9.87 | -23.18 | 30.47 |  | 0.90 | 10.78 | -26.50 | 52.36 |
| T0 mean activity |  | 61.66 | 8.18 | 45.54 | 83.83 |  | 64.31 | 9.98 | 41.47 | 95.81 |
| T1 mean activity |  | 60.81 | 9.22 | 40.66 | 90.80 |  | 64.04 | 11.51 | 39.26 | 96.89 |
| Δ mean activity |  | -0.84 | 6.46 | -21.14 | 17.36 |  | -0.27 | 7.02 | -15.78 | 19.15 |
| T0 mean avoidance |  | 14.46 | 13.18 | 0.04 | 50.06 |  | 14.61 | 14.38 | 0.00 | 59.23 |
| T1 mean avoidance |  | 17.78 | 17.15 | 0.00 | 84.18 |  | 15.74 | 14.34 | 0.00 | 55.88 |
| Δ mean avoidance |  | 3.32 | 11.07 | -21.79 | 42.63 |  | 1.13 | 12.28 | -35.16 | 47.78 |
| T0 instability negative affect |  | 11.24 | 5.75 | 0.71 | 32.76 |  | 11.12 | 5.84 | 1.57 | 27.59 |
| T1 instability negative affect |  | 10.26 | 5.35 | 0.25 | 27.70 |  | 10.35 | 5.40 | 0.49 | 25.82 |
| Δ instability negative affect |  | -0.98 | 5.24 | -17.89 | 21.78 |  | -0.77 | 4.00 | -10.98 | 7.48 |
| T0 instability positive affect |  | 15.86 | 5.17 | 5.83 | 34.92 |  | 15.86 | 6.07 | 4.50 | 31.44 |
| T1 instability positive affect |  | 14.68 | 4.79 | 6.36 | 29.97 |  | 15.23 | 6.38 | 3.34 | 33.63 |
| Δ instability positive affect |  | -1.18 | 4.10 | -13.57 | 11.06 |  | -0.63 | 4.16 | -11.68 | 9.39 |
| T0 instability fatigue |  | 26.72 | 7.56 | 12.33 | 52.01 |  | 27.17 | 8.72 | 8.57 | 47.96 |
| T1 instability fatigue |  | 26.57 | 8.39 | 6.86 | 44.49 |  | 25.76 | 9.17 | 10.21 | 49.19 |
| Δ instability fatigue |  | -0.15 | 7.09 | -21.01 | 17.60 |  | -1.41 | 7.04 | -18.04 | 15.06 |
| T0 instability loneliness |  | 16.59 | 10.48 | 0.00 | 35.03 |  | 15.99 | 9.54 | 0.00 | 37.35 |
| T1 instability loneliness |  | 15.61 | 11.67 | 0.00 | 49.44 |  | 15.01 | 10.34 | 0.00 | 38.11 |
| **Characteristic** | **M+CAU (*n* = 68)** | | | | | **CAU (*n* = 67)** | | | | |
|  |  | ***M*** | ***SD*** | ***min*** | ***max*** |  | ***M*** | ***SD*** | ***min*** | ***max*** |
| Δ instability loneliness |  | -0.98 | 10.49 | -32.86 | 38.23 |  | -0.98 | 9.09 | -22.87 | 21.13 |
| T0 instability activity |  | 21.91 | 7.21 | 5.67 | 47.25 |  | 22.20 | 6.09 | 9.28 | 40.84 |
| T1 instability activity |  | 20.02 | 6.09 | 5.08 | 34.79 |  | 20.13 | 7.36 | 4.84 | 35.66 |
| Δ instability activity |  | -1.89 | 6.65 | -20.28 | 20.14 |  | -2.07 | 5.71 | -15.90 | 9.16 |
| T0 instability avoidance |  | 12.76 | 7.62 | 0.28 | 36.95 |  | 12.40 | 8.30 | 0.00 | 31.96 |
| T1 instability avoidance |  | 12.77 | 8.40 | 0.00 | 30.85 |  | 11.80 | 7.76 | 0.00 | 29.40 |
| Δ instability avoidance |  | 0.01 | 7.46 | -18.36 | 26.39 |  | -0.60 | 6.75 | -16.60 | 17.43 |
| **Characteristic** | **M+CAU (*n* = 68)** | | | | | **CAU (*n* = 67)** | | | | |
|  | ***n*** | ***M*** | ***SD*** | ***min*** | ***max*** | ***n*** | ***M*** | ***SD*** | ***min*** | ***max*** |
| T0 EI1 negative affect | 34 | -0.09 | 0.37 | -1.03 | 0.92 | 29 | -0.10 | 0.33 | -0.98 | 0.78 |
| T1 EI1 negative affect | 26 | -0.02 | 0.42 | -1.11 | 1.24 | 25 | -0.08 | 0.36 | -1.38 | 0.72 |
| Δ EI1 negative affect |  | 0.06 | 0.55 | -1.82 | 1.24 |  | 0.01 | 0.47 | -2.16 | 0.92 |
| T0 EI1 positive affect | 61 | -0.54 | 0.47 | -1.47 | 0.51 | 56 | -0.56 | 0.50 | -1.65 | 0.43 |
| T1 EI1 positive affect | 50 | -0.46 | 0.49 | -1.66 | 0.64 | 55 | -0.50 | 0.54 | -2.12 | 0.49 |
| Δ EI1 positive affect |  | 0.08 | 0.65 | -1.27 | 1.35 |  | 0.06 | 0.47 | -1.25 | 1.32 |
| T0 EI1 fatigue | 53 | -0.38 | 0.33 | -1.23 | 0.84 | 52 | -0.40 | 0.33 | -1.15 | 0.49 |
| T1 EI1 fatigue | 48 | -0.32 | 0.32 | -1.08 | 0.37 | 50 | -0.36 | 0.34 | -1.30 | 0.40 |
| Δ EI1 fatigue |  | 0.06 | 0.44 | -1.00 | 1.23 |  | 0.04 | 0.34 | -0.74 | 0.91 |
| T0 EI1 loneliness | 23 | 0.06 | 0.26 | -0.90 | 0.94 | 34 | 0.04 | 0.39 | -0.84 | 1.11 |
| T1 EI1 loneliness | 24 | 0.01 | 0.27 | -0.83 | 0.96 | 28 | 0.01 | 0.31 | -0.77 | 0.86 |
| Δ EI1 loneliness |  | -0.05 | 0.35 | -0.94 | 0.89 |  | -0.03 | 0.51 | -1.10 | 1.22 |
| T0 EI1 activity | 41 | -0.06 | 0.49 | -1.40 | 0.79 | 43 | -0.13 | 0.47 | -1.63 | 0.87 |
| T1 EI1 activity | 42 | -0.04 | 0.49 | -1.26 | 1.25 | 40 | -0.09 | 0.50 | -1.43 | 0.96 |
| Δ EI1 activity |  | 0.02 | 0.63 | -1.57 | 1.40 |  | 0.04 | 0.59 | -1.58 | 1.39 |
| T0 EI1 avoidance | 29 | 0.10 | 0.36 | -0.60 | 1.02 | 26 | 0.12 | 0.32 | -0.48 | 1.22 |
| T1 EI1 avoidance | 32 | 0.13 | 0.33 | -0.48 | 1.34 | 32 | 0.10 | 0.38 | -0.61 | 1.22 |
| Δ EI1 avoidance |  | 0.03 | 0.41 | -1.38 | 0.97 |  | -0.02 | 0.44 | -0.99 | 1.22 |
| T0 EI2 negative affect | 34 | 0.19 | 0.44 | -0.57 | 1.53 | 29 | 0.21 | 0.41 | -0.30 | 1.62 |
| T1 EI2 negative affect | 26 | 0.19 | 0.41 | -0.25 | 1.70 | 25 | 0.16 | 0.32 | -0.25 | 1.33 |
| Δ EI2 negative affect |  | 0.00 | 0.45 | -1.21 | 1.23 |  | -0.05 | 0.47 | -1.62 | 1.33 |
| T0 EI2 positive affect | 61 | -0.10 | 0.38 | -0.90 | 0.76 | 56 | -0.16 | 0.37 | -0.87 | 0.78 |
| T1 EI2 positive affect | 50 | -0.11 | 0.40 | -1.05 | 0.84 | 55 | -0.09 | 0.41 | -1.20 | 0.74 |
| Δ EI2 positive affect |  | 0.00 | 0.51 | -1.15 | 1.23 |  | 0.07 | 0.44 | -1.33 | 1.25 |
| T0 EI2 fatigue | 53 | -0.01 | 0.34 | -0.54 | 1.40 | 52 | -0.04 | 0.25 | -0.60 | 0.61 |
| T1 EI2 fatigue | 48 | 0.01 | 0.33 | -0.55 | 1.11 | 50 | 0.00 | 0.31 | -0.59 | 1.09 |
| **Characteristic** | **M+CAU (*n* = 68)** | | | | | **CAU (*n* = 67)** | | | | |
|  | ***n*** | ***M*** | ***SD*** | ***min*** | ***max*** | ***n*** | ***M*** | ***SD*** | ***min*** | ***max*** |
| Δ EI2 fatigue |  | 0.01 | 0.43 | -0.97 | 1.11 |  | 0.03 | 0.36 | -0.78 | 0.85 |
| T0 EI2 loneliness | 23 | 0.12 | 0.24 | -0.11 | 0.91 | 34 | 0.22 | 0.36 | -0.44 | 1.18 |
| T1 EI2 loneliness | 24 | 0.17 | 0.37 | -0.25 | 1.50 | 28 | 0.17 | 0.39 | -0.46 | 1.86 |
| Δ EI2 loneliness |  | 0.05 | 0.40 | -0.91 | 1.50 |  | -0.05 | 0.49 | -1.02 | 1.96 |
| T0 EI2 activity | 41 | 0.04 | 0.35 | -0.53 | 1.05 | 43 | 0.00 | 0.32 | -0.50 | 1.25 |
| T1 EI2 activity | 42 | 0.04 | 0.35 | -0.50 | 1.02 | 40 | 0.06 | 0.33 | -0.50 | 1.03 |
| Δ EI2 activity |  | 0.00 | 0.43 | -1.08 | 1.30 |  | 0.06 | 0.37 | -1.25 | 1.11 |
| T0 EI2 avoidance | 29 | 0.22 | 0.42 | -0.28 | 1.61 | 26 | 0.19 | 0.32 | -0.23 | 1.17 |
| T1 EI2 avoidance | 32 | 0.27 | 0.40 | -0.36 | 1.28 | 32 | 0.20 | 0.39 | -0.48 | 1.54 |
| Δ EI2 avoidance |  | 0.04 | 0.47 | -1.61 | 1.18 |  | 0.01 | 0.47 | -0.96 | 1.54 |
| *Note.* Abbreviations: CAU = care as usual, EI1 = one-step expected influence centrality, EI2 = two-step expected influence centrality, M = mean, M + CAU = intervention-modules added to care as usual, min = minimum, max = maximum, *n* = times depicted at group-level, SD = standard deviation, T0 = baseline assessment, T1 = post-intervention assessment, Δ = change from T0 to T1. | | | | | | | | | | |

**Fig. S3**

*Exemplar networks at T0 and T1 of participant 1726 using maximally 6 nodes*

| 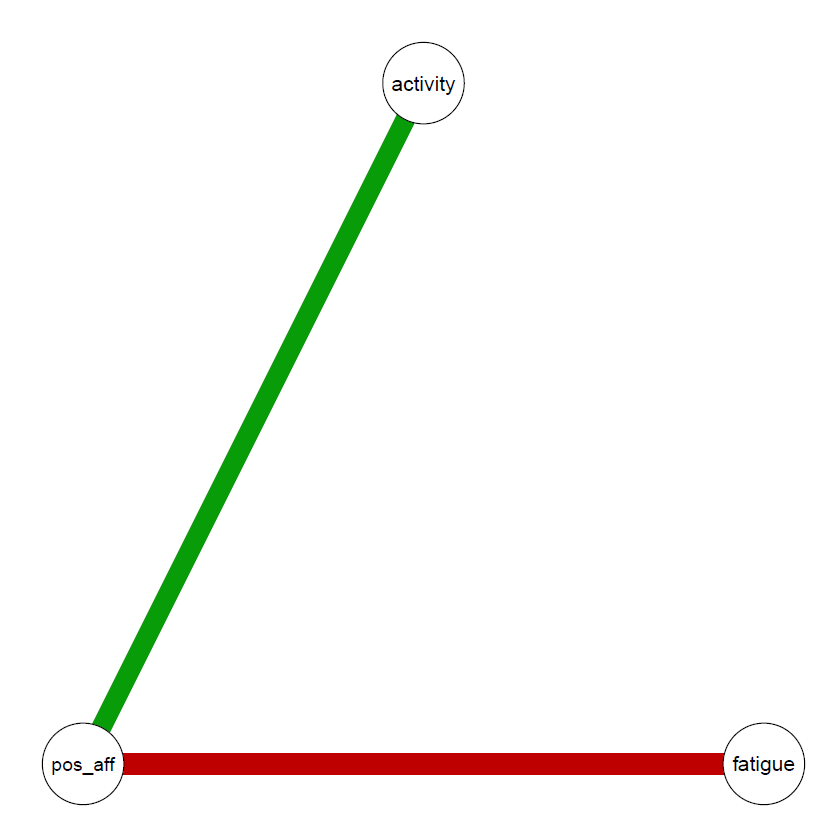 |
| --- |
| 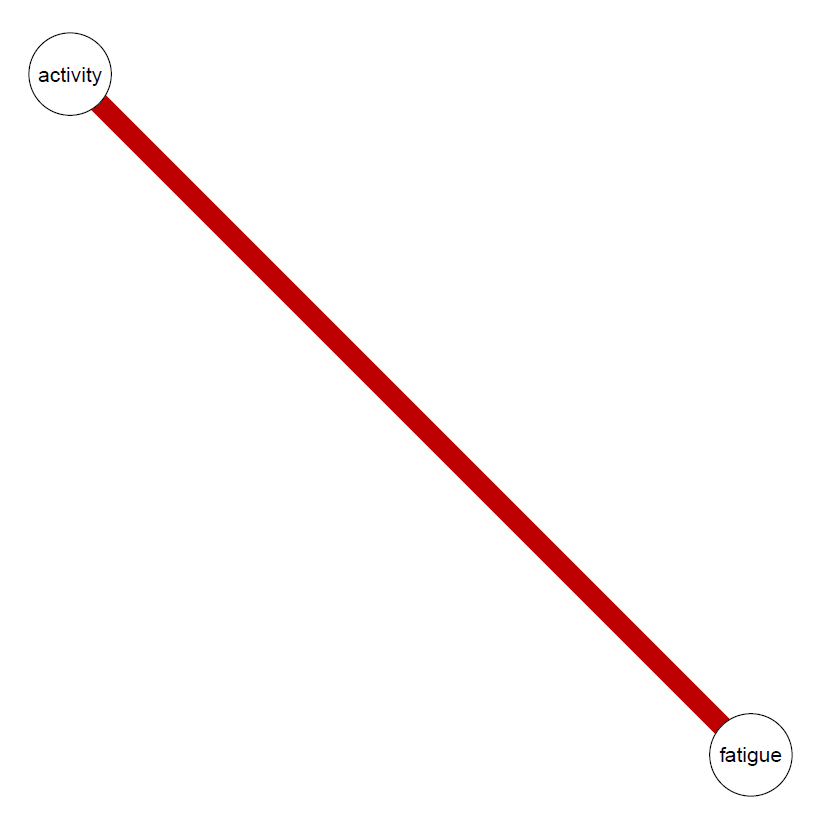 |

*Note.* Depicted nodes are Activity (which is an aggregate of the items ‘my current activity was enjoyable, my current activity costed energy’, Fatigue (I feel… tired), and Positive affect (Pos_aff; which is an aggregate of the items ‘I feel… relaxed, energetic, enthusiastic, cheerful’). Red lines depict a negative partial correlation, green lines depict a positive partial correlation. Thicker lines resemble stronger partial correlations.

## Supplement 5: Sensitivity analyses using participants that adhered and deviated from the personalized data-driven advice

This section reports sensitivity analyses in which nineteen participants were removed. As described in the manuscript, participants could deviated from the personalized data-driven advice based on shared decision making. Here, we report the descriptives and results based on the sample that adhered to the personalized data-driven advice (*n* = 50). The sample (see Table S4 and S5) was comparable to the main sample (see Table 1 and 2 in the manuscript) in terms of age, sex, compliance with EMA, and completed intervention-modules. The results after multiple testing correction were unchanged. However, there were two differences before the correction. In the smaller sample, the number of completed intervention-modules was associated with instability of *activity investment*, and was not associated with *network density* at *α =* .05.

### Results

#### Choice and completion of modules

Sixty-nine participants were randomized to intervention-modules + CAU (M+CAU) and provided sufficient (>30%) EMA-data at baseline and post-intervention. Nineteen of 69 participants (27.5%) were excluded because they deviated from the data-driven advice regarding combinations of intervention-modules. Table S4 shows sample characteristics.

For included participants (*n* = 50) most often advised intervention-modules were *Wellness* (*n* = 37), *Activation* (*n* = 37), and *Enhancing positive affect* (*n* = 31). One participant did not complete any intervention-module at the post-intervention assessments, and all others finished 1 to 6 intervention-modules (M[SD] = 4.34[1.76]). Four in five participants (*n* = 40;80%) completed at least half of the intervention (>3 intervention-modules), and more than half of the participants (*n* = 28;56%) started the last intervention-module (StayFine relapse prevention plan). Table S5 summarises the number of chosen and completed modules.

#### Network characteristic descriptives

On average, change in network dynamics at group level was minimal (between -2% and 3% change), see Table S6. In the group of participants randomized to intervention-modules (*n* = 50; M+CAU-group), the nodes that were most often depicted in the baseline networks of 11 nodes were *positive affect* (*n* = 43), *fatigue* (*n* = 39), and *stress* (*n* = 33). In the post-intervention networks, nodes most often depicted were *positive affect* (*n* = 51), *activity* (*n* = 50), and *fatigue* (*n* = 50). Nodes with the highest intensity were *activity, positive affect,* and *fatigue* at baseline and post-intervention. At baseline and post-intervention, node-instability was highest in the nodes *fatigue, activity,* and *stress*. On group level, the most-central nodes were *sadness* and *suppression* based on centrality values (bootstrapping methods for centrality ordering were not performed). Table S4 summarises the network characteristics at baseline, post-intervention, and the change-score, grouped by intensity, instability, EI1-, EI2-centrality, and density.

#### Completed modules and change in network characteristics

No association between number of completed intervention-modules and average-level change in node intensity (11×). Associations between a higher number of completed intervention-modules and change in node instability were non-significant (10×), except one that showed an increase in average-level instability of the node *activity investment* (*n* = 50;*β* = 1.079, 95%CI [0.000,2.157], *p* = .020, *η_p_^2^* = 0.11). An increase in instability suggests that fluctuation from one moment to the next increases for *activity investment.* The significance of the result vanished after correcting for multiple testing.

On average, a higher number of completed intervention-modules was associated with a decrease in centrality of *fatigue* [*n* = 50;*β* = -0.071 95%CI (-0.142,0.000), *p* = .037, *η_p_^2^* = 0.04]. A negative change in node-EI2-centrality means that the node was less influential in the network (taking into account the relative strength of neighbouring nodes) when more intervention-modules were completed. An analysis without two outliers in node-EI2-centrality of *fatigue* did not change the results (*p* = .032). Again, after correcting for multiple testing, the results became non-significant. The remainder of associations between number of intervention-modules and the change from baseline to post-intervention in one-step expected influence centrality (11×), and two-step expected influence centrality (10×) was statistically non-significant.

A higher number of completed intervention-modules was not associated with an average-level decrease in network density.

#### Additional analyses

Randomization groups were compared for change in each network dynamics (45×). Of 45 t-tests for differences between randomization groups (M+CAU versus CAU), two significantly showed a difference: one-step EI-centrality of *loneliness* (*t*[113.68] = -2.46, *p* = .015, d = -0.45; M_M+CAU_[SD] = 0.09[0.45]; M_CAU_[SD] = -0.13[0.53]), and two-step EI-centrality of *loneliness* (*t*[115.47] = -2.42, *p* = .017, d = -0.43; M_M+CAU_[SD] = 0.19(0.56); M_CAU_[SD] = -0.10(0.71]). Reflecting a mean positive change (increase) in the M+CAU-group, and a mean negative change (decrease) in the CAU-group. However, results were non-significant after multiple testing correction.

**Table S4**

*Sample characteristics for participants that adhered to the personalization advice*

|  | **Sample (*N* = 118)** | | | | **M+CAU (*n* = 50)** | | | | **CAU (*n* = 68)** | | | |
| --- | --- | --- | --- | --- | --- | --- | --- | --- | --- | --- | --- | --- |
|  | **No.** | **%** |  |  | **No.** | **%** |  |  | **No.** | **%** |  |  |
| Female | 106 | 89.8 |  |  | 43 | 86 |  |  | 63 | 93 |  |  |
| Prior episodes |  |  |  |  |  |  |  |  |  |  |  |  |
| 1 | 81 | 68.6 |  |  | 33 | 66 |  |  | 48 | 71 |  |  |
| 2 | 28 | 23.7 |  |  | 14 | 28 |  |  | 14 | 21 |  |  |
| 3 | 7 | 5.9 |  |  | 3 | 6 |  |  | 4 | 6 |  |  |
| 4 | 2 | 1.7 |  |  | 0 | 0 |  |  | 2 | 3 |  |  |
| Prior disorders* |  |  |  |  |  |  |  |  |  |  |  |  |
| Depressive disorders only | 22 | 18.6 |  |  | 13 | 26 |  |  | 9 | 13 |  |  |
| Major depressive disorder | 21 | 17.8 |  |  | 13 | 26 |  |  | 8 | 12 |  |  |
| Persistent depressive disorder | 2 | 1.7 |  |  | 0 | 0 |  |  | 2 | 3 |  |  |
| Anxiety disorders only | 12 | 10.2 |  |  | 4 | 8 |  |  | 8 | 12 |  |  |
| Agoraphobia | 7 | 5.9 |  |  | 2 | 4 |  |  | 5 | 7 |  |  |
| Generalized anxiety disorder | 9 | 7.6 |  |  | 3 | 6 |  |  | 6 | 9 |  |  |
| Panic disorder | 5 | 4.2 |  |  | 2 | 4 |  |  | 3 | 4 |  |  |
| Social anxiety disorder | 4 | 3.4 |  |  | 2 | 4 |  |  | 2 | 3 |  |  |
| Separation anxiety disorder | 3 | 2.5 |  |  | 1 | 2 |  |  | 2 | 3 |  |  |
| Specific phobia | 4 | 3.4 |  |  | 0 | 0 |  |  | 4 | 6 |  |  |
| Anxiety and Depressive disorders | 84 | 71.2 |  |  | 33 | 66 |  |  | 51 | 75 |  |  |
| Major depressive disorder | 81 | 68.6 |  |  | 32 | 64 |  |  | 49 | 72 |  |  |
| Persistent depressive disorder | 8 | 6.8 |  |  | 1 | 2 |  |  | 7 | 10 |  |  |
| Agoraphobia | 24 | 20.3 |  |  | 6 | 12 |  |  | 18 | 26 |  |  |
| Generalized anxiety disorder | 56 | 47.5 |  |  | 24 | 48 |  |  | 32 | 47 |  |  |
| Panic disorder | 39 | 33.1 |  |  | 13 | 26 |  |  | 26 | 38 |  |  |
| Separation anxiety disorder | 14 | 11.9 |  |  | 3 | 6 |  |  | 11 | 16 |  |  |
| Social anxiety disorder | 51 | 43.2 |  |  | 19 | 38 |  |  | 32 | 47 |  |  |
|  | **Sample (*N* = 118)** | | | | **M+CAU (*n* = 50)** | | | | **CAU (*n* = 68)** | | | |
|  | **No.** | **%** |  |  | **No.** | **%** |  |  | **No.** | **%** |  |  |
| Specific phobia | 29 | 24.6 |  |  | 14 | 28 |  |  | 15 | 22 |  |  |
| Other disorders^@^ | 68 | 57.6 |  |  | 25 | 50 |  |  | 43 | 63 |  |  |
| One | 34 | 28.8 |  |  | 15 | 30 |  |  | 19 | 28 |  |  |
| Two | 23 | 19.5 |  |  | 8 | 16 |  |  | 15 | 22 |  |  |
| Three | 10 | 8.5 |  |  | 2 | 4 |  |  | 8 | 12 |  |  |
| Four | 1 | 0.8 |  |  | 0 | 0 |  |  | 1 | 1 |  |  |
| Prior psychological treatment | 106 | 89.8 |  |  | 43 | 86 |  |  | 63 | 93 |  |  |
| Current other disorder(s) | 21 | 17.8 |  |  | 5 | 10 |  |  |  | 0 |  |  |
| Current infrequent psychological treatment | 14 | 11.9 |  |  | 6 | 12 |  |  | 8 | 12 |  |  |
| Current medication^#^ | 51 | 43.2 |  |  | 28 | 56 |  |  | 23 | 34 |  |  |
| For depression or anxiety | 24 | 20.3 |  |  | 13 | 26 |  |  | 11 | 16 |  |  |
| For other reasons | 34 | 28.8 |  |  | 21 | 42 |  |  | 13 | 19 |  |  |
| Dutch nationality birth father | 104 | 88.1 |  |  | 42 | 84 |  |  | 62 | 91 |  |  |
| Dutch nationality birth mother | 106 | 89.8 |  |  | 43 | 86 |  |  | 63 | 93 |  |  |
| Dutch nationality both parents | 101 | 85.6 |  |  | 41 | 82 |  |  | 60 | 88 |  |  |
|  | ***M*** | ***SD*** | ***min*** | ***max*** | ***M*** | ***SD*** | ***min*** | ***max*** | ***M*** | ***SD*** | ***min*** | ***max*** |
| Age | 19.70 | 1.77 | 14.33 | 22.42 | 19.68 | 1.78 | 14.92 | 22.08 | 19.72 | 1.76 | 14.33 | 22.42 |
| T0 to T1 (weeks) | 18.99 | 5.49 | 11.00 | 46.01 | 20.39 | 5.70 | 11.99 | 37.99 | 17.95 | 5.12 | 11.00 | 46.01 |
| T0 entries | 71.20 | 10.49 | 38.00 | 84.00 | 71.26 | 9.81 | 50.00 | 84.00 | 71.16 | 11.04 | 38.00 | 84.00 |
| T0 entries p/d | 5.09 | 0.75 | 2.71 | 6.00 | 5.09 | 0.70 | 3.57 | 6.00 | 5.08 | 0.79 | 2.71 | 6.00 |
| T0 entries p/d SD | 0.85 | 0.47 | 0.00 | 3.11 | 0.86 | 0.42 | 0.00 | 1.79 | 0.85 | 0.51 | 0.00 | 3.11 |
| T1 entries | 59.94 | 16.03 | 25.00 | 83.00 | 60.46 | 15.99 | 29.00 | 83.00 | 59.56 | 16.17 | 25.00 | 83.00 |
| T1 entries p/d | 4.28 | 1.15 | 1.79 | 5.93 | 4.32 | 1.14 | 2.07 | 5.93 | 4.25 | 1.15 | 1.79 | 5.93 |
| T1 entries p/d SD | 1.23 | 0.60 | 0.27 | 3.03 | 1.16 | 0.54 | 0.27 | 2.35 | 1.28 | 0.65 | 0.27 | 3.03 |
| *Note.* Abbreviations: CAU = care as usual, M = mean, M + CAU = intervention-modules added to care as usual, min = minimum, max = maximum, No. = number, p/d = per day, SD = standard deviation, T0 = baseline assessment, T1 = post-intervention assessment  * As assessed with the Kiddie-Schedule for Affective Disorders and Schizophrenia present and lifetime (K-SADS-PL DSM-5).  ^@^ Assessed disorders were psychotic-, and eating disorders, obsessive compulsive disorder, posttraumatic stress disorder, (hypo)mania, and alcohol and drug disorders.  ^#^ numbers do not add up to, since some have combined medication for depressive or anxiety disorders, and other reasons. | | | | | | | | | | | | |

**Table S5**

*Number of chosen and completed interventions (total and per combination) for participants that adhered to the personalization advice*

|  | **Completed number of intervention-modules** | | | | | | | | | | | | | | | | | | | |
| --- | --- | --- | --- | --- | --- | --- | --- | --- | --- | --- | --- | --- | --- | --- | --- | --- | --- | --- | --- | --- |
| **No. int-modules** | **0** | **1** | | **1.5** | | **2** | | **2.5** | **3** | | **3.5** | | **4** | | **4.5** | **5** | | **5.5** | | **6** |
| (*n* = 50) | 1 | 1 | | 5 | | 2 | | 1 | 4 | | 3 | | 5 | | 1 | 5 | | 4 | | 18 |
|  | **Completed per combination** | | | | | | | | | | | | | | | | | | | |
| **Intervention-module** | Completed (total) | | Positive affect, activation, sleep (*n*=7) | | Positive affect, activation, wellness (*n*=10) | | Wellness, activation, sleep (*n*=8) | | | Positive affect, exposure, sleep (*n*=4) | | Positive affect, exposure, wellness (*n*=8) | | Wellness, exposure, sleep (*n*=1) | | | Positive affect, activation, exposure (*n*=2) | | Wellness, activation, exposure (*n*=10) | |
|  |  |  |  |  |  |  |  |  |  |  |  |  |  |  |  |  |  |  |  |  |
| PE (*n* = 50) | 49 | | 7 | | 9 | | 8 | | | 4 | | 8 | | 1 | | | 2 | | 10 | |
| CR (*n* = 50) | 42 | | 6 | | 8^@^ | | 8 | | | 2 | | 7 | | 1 | | | 1 | | 9 | |
| AC (*n* = 37) | 25 | | 4 | | 9 | | 5 | | | - | | - | | - | | | 0 | | 7 | |
| EX (*n* = 25) | 13 | | - | | - | | - | | | 1 | | 6 | | 1 | | | 0 | | 5 | |
| PA (*n* = 31) | 24 | | 5 | | 9 | | - | | | 2 | | 7 | | - | | | 1 | | - | |
| SL (*n* = 20) | 10 | | 4 | | - | | 5 | | | 1 | | - | | 0 | | | - | | - | |
| WN (*n* = 37) | 28 | | - | | 8 | | 7 | | | - | | 4 | | 1 | | | - | | 8 | |
| SF (*n* = 50) | 18 | | 4 | | 5 | | 3 | | | 1 | | 2 | | 0 | | | 0 | | 3 | |
| *Note.* The number of times an intervention-module was chosen is noted on the left, with *n* = x after each intervention-module. Then, the number of completed intervention-modules is reported in total, followed by how many were completed in each combination. The subsample size is noted with *n*=x for each combination of intervention-modules.  Abbreviations: *n* = times chosen, PE = psychoeducation, CR = cognitive restructuring, AC = behavioral activation, EX = exposure, PA = enhancing positive affect, SL = sleep, WN = wellness, SF = StayFine plan.  *combination was not based on the data-driven advice.  ^@^=1 participant was never offered part 2 of the intervention-module and therefore did not complete the entire module. | | | | | | | | | | | | | | | | | | | | |

**Table S6**

*Network characteristics at baseline, post-intervention, and change-score for participants that adhered to the personalization advice*

| **Characteristic** | **M+CAU (*n* = 50)** | | | | | **CAU (*n* = 68)** | | | | | |
| --- | --- | --- | --- | --- | --- | --- | --- | --- | --- | --- | --- |
|  |  | ***M*** | ***SD*** | ***min*** | ***max*** |  | ***M*** | ***SD*** | ***min*** | ***max*** | |
| T0 density |  | 0.08 | 0.05 | 0.00 | 0.23 |  | 0.08 | 0.05 | 0.00 | 0.27 | |
| T1 density |  | 0.08 | 0.05 | 0.00 | 0.29 |  | 0.09 | 0.05 | 0.00 | 0.26 | |
| Δ density |  | 0.00 | 0.06 | -0.20 | 0.17 |  | 0.01 | 0.07 | -0.20 | 0.20 | |
| T0 intensity sadness |  | 14.20 | 11.94 | 0.00 | 44.71 |  | 15.10 | 13.05 | 0.09 | 52.56 | |
| T1 intensity sadness |  | 15.37 | 13.02 | 0.00 | 48.72 |  | 15.68 | 13.99 | 0.06 | 57.46 | |
| Δ intensity sadness |  | 1.18 | 8.31 | -19.41 | 21.11 |  | 0.57 | 8.42 | -15.17 | 27.93 | |
| T0 intensity anxiety |  | 11.10 | 11.43 | 0.00 | 55.85 |  | 10.91 | 11.42 | 0.00 | 39.72 | |
| T1 intensity anxiety |  | 11.82 | 13.90 | 0.00 | 66.10 |  | 10.83 | 11.46 | 0.00 | 46.24 | |
| Δ intensity anxiety |  | 0.72 | 6.88 | -10.64 | 27.29 |  | -0.08 | 7.10 | -21.72 | 21.62 | |
| T0 intensity stress |  | 25.78 | 17.14 | 0.38 | 68.96 |  | 26.96 | 17.53 | 1.17 | 72.14 | |
| T1 intensity stress |  | 25.90 | 17.33 | 0.07 | 89.97 |  | 25.51 | 16.48 | 0.01 | 66.68 | |
| Δ intensity stress |  | 0.12 | 13.14 | -45.68 | 36.08 |  | -1.45 | 10.45 | -22.09 | 25.71 | |
| T0 intensity anger |  | 6.97 | 6.78 | 0.00 | 31.00 |  | 9.30 | 8.41 | 0.00 | 32.96 | |
| T1 intensity anger |  | 7.46 | 8.09 | 0.00 | 43.73 |  | 9.77 | 10.07 | 0.00 | 43.80 | |
| Δ intensity anger |  | 0.49 | 4.46 | -12.35 | 14.81 |  | 0.47 | 7.78 | -20.42 | 39.68 | |
| T0 intensity positive affect |  | 55.75 | 13.19 | 17.52 | 85.75 |  | 58.40 | 14.84 | 7.55 | 92.27 | |
| T1 intensity positive affect |  | 55.19 | 14.71 | 9.01 | 87.42 |  | 57.63 | 16.03 | 7.44 | 94.39 | |
| Δ intensity positive affect |  | -0.56 | 9.26 | -34.66 | 13.07 |  | -0.77 | 8.96 | -28.44 | 25.68 | |
| T0 intensity fatigue |  | 49.76 | 16.73 | 13.59 | 86.33 |  | 44.54 | 14.84 | 13.00 | 83.46 | |
| T1 intensity fatigue |  | 48.14 | 14.80 | 13.89 | 96.72 |  | 43.89 | 16.98 | 11.08 | 85.86 | |
| Δ intensity fatigue |  | -1.63 | 8.42 | -19.28 | 15.12 |  | -0.65 | 11.91 | -44.26 | 25.73 | |
| T0 intensity loneliness |  | 13.50 | 12.40 | 0.00 | 45.63 |  | 15.51 | 15.53 | 0.00 | 68.66 | |
| T1 intensity loneliness |  | 14.35 | 13.46 | 0.00 | 48.38 |  | 16.28 | 16.63 | 0.00 | 61.08 | |
| Δ intensity loneliness |  | 0.85 | 8.45 | -23.18 | 30.47 |  | 0.77 | 10.76 | -26.50 | 52.36 | |
| T0 intensity suppression |  | 14.43 | 13.91 | 0.00 | 55.82 |  | 15.10 | 15.13 | 0.00 | 65.67 | |
| **Characteristic** | **M+CAU (*n* = 50)** | | | | | **CAU (*n* = 68)** | | | | | |
|  |  | ***M*** | ***SD*** | ***min*** | ***max*** |  | ***M*** | ***SD*** | ***min*** | ***max*** | |
| T1 intensity suppression |  | 16.19 | 15.93 | 0.00 | 58.35 |  | 15.97 | 14.86 | 0.00 | 59.47 | |
| Δ intensity suppression |  | 1.77 | 10.77 | -24.32 | 41.32 |  | 0.88 | 12.31 | -25.44 | 47.76 | |
| T0 intensity social contact |  | 52.90 | 19.00 | 0.00 | 83.82 |  | 54.93 | 19.73 | 0.00 | 98.72 | |
| T1 intensity social contact |  | 53.72 | 18.62 | 0.00 | 85.71 |  | 57.70 | 17.19 | 5.56 | 98.73 | |
| Δ intensity social contact |  | 0.82 | 14.44 | -30.39 | 46.57 |  | 2.76 | 18.26 | -36.66 | 77.93 | |
| T0 intensity activity investment |  | 60.70 | 8.10 | 45.54 | 81.98 |  | 64.53 | 10.08 | 41.47 | 95.81 | |
| T1 intensity activity investment |  | 59.80 | 7.93 | 40.66 | 84.24 |  | 64.20 | 11.51 | 39.26 | 96.89 | |
| Δ intensity activity investment |  | -0.90 | 5.76 | -17.62 | 11.29 |  | -0.34 | 6.99 | -15.78 | 19.15 | |
| T0 intensity avoidance |  | 12.76 | 12.78 | 0.00 | 44.30 |  | 13.96 | 14.54 | 0.00 | 56.62 | |
| T1 intensity avoidance |  | 14.17 | 14.05 | 0.00 | 53.56 |  | 15.28 | 14.36 | 0.00 | 60.49 | |
| Δ intensity avoidance |  | 1.41 | 10.31 | -29.30 | 22.29 |  | 1.32 | 12.95 | -47.40 | 48.90 | |
| T0 instability sadness |  | 15.53 | 9.53 | 0.00 | 34.97 |  | 15.79 | 8.78 | 0.76 | 35.55 | |
| T1 instability sadness |  | 14.88 | 9.94 | 0.00 | 43.75 |  | 15.62 | 9.64 | 0.66 | 35.25 | |
| Δ instability sadness |  | -0.66 | 9.34 | -18.26 | 43.53 |  | -0.17 | 6.97 | -17.06 | 16.42 | |
| T0 instability anxiety |  | 12.95 | 10.48 | 0.00 | 58.36 |  | 12.16 | 10.06 | 0.00 | 37.44 | |
| T1 instability anxiety |  | 11.58 | 9.11 | 0.00 | 34.50 |  | 10.95 | 9.57 | 0.00 | 44.76 | |
| Δ instability anxiety |  | -1.37 | 7.81 | -23.87 | 20.88 |  | -1.21 | 6.74 | -17.84 | 21.43 | |
| T0 instability stress |  | 20.42 | 8.41 | 1.91 | 43.87 |  | 21.24 | 8.42 | 1.54 | 40.03 | |
| T1 instability stress |  | 19.94 | 8.61 | 0.98 | 40.56 |  | 20.56 | 9.58 | 0.12 | 37.45 | |
| Δ instability stress |  | -0.48 | 6.70 | -13.58 | 19.80 |  | -0.68 | 7.57 | -21.41 | 17.95 | |
| T0 instability anger |  | 12.19 | 9.18 | 0.00 | 41.89 |  | 13.15 | 8.75 | 0.00 | 39.63 | |
| T1 instability anger |  | 11.05 | 9.27 | 0.00 | 47.03 |  | 11.85 | 7.98 | 0.00 | 32.78 | |
| Δ instability anger |  | -1.15 | 9.72 | -25.72 | 47.03 |  | -1.30 | 7.44 | -23.89 | 16.51 | |
| T0 instability positive affect |  | 15.46 | 5.06 | 4.69 | 27.28 |  | 15.77 | 6.07 | 4.50 | 31.44 | |
| T1 instability positive affect |  | 14.32 | 4.45 | 6.36 | 26.70 |  | 15.09 | 6.44 | 3.34 | 33.63 | |
| Δ instability positive affect |  | -1.15 | 3.31 | -11.48 | 4.91 |  | -0.68 | 4.16 | -11.68 | 9.39 | |
| T0 instability fatigue |  | 25.66 | 7.26 | 9.25 | 45.46 |  | 27.13 | 8.66 | 8.57 | 47.96 | |
| T1 instability fatigue |  | 26.13 | 7.87 | 9.06 | 43.02 |  | 25.51 | 9.33 | 8.93 | 49.19 | |
| **Characteristic** | **M+CAU (*n* = 50)** | | | | | **CAU (*n* = 68)** | | | | | |
|  |  | ***M*** | ***SD*** | ***min*** | ***max*** |  | ***M*** | ***SD*** | ***min*** | ***max*** | |
| Δ instability fatigue |  | 0.47 | 6.88 | -21.01 | 17.60 |  | -1.62 | 7.19 | -18.04 | 15.06 | |
| T0 instability loneliness |  | 15.52 | 10.42 | 0.00 | 35.03 |  | 15.84 | 9.55 | 0.00 | 37.35 | |
| T1 instability loneliness |  | 15.09 | 11.94 | 0.00 | 49.44 |  | 14.83 | 10.36 | 0.00 | 38.11 | |
| Δ instability loneliness |  | -0.44 | 10.19 | -23.94 | 38.23 |  | -1.01 | 9.02 | -22.87 | 21.13 | |
| T0 instability suppression |  | 16.39 | 10.25 | 0.00 | 47.21 |  | 16.00 | 11.26 | 0.00 | 43.65 | |
| T1 instability suppression |  | 16.34 | 11.58 | 0.00 | 51.20 |  | 15.13 | 10.68 | 0.00 | 46.35 | |
| Δ instability suppression |  | -0.05 | 10.28 | -22.55 | 41.96 |  | -0.87 | 9.20 | -23.13 | 20.92 | |
| T0 instability social contact |  | 55.58 | 12.29 | 0.00 | 74.05 |  | 56.12 | 12.96 | 0.00 | 84.85 | |
| T1 instability social contact |  | 55.19 | 13.29 | 0.00 | 79.77 |  | 55.45 | 11.47 | 0.00 | 76.38 | |
| Δ instability social contact |  | -0.39 | 11.44 | -26.09 | 31.01 |  | -0.67 | 14.43 | -46.55 | 46.67 | |
| T0 instability activity investment |  | 21.47 | 6.66 | 5.67 | 35.96 |  | 22.05 | 6.17 | 9.28 | 40.84 | |
| T1 instability activity investment |  | 19.49 | 5.68 | 5.08 | 32.61 |  | 20.00 | 7.38 | 4.84 | 35.66 | |
| Δ instability activity investment |  | -1.99 | 6.62 | -20.28 | 20.14 |  | -2.04 | 5.67 | -15.90 | 9.16 | |
| T0 instability avoidance |  | 14.21 | 11.12 | 0.00 | 44.17 |  | 13.33 | 9.69 | 0.00 | 39.01 | |
| T1 instability avoidance |  | 13.94 | 10.49 | 0.00 | 46.27 |  | 13.43 | 9.04 | 0.00 | 32.44 | |
| Δ instability avoidance |  | -0.27 | 10.07 | -20.79 | 27.50 |  | 0.09 | 8.76 | -21.23 | 21.03 | |
| **Characteristic** | **M+CAU (*n* = 50)** | | | | | **CAU (*n* = 68)** | | | | | |
|  | ***n*** | ***M*** | ***SD*** | ***min*** | ***max*** | ***n*** | ***M*** | ***SD*** | ***min*** | ***max*** | |
| T0 EI1 sadness | 27 | 0.21 | 0.35 | -0.34 | 1.31 | 44 | 0.19 | 0.49 | -0.49 | 1.70 | |
| T1 EI1 sadness | 33 | 0.28 | 0.47 | -0.92 | 1.38 | 35 | 0.24 | 0.50 | -0.42 | 1.79 | |
| Δ EI1 sadness |  | 0.06 | 0.57 | -1.45 | 1.29 |  | 0.05 | 0.63 | -1.70 | 1.67 | |
| T0 EI1 anxiety | 17 | 0.15 | 0.25 | 0.00 | 1.01 | 25 | 0.12 | 0.28 | -0.86 | 0.93 | |
| T1 EI1 anxiety | 22 | 0.15 | 0.33 | -0.46 | 1.15 | 15 | 0.07 | 0.35 | -1.56 | 1.27 | |
| Δ EI1 anxiety |  | 0.01 | 0.38 | -1.31 | 0.81 |  | -0.05 | 0.43 | -1.91 | 1.19 | |
| T0 EI1 stress | 33 | -0.01 | 0.47 | -1.19 | 0.99 | 49 | -0.08 | 0.46 | -1.30 | 1.03 | |
| T1 EI1 stress | 34 | 0.07 | 0.48 | -0.85 | 1.57 | 46 | -0.08 | 0.45 | -1.36 | 1.14 | |
| Δ EI1 stress |  | 0.09 | 0.63 | -1.29 | 1.91 |  | 0.00 | 0.60 | -1.66 | 1.61 | |
| T0 EI1 anger | 18 | 0.13 | 0.29 | -0.63 | 0.91 | 25 | 0.10 | 0.28 | -0.46 | 0.84 | |
| **Characteristic** | **M+CAU (*n* = 50)** | | | | | **CAU (*n* = 68)** | | | | | |
|  | ***n*** | ***M*** | ***SD*** | ***min*** | ***max*** | ***n*** | ***M*** | ***SD*** | ***min*** | ***max*** | |
| T1 EI1 anger | 14 | 0.11 | 0.34 | -0.75 | 1.34 | 23 | 0.01 | 0.34 | -1.28 | 0.81 | |
| Δ EI1 anger |  | -0.02 | 0.42 | -1.23 | 0.96 |  | -0.09 | 0.48 | -1.70 | 0.81 | |
| T0 EI1 positive affect | 43 | -0.44 | 0.56 | -1.54 | 0.92 | 53 | -0.59 | 0.54 | -1.91 | 0.46 | |
| T1 EI1 positive affect | 36 | -0.40 | 0.49 | -1.66 | 0.59 | 52 | -0.56 | 0.54 | -2.26 | 0.18 | |
| Δ EI1 positive affect |  | 0.03 | 0.72 | -1.26 | 1.71 |  | 0.03 | 0.54 | -1.29 | 1.35 | |
| T0 EI1 fatigue | 39 | -0.37 | 0.34 | -1.00 | 0.32 | 51 | -0.37 | 0.35 | -1.32 | 0.35 | |
| T1 EI1 fatigue | 39 | -0.35 | 0.41 | -1.53 | 0.95 | 56 | -0.35 | 0.50 | -1.53 | 1.19 | |
| Δ EI1 fatigue |  | 0.02 | 0.51 | -1.18 | 1.39 |  | 0.02 | 0.49 | -0.91 | 1.50 | |
| T0 EI1 loneliness | 20 | -0.03 | 0.30 | -0.85 | 0.84 | 34 | 0.12 | 0.42 | -1.29 | 1.11 | |
| T1 EI1 loneliness | 24 | 0.06 | 0.41 | -1.10 | 0.96 | 29 | -0.02 | 0.36 | -1.31 | 0.95 | |
| Δ EI1 loneliness |  | 0.09 | 0.45 | -1.11 | 0.92 |  | -0.13 | 0.53 | -1.57 | 1.18 | |
| T0 EI1 suppression | 26 | 0.28 | 0.46 | -1.01 | 1.61 | 32 | 0.29 | 0.39 | -0.30 | 1.47 | |
| T1 EI1 suppression | 25 | 0.25 | 0.41 | -0.48 | 1.34 | 36 | 0.32 | 0.60 | -0.86 | 2.74 | |
| Δ EI1 suppression |  | -0.03 | 0.51 | -1.29 | 1.16 |  | 0.02 | 0.66 | -2.05 | 2.27 | |
| T0 EI1 social contact | 32 | -0.05 | 0.50 | -1.11 | 1.62 | 25 | -0.06 | 0.28 | -1.21 | 0.78 | |
| T1 EI1 social contact | 26 | -0.16 | 0.33 | -1.09 | 0.42 | 33 | -0.15 | 0.40 | -1.44 | 0.97 | |
| Δ EI1 social contact |  | -0.10 | 0.53 | -1.62 | 1.25 |  | -0.09 | 0.39 | -1.44 | 0.97 | |
| T0 EI1 activity investment | 32 | -0.06 | 0.37 | -1.24 | 0.61 | 43 | -0.20 | 0.39 | -1.64 | 1.16 | |
| T1 EI1 activity investment | 37 | -0.12 | 0.52 | -1.38 | 1.26 | 49 | -0.24 | 0.45 | -1.61 | 0.75 | |
| Δ EI1 activity investment |  | -0.07 | 0.51 | -1.14 | 1.30 |  | -0.04 | 0.63 | -2.30 | 1.64 | |
| T0 EI1 avoidance | 20 | 0.17 | 0.33 | -0.32 | 1.15 | 24 | 0.20 | 0.33 | -0.02 | 1.35 | |
| T1 EI1 avoidance | 20 | 0.13 | 0.35 | -0.86 | 0.82 | 29 | 0.11 | 0.34 | -1.13 | 1.08 | |
| Δ EI1 avoidance |  | -0.03 | 0.45 | -1.30 | 0.82 |  | -0.09 | 0.43 | -1.35 | 0.95 | |
| T0 EI2 sadness | 27 | 0.47 | 0.57 | 0.00 | 2.34 | 44 | 0.55 | 0.68 | -0.16 | 2.36 | |
| T1 EI2 sadness | 33 | 0.57 | 0.69 | -0.56 | 2.32 | 35 | 0.52 | 0.74 | -0.24 | 2.66 | |
| Δ EI2 sadness |  | 0.10 | 0.81 | -2.34 | 1.71 |  | -0.03 | 0.81 | -2.36 | 2.02 | |
| T0 EI2 anxiety | 17 | 0.27 | 0.42 | 0.00 | 1.52 | 25 | 0.23 | 0.41 | -0.08 | 1.62 | |
| T1 EI2 anxiety | 22 | 0.35 | 0.55 | -0.04 | 2.00 | 15 | 0.21 | 0.50 | 0.00 | 2.15 | |
| **Characteristic** | **M+CAU (*n* = 50)** | | | | | **CAU (*n* = 68)** | | | | | |
|  | ***n*** | ***M*** | ***SD*** | ***min*** | ***max*** | ***n*** | ***M*** | ***SD*** | ***min*** | ***max*** | |
| Δ EI2 anxiety |  | 0.08 | 0.61 | -1.56 | 1.61 |  | -0.02 | 0.58 | -1.62 | 1.99 | |
| T0 EI2 stress | 33 | 0.28 | 0.55 | -0.42 | 1.96 | 49 | 0.22 | 0.46 | -0.47 | 1.79 | |
| T1 EI2 stress | 34 | 0.35 | 0.57 | -0.60 | 2.05 | 46 | 0.21 | 0.48 | -0.79 | 1.89 | |
| Δ EI2 stress |  | 0.07 | 0.74 | -1.73 | 2.05 |  | -0.01 | 0.62 | -1.57 | 1.83 | |
| T0 EI2 anger | 18 | 0.23 | 0.42 | -0.21 | 1.71 | 25 | 0.22 | 0.45 | -0.55 | 1.45 | |
| T1 EI2 anger | 14 | 0.24 | 0.53 | -0.45 | 2.22 | 23 | 0.22 | 0.48 | -0.84 | 1.80 | |
| Δ EI2 anger |  | 0.02 | 0.64 | -1.71 | 1.78 |  | 0.00 | 0.70 | -1.46 | 1.61 | |
| T0 EI2 positive affect | 43 | -0.08 | 0.48 | -1.14 | 1.17 | 53 | -0.23 | 0.44 | -1.19 | 0.93 | |
| T1 EI2 positive affect | 36 | -0.10 | 0.42 | -1.03 | 1.15 | 52 | -0.17 | 0.43 | -1.06 | 2.06 | |
| Δ EI2 positive affect |  | -0.02 | 0.65 | -1.76 | 2.29 |  | 0.06 | 0.57 | -1.10 | 2.80 | |
| T0 EI2 fatigue | 39 | -0.04 | 0.41 | -0.55 | 2.01 | 51 | 0.00 | 0.31 | -0.89 | 0.77 | |
| T1 EI2 fatigue | 39 | -0.05 | 0.44 | -1.17 | 2.06 | 56 | 0.10 | 0.40 | -0.45 | 1.66 | |
| Δ EI2 fatigue |  | -0.02 | 0.67 | -2.49 | 2.31 |  | 0.10 | 0.48 | -0.69 | 1.57 | |
| T0 EI2 loneliness | 20 | 0.13 | 0.34 | -0.33 | 1.12 | 34 | 0.36 | 0.53 | -0.49 | 1.81 | |
| T1 EI2 loneliness | 24 | 0.32 | 0.55 | -0.37 | 1.59 | 29 | 0.27 | 0.56 | -0.44 | 2.43 | |
| Δ EI2 loneliness |  | 0.19 | 0.56 | -0.88 | 1.59 |  | -0.10 | 0.71 | -1.81 | 1.72 | |
| T0 EI2 suppression | 26 | 0.48 | 0.66 | -0.55 | 2.08 | 32 | 0.49 | 0.62 | -0.11 | 2.24 | |
| T1 EI2 suppression | 25 | 0.47 | 0.61 | -0.48 | 2.00 | 36 | 0.42 | 0.62 | -0.28 | 2.07 | |
| Δ EI2 suppression |  | -0.02 | 0.81 | -2.00 | 1.91 |  | -0.07 | 0.80 | -1.68 | 1.84 | |
| T0 EI2 social contact | 32 | 0.05 | 0.46 | -0.63 | 1.90 | 25 | -0.02 | 0.21 | -0.66 | 0.65 | |
| T1 EI2 social contact | 26 | -0.01 | 0.31 | -0.65 | 1.08 | 33 | 0.06 | 0.38 | -0.72 | 1.61 | |
| Δ EI2 social contact |  | -0.06 | 0.58 | -1.90 | 1.54 |  | 0.07 | 0.46 | -0.78 | 2.27 | |
| T0 EI2 activity investment | 32 | 0.10 | 0.37 | -0.53 | 1.82 | 43 | -0.07 | 0.33 | -0.50 | 1.83 | |
| T1 EI2 activity investment | 37 | 0.01 | 0.41 | -0.68 | 1.48 | 49 | -0.03 | 0.31 | -0.74 | 1.17 | |
| Δ EI2 activity investment |  | -0.09 | 0.46 | -1.51 | 1.25 |  | 0.04 | 0.43 | -1.83 | 1.41 | |
| T0 EI2 avoidance | 20 | 0.30 | 0.46 | -0.21 | 1.46 | 24 | 0.35 | 0.56 | 0.00 | 2.37 | |
| T1 EI2 avoidance | 20 | 0.31 | 0.54 | -0.63 | 1.70 | 29 | 0.41 | 0.68 | -0.11 | 2.97 | |
| Δ EI2 avoidance |  | 0.01 | 0.63 | -1.41 | 1.49 |  | 0.07 | 0.87 | -2.28 | 2.76 | |
| *Note.* Abbreviations: CAU = care as usual, EI1 = one-step expected influence centrality, EI2 = two-step expected influence centrality, M = mean, M + CAU = intervention-modules added to care as usual, min = minimum, max = maximum, *n* = times depicted at group-level, SD = standard deviation, T0 = baseline assessment, T1 = post-intervention assessment, Δ = change from T0 to T1. | | | | | | | | | | |  |

## Supplement 6 R-code

*R-code as used for data-preparation*
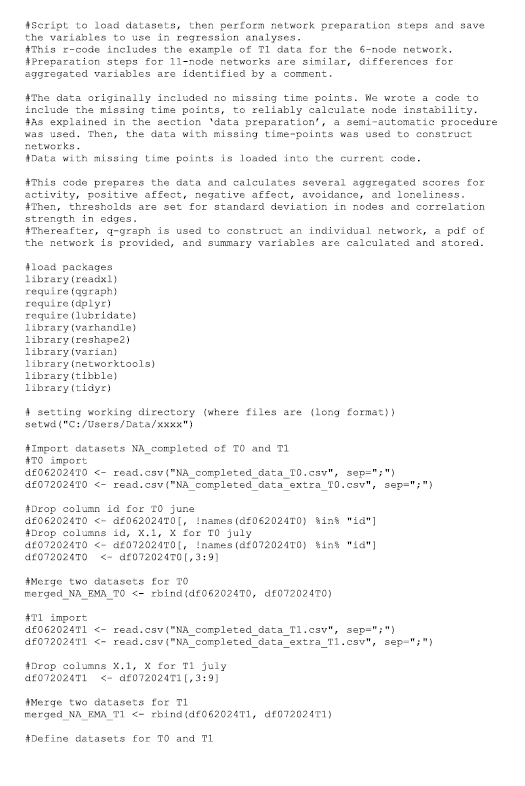


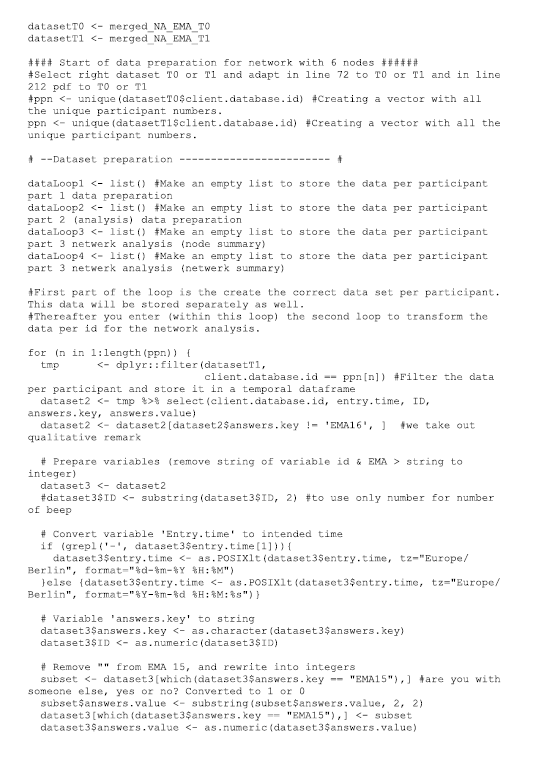


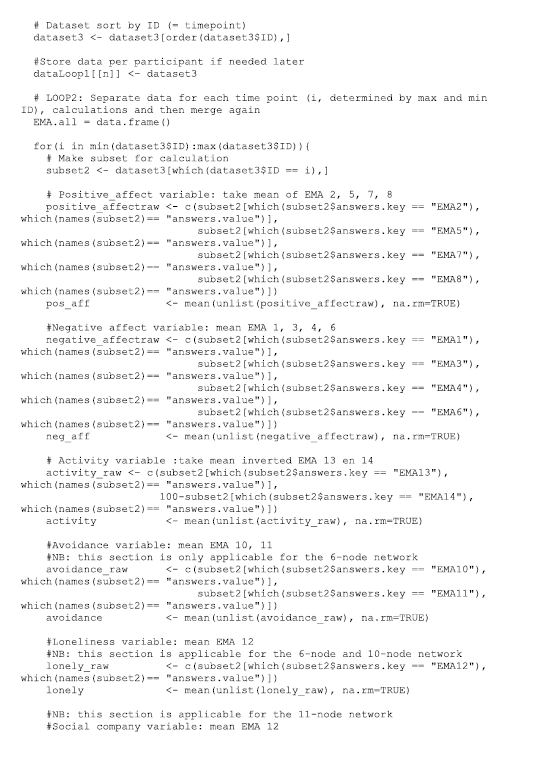


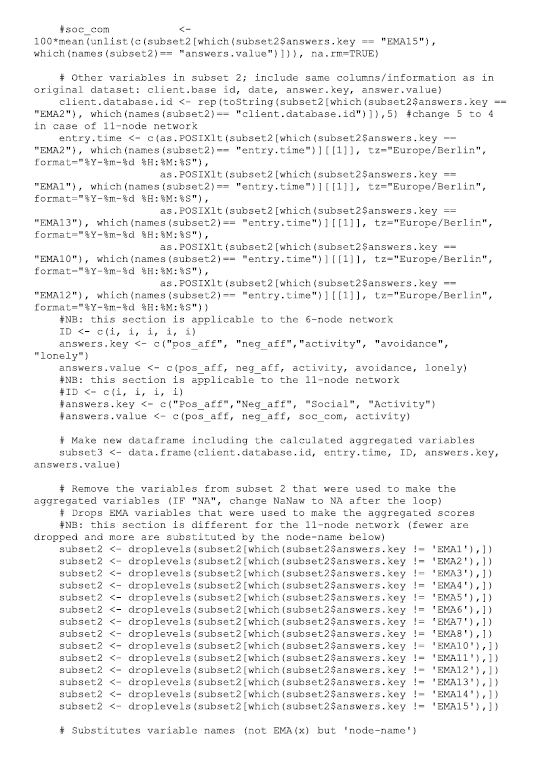


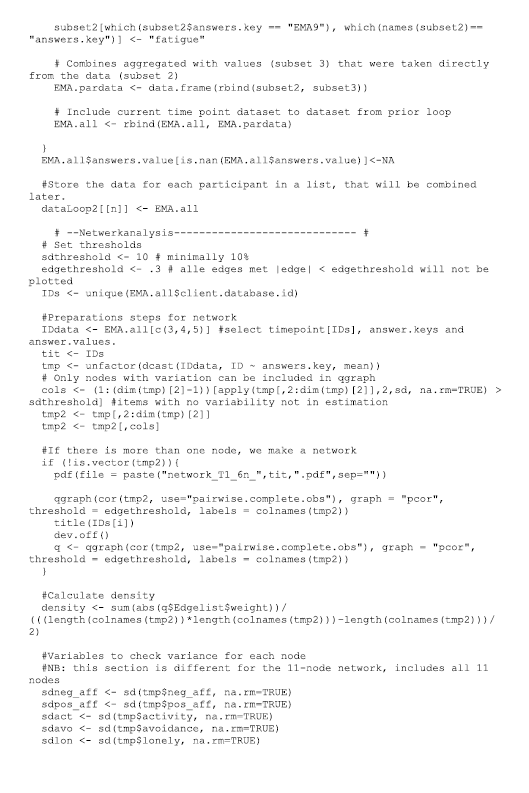


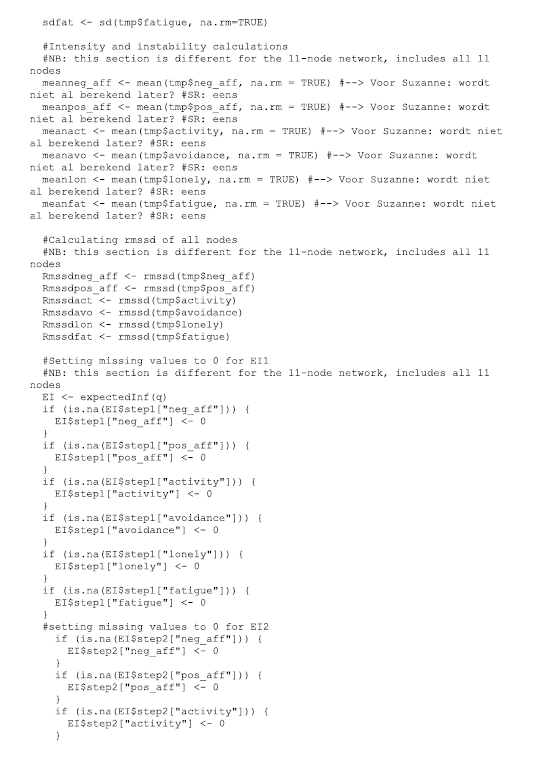


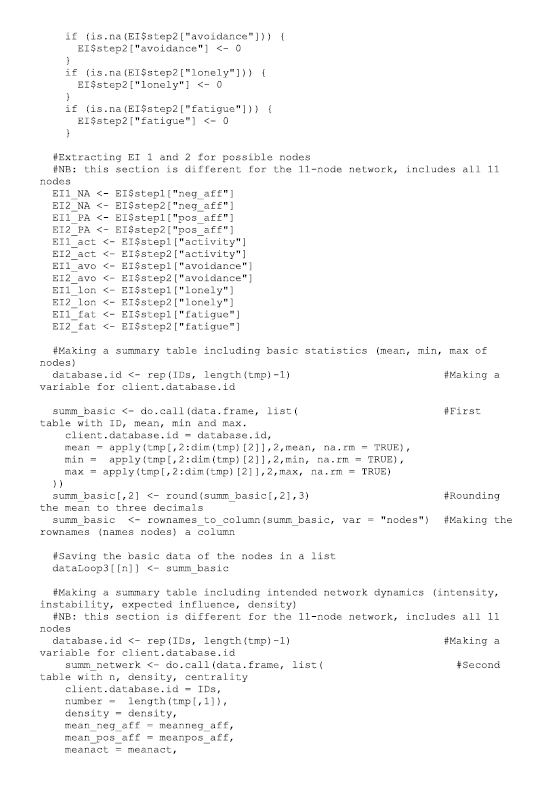


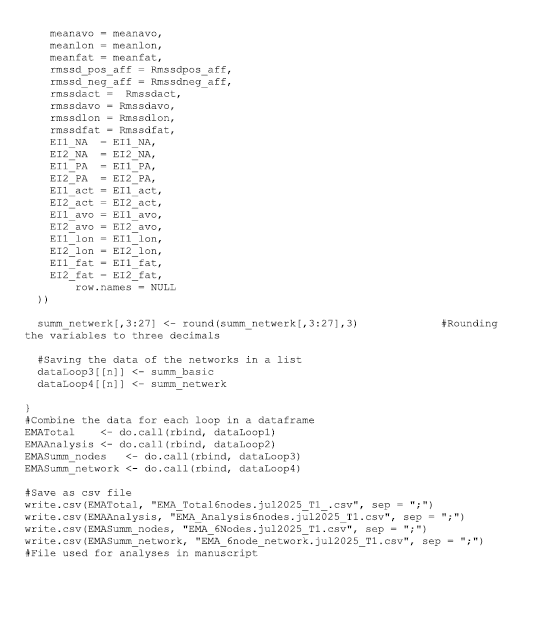


## Supplement 7 Symptom change

We have examined the depressive (Beck Depression Inventory; BDI) [18,19] and anxiety symptoms (Revised Child Anxiety and Depression Scale; RCADS) [20,21] data in the total sample and separately for both groups (Modules vs. No modules).

First, two line graphs are provided to visualize the change over time in depressive and anxiety symptoms within the total sample (Figures S5 and S6).

**Fig. S5**

Change from T0 to T1 in depressive symptoms


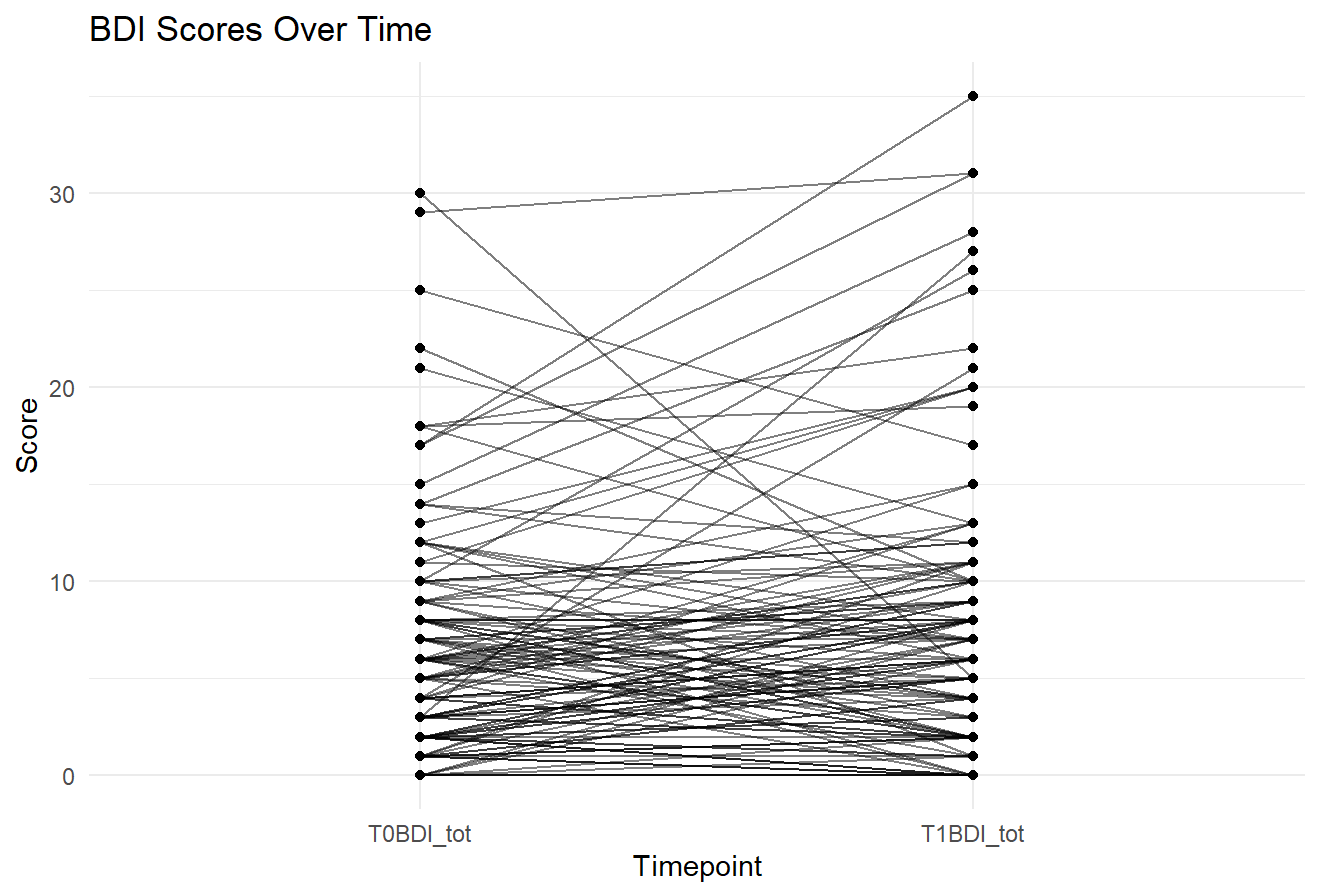


*Note*. Lines depict individuals within the total sample, remitted at baseline (*n* = 132).

**Fig. S6**

Change from T0 to T1 in anxiety symptoms


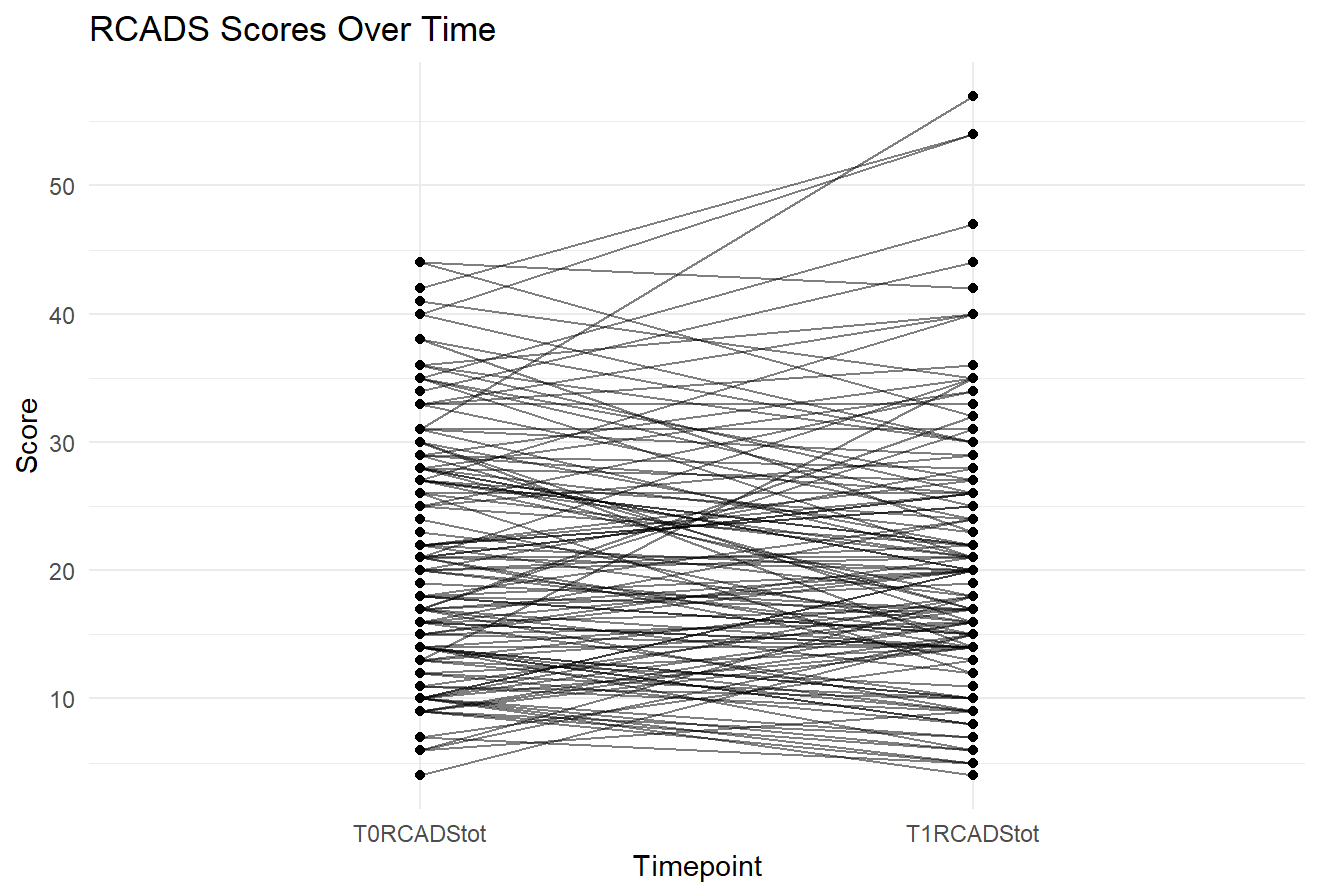


*Note*. Lines depict individuals within the total sample, remitted at baseline (*n* = 132).

In this remitted group, the symptom severity increased slightly from baseline to post-intervention for BDI (*n* = 132, β = 0.692, CI95% [0.000, 1.385], p < 0.001, R^2^ = .31), and for RCADS (n= 132, β = 0.789, CI95% [0.000, 1.578], *p* < 0.001, R^2^ = .51). Within the intervention group, symptom changes from 0 to 4 months were not associated with the number of modules (BDI change: β = 0.06418, CI95% [0.000, 0.128], *p* = 0.887, RCADS change: β = 0.5266, CI95% [-0.000, 1.053], *p* = 0.350).

Using Welch Two Sample t-tests, we examined difference between randomization groups in depressive and anxiety symptom changes. There were no statistically meaningful differences between groups (t_depression_[129.96] = 0.091, *p* = 0.928, M_CAU_ = 1.246, M_M+CAU_ = 1.149, and t_anxiety_[130] = 0.730, *p* = 0.466, M_CAU_ = 0.200, M_M+CAU_ =-0.761). Thus, in the current sample, randomization does not (yet) have predictive value on change in symptoms at four months.

These additional analyses suggest that although there is clinical change over time in symptoms of depression and anxiety, this change is not reflected in network change. BDI and RCADS data were missing for 2 out of 69 participants in the M+CAU group and 3 out of 68 in the CAU group, and that timing of the T1 assessment for the online questionnaires and EMA did not always overlap.

Lastly, we performed a regression analysis to examine baseline symptom severity in relation to completed intervention-modules. The number of completed intervention-modules was not associated with baseline depressive (BDI) or anxiety (RCADS) symptom severity. (n = 132, β = 0.094, CI95% [0.000, 0.188], *p* = 0.818), and for RCADS (n= 132, β = 0.049, CI95% [0.000, 0.098], *p* = 0.941). Thereby suggesting that baseline symptom severity does not positively or negatively correlate with completion of intervention-modules in this remitted group.

# References

[1] H. Wickham, R. François, L. Henry, K. Müller, D. Vaughan, dplyr: A grammar of data manipulation, Https://Dplyr.Tidyverse.Org, Https://Github.Com/Tidyverse/Dplyr. (2023).

[2] G. Grolemund, H. Wickham, Dates and times made easy with lubridate, J. Stat. Softw. 40 (2011) 1–25.

[3] B.D. Ripley, W.N. Venables, D.M. Bates, K. Hornik, A. Gebhardt, D. Firth, MASS: Support Functions and Datasets for Venables and Ripley’s MASS, R Top. Doc. (2023).

[4] S. Epskamp, A.O.J. Cramer, L.J. Waldorp, V.D. Schmittmann, D. Borsboom, qgraph: Network visualizations of relationships in psychometric data, J. Stat. Softw. 48 (2012) 1–18. https://doi.org/10.18637/jss.v048.i04.

[5] H. Wickham, J. Bryan, readxl: Read Excel files, Https://Readxl.Tidyverse.Org, Https://Github.Com/Tidyverse/Readxl. (2023).

[6] D. Hervas Marin, repmod: Create Report Table from Different Objects, (2021).

[7] H. Wickham, Reshaping data with the reshape package, J. Stat. Softw. 21 (2007) 1–20.

[8] A. Kassambara, rstatix: Pipe-Friendly Framework for Basic Statistical Tests, (2023).

[9] H. Wickham, M. Averick, J. Bryan, W. Chang, L. McGowan, R. François, G. Grolemund, A. Hayes, L. Henry, J. Hester, M. Kuhn, T. Pedersen, E. Miller, S. Bache, K. Müller, J. Ooms, D. Robinson, D. Seidel, V. Spinu, K. Takahashi, D. Vaughan, C. Wilke, K. Woo, H. Yutani, Welcome to the Tidyverse, J. Open Source Softw. 4 (2019). https://doi.org/10.21105/joss.01686.

[10] M. Mahmoudian, varhandle: Functions for robust variable handling, (2020).

[11] J.F. Wiley, E.G. Limited, varian: Variability Analysis in R, (2016).

[12] J.M. van der Wal, C.D. van Borkulo, J.M.B. Haslbeck, C. Slofstra, N.S. Klein, T.F. Blanken, M.K. Deserno, A. Lok, M.H. Nauta, C.L.H. Bockting, Differential impact of preventive cognitive therapy while tapering antidepressants versus maintenance antidepressant treatment on affect fluctuations and individual affect networks and impact on relapse: a secondary analysis of a randomised controlled trial, EClinicalMedicine 66 (2023) 102329. https://doi.org/10.1016/j.eclinm.2023.102329.

[13] C.K.F. Wen, S. Schneider, A.A. Stone, D. Spruijt-Metz, Compliance with mobile ecological momentary assessment protocols in children and adolescents: A systematic review and meta-analysis, J. Med. Internet Res. 19 (2017) e132. https://doi.org/10.2196/jmir.6641.

[14] E.T. Dobson, P.E. Croarkin, H.K. Schroeder, S.T. Varney, S.A. Mossman, K. Cecil, J.R. Strawn, Bridging anxiety and depression: A network approach in anxious adolescents, J. Affect. Disord. 280 (2021) 305–314. https://doi.org/10.1016/j.jad.2020.11.027.

[15] S. Epskamp, D. Borsboom, E.I. Fried, Estimating psychological networks and their accuracy: A tutorial paper, Behav. Res. Methods 50 (2018) 195–212. https://doi.org/10.3758/s13428-017-0862-1.

[16] B.E.A.M. Kooiman, S.J. Robberegt, C.J. Albers, C.L.H. Bockting, Y.A.J. Stikkelbroek, M.H. Nauta, Congruency of multimodaldata-driven personalization withshared decision-making forStayFine: Individualizedapp-based relapse prevention foranxiety and depression in youngpeople, Front. Psychiatry (2023) 1229713. https://doi.org/10.3389/fpsyt.2023.1229713.

[17] A.C. Mansueto, R.W. Wiers, J.C.M. van Weert, B.C. Schouten, S. Epskamp, Investigating the feasibility of idiographic network models, Psychol. Methods 28 (2023) 1052–1068. https://doi.org/10.1037/met0000466.

[18] A.T. Beck, C.H. Ward, M. Mendelson, J. Mock, J. Erbaugh, An inventory for measuring depression, Arch. Gen. Psychiatry 4 (1961) 561–571. https://doi.org/10.1001/archpsyc.1961.01710120031004.

[19] A.J.W. van der Does, BDI-NL. Handleiding bij de Nederlandse versie van de Beck Depression Inventory, (2002).

[20] A.J. Oldehinkel, Nederlandstalige vertaling van de Revised Child Anxiety and Depression Scale (RCADS), (2000).

[21] B.F. Chorpita, L. Yim, C. Moffitt, L.A. Umemoto, S.E. Francis, Assessment of symptoms of DSM-IV anxiety and depression in children: A revised child anxiety and depression scale, Behav. Res. Ther. 38 (2000) 835–855.
